# Rearrangements of organic peroxides and related processes

**DOI:** 10.3762/bjoc.12.162

**Published:** 2016-08-03

**Authors:** Ivan A Yaremenko, Vera A Vil’, Dmitry V Demchuk, Alexander O Terent’ev

**Affiliations:** 1 N. D. Zelinsky Institute of Organic Chemistry, Russian Academy of Sciences, Leninsky Prospect 47, Moscow, 119991, Russia

**Keywords:** artemisinin, Baeyer−Villiger, Criegee, Hock, peroxide, rearrangement

## Abstract

This review is the first to collate and summarize main data on named and unnamed rearrangement reactions of peroxides. It should be noted, that in the chemistry of peroxides two types of processes are considered under the term rearrangements. These are conventional rearrangements occurring with the retention of the molecular weight and transformations of one of the peroxide moieties after O–O-bond cleavage. Detailed information about the Baeyer−Villiger, Criegee, Hock, Kornblum−DeLaMare, Dakin, Elbs, Schenck, Smith, Wieland, and Story reactions is given. Unnamed rearrangements of organic peroxides and related processes are also analyzed. The rearrangements and related processes of important natural and synthetic peroxides are discussed separately.

## Introduction

The chemistry of organic peroxides has more than a hundred-year history. Currently, organic peroxides are widely used as oxidizing agents and initiators for free-radical reactions both in industry and in laboratory. These compounds are produced and involved in various natural and biological processes and were explored extensively as antimalarial agents, anthelmintics, and anticancer drugs.

Organic peroxides, such as alkyl hydroperoxides, aryl hydroperoxides, ketone peroxides, dialkyl peroxides, diacyl peroxides, peroxy esters, peroxydicarbonates, peroxyacetals, and inorganic peroxides are the most important radical initiators that are widely used in industrial processes in the manufacture of polymers from unsaturated monomers [1–9].

Nowadays, the progress in the chemistry of organic peroxides is mainly a result of their biological activity and pharmaceutical application. The search of effective antimalarial and antihelminthic drugs is the main challenge of medicinal chemistry of peroxides. According to the World Health Organization (WHO) malaria is a widely distributed illness. About 3.2 billion people remain at risk of malaria and in 2015 214 million cases of malaria and 438 thousands deaths from it have been registered [10]. Compounds with high antimalarial [11–23], antihelminthic [24–28], and antitumor activities [29–34] were found among natural, semisynthetic, and synthetic peroxides. The main biologically active frame of these compounds includes five-membered 1,2-dioxolane [35–37], 1,2,4-trioxolane [38–39], and six-membered 1,2-dioxane [40–42], 1,2-dioxene [43], 1,2,4-trioxane [22,44–45] cycles. The naturally occuring peroxide artemisinin and its semisynthetic derivatives, artemether, arteether, and artesunate, are applied in large scale for malaria treatment [46–47].

Organic peroxides, their rearrangements and related processes play an important role in the chemistry of oxidation processes. Thus, the key reagent in the Sharpless epoxidation of allylic alcohols [48] and in the manufacture of propylene oxide via the Prilezhaev reaction [49–51] is *tert*-butyl hydroperoxide. In industry, phenol and acetone are mainly produced by the Hock process, which is based on the rearrangement of cumene hydroperoxide. In 2003, phenol was produced to more than 95% by this oxidation process [52–54]. Another important application of organic peroxides is the synthesis of lactones from cyclic ketones via the Baeyer−Villiger oxidation and it is one of the methods for the synthesis of commercially important caprolactone from cyclohexanone with peracetic acid [55–56].

Autoxidation processes with formation of hydroperoxides and their subsequent free-radical transformations with generation of carbon- and oxygen-centered radicals are key reactions in the drying process of oil-based and alkyd paints containing double bonds [57–61].

Organic peroxides and their transformation play an important role not only in industrial but also in biological processes. Thus, the firefly luciferase-catalyzed oxidation of luciferin yields the peroxy compound 1,2-dioxetane. This four-membered peroxide cycle is unstable and spontaneously decays to carbon dioxide and excited ketones, which release excess energy through light emission (bioluminescence) [62–65]. The in vivo oxidation of cholesterol by singlet oxygen produces the hydroperoxide cholesterol-5α-OOH, which undergoes a Hock oxidation to form atheronal A. The latter possesses proatherogenic effects and triggers the development of cardiovascular diseases [66–71].

The development of the chemistry of organic peroxides is closely related to the application and preparation of unsaturated compounds, such as epoxides, aldehydes, ketones, carboxylic acids, and their derivatives [72–113]. Organic peroxides are widely used as oxidants in oxidative coupling processes [114–120].

Industrial-scale production of readily available and efficient initiators of free radical polymerization and effective biologically active compounds promotes the search for new synthetic methods for peroxides starting from carbonyl compounds, hydrogen peroxide, and hydroperoxides [121–182].

In many cases, rearrangements and related reactions of peroxides are key pathways in laboratory, industrial, and biological processes. The rearrangements of organic peroxides are covered in the literature in hundreds of publications and in several specialized and partial reviews [183–188]. The present review is the first to combine the key data on both, name rearrangements and less well-known rearrangements and related oxidative processes, and to summarize systematically related and different features of these reactions, compares their mechanisms, and assesses the prospects of their application.

By definition, a rearrangement is a migration of an atom or a group of atoms from one atom to another within the same molecule [189]. In contrast, a rearrangement of organic peroxides means a change in the structure of the starting molecule to form an isomeric compound without a peroxy group [183]. The terminology of rearrangements of organic peroxides and related processes encountered in the literature shows that this definition is not generally applicable as rearrangements of peroxides can give both isomeric and non-isomeric compounds either containing a peroxy group or without the latter. In most cases, a rearrangement involves the migration or cleavage of the peroxide group in an intermediate molecule, and the stability of the latter is responsible for the further pathway of the process.

**Figure 1:**
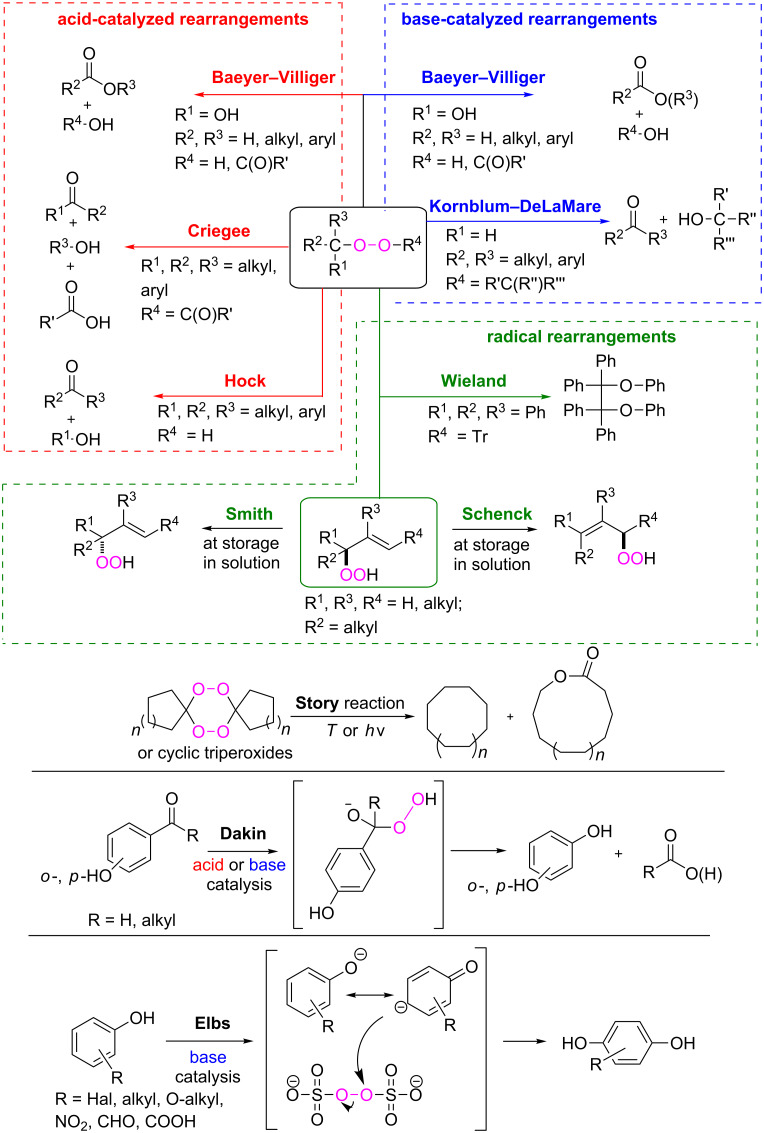
The named transformations considered in this review.

The review covers main studies published over the last 15–20 years with a brief excursion to the history of the development of various reactions and transformations. The review consists of three parts: the first part considers named transformations of organic peroxides (Figure 1), the second one deals with unnamed reactions, and the third part covers transformations of some important natural and synthetic peroxides. Since the term “rearrangements”, as applied to transformations of peroxides, is not clearly defined all parts of the review include processes related to rearrangements.

## Review

### 1 Named rearrangements of organic peroxides

Rearrangements of organic peroxides are the key steps in many well-known processes such as the Baeyer–Villiger (BV), the Criegee and Hock reactions, the Kornblum–DeLaMare rearrangement, Dakin, and Elbs oxidation.

The BV oxidation is widely used in organic synthesis for the preparation of esters and lactones and the Criegee reaction is applied to transform tertiary alcohols into ketones and aldehydes. The Hock rearrangement is a key step in the cumene (cumene–phenol) process and the Kornblum–DeLaMare is an important tool in the synthesis of functionalized ketones and alcohols, including γ-hydroxy enones. The Dakin oxidation finds application for the synthesis of phenols from arylaldehydes or aryl ketones and the Elbs persulfate oxidation allows the preparation of hydroxyphenols from phenols. Finally, the Schenck and Smith rearrangements are of interest in allyl hydroperoxide transformations.

#### 1.1 Baeyer–Villiger oxidation

**Scheme 1:**
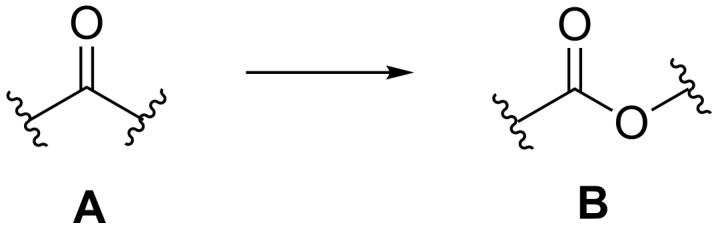
The Baeyer–Villiger oxidation.

The BV reaction is the oxidation of ketones or aldehydes **A** under the action of hydrogen peroxide, hydroperoxides, Caro’s acid (H_2_SO_5_), or organic peracids to yield esters, lactones, or carboxylic acids **B** (Scheme 1) [190–191].

Baeyer and Villiger accomplished the oxidation of ketones to esters for the first time in 1899 while they attempted the reaction of Caro’s acid (H_2_SO_5_) with menthone, tetrahydrocarvone, and camphor to transform these compounds into the corresponding lactones [192–194].

Since that time, this reaction has shown to be of general applicability and it has gained wide application for the oxidation of carbonyl compounds of different structures. In this reaction, cyclic ketones are transformed into lactones, acyclic ketones, into esters and aldehydes into carboxylic acids. The BV oxidation is one of the most important reactions in organic chemistry because it produces lactones, which are useful synthetic products in polymer, agrochemical, and pharmaceutical industry.

*m*-Chloroperbenzoic, peracetic, and perfluoroacetic acids, as well as hydrogen peroxide/protic acid, hydrogen peroxide/Lewis acid, and hydrogen peroxide/base systems are widely employed in the Baeyer–Villiger oxidation [185,194–195].

**Scheme 2:**
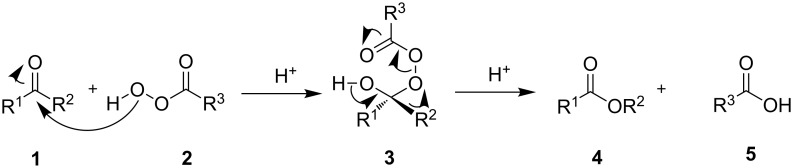
The general mechanism of the peracid-promoted Baeyer–Villiger oxidation.

The general mechanism of the peracid-promoted Baeyer–Villiger oxidation involves two main steps. In the first step, the oxygen atom of the peroxide moiety of the peracid **2** binds to the carbonyl group of ketone **1** to form the tetrahedral intermediate **3** which is referred to as the Criegee intermediate. The next step involves the concerted migration of the R^2^ group to the peroxide oxygen atom, resulting in the formation of ester **4** and carboxylic acid **5** (Scheme 2).

The ability of peracids to oxidize cyclic and acyclic ketones and aldehydes to the corresponding lactones, esters, and carboxylic acids decreases in the series peroxotrifluoroacetic acid > monopermaleic acid > mono-*o*-perphthalic acid > 3,5-dinitroperbenzoic acid > *p*-nitroperbenzoic acid > MCPBA ≈ performic acid > perbenzoic acid > peracetic acid >> H_2_O_2_ > *t*-BuOOH [196].

The migratory ability of substituents in the Criegee intermediate decreases in the following series: tertiary alkyl > cyclohexyl > secondary alkyl > benzyl > phenyl > primary alkyl > cyclopentyl, cyclopropyl > methyl. In some cases, stereoelectronical effects strongly influence the regioselectivity of the reaction, specifically the ability of the migrating C–C to align with the back of the breaking O–O bond, and the presence or absence of strain in cyclic ketone substrates [197–198]. The strongest electron-donating group migrates in unsymmetrical ketones [199].

There are thousands of publications on the Baeyer–Villiger reaction. In the latest reviews published by Krow [195] in 1993 and by Renz and Meunier [185] in 1999, the field of application, the reactivity of substrates, and the reaction kinetics and mechanisms are considered in detail. In the review by Strukul, special emphasis was placed on metal-catalyzed Baeyer–Villiger oxidations [196]. Green approaches in the Baeyer–Villiger reaction were highlighted by another review [200].

**Scheme 3:**
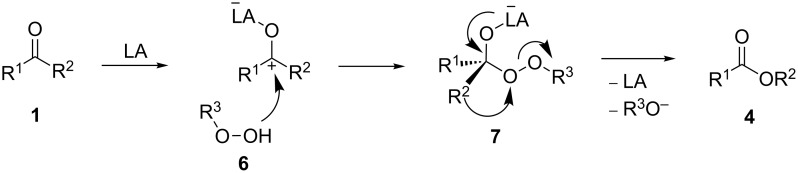
General mechanism of the Lewis acid-catalyzed Baeyer–Villiger rearrangement.

The present review covers a more modern aspect of this reaction, viz., the performance of the process using hydrogen peroxide. Oxidizing systems containing hydrogen peroxide as the oxidizing agent allow the usual and asymmetric oxidation of the substrate to the target product with high conversion and yield. In recent years, the inexpensive, commercially available, and environmentally friendly H_2_O_2_ was utilized in the Baeyer–Villiger reaction with increasing frequency. Various catalysts that activate hydrogen peroxide, such as heterogeneous catalysts based on solid acids [201], zeolites [202–203], Se [204], As [205], Co [206], sulfonated organic ion exchange resins [203,207], and homogeneous catalysts based on Pt [208], Zr [209], Re [210–211], Se [212–213], As [205], Mo [214], Co [215], Brønsted [216], and Lewis acids [217] are described in the literature. The general mechanism of a Lewis acid-catalyzed Baeyer–Villiger rearrangement is presented in Scheme 3 [200,218].

**Scheme 4:**
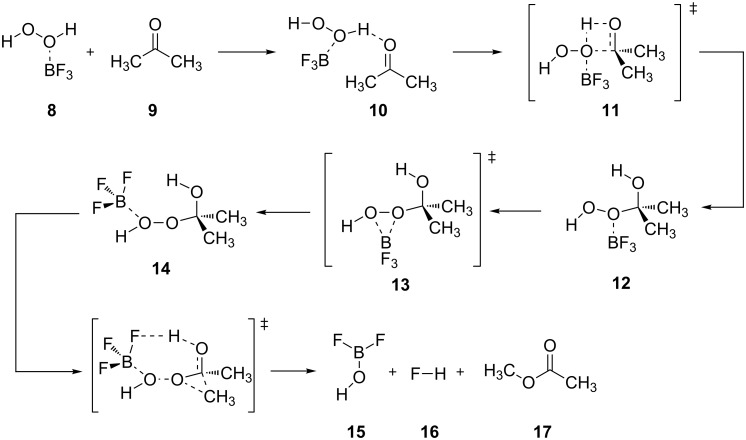
The theoretically studied mechanism of the BV oxidation reaction promoted by H_2_O_2_ and the Lewis acid BF_3_.

Scheme 4 shows the theoretically studied mechanism of the oxidation reaction promoted by H_2_O_2_ and the Lewis acid BF_3_ [217,219]. In the first step, the hydrogen peroxide–boron trifluoride complex **8** reacts with ketone **9** to form adduct **10**. The latter intermediate rearranges through transition state **11** into the tetrahedral peroxyacetal intermediate **12**. Then BF_3_ migrates to another oxygen atom through transition state **13** to give the second Criegee intermediate **14**. The decomposition of intermediate **14** finally produces **15**, hydrogen fluoride (**16**) and ester **17**.

**Scheme 5:**
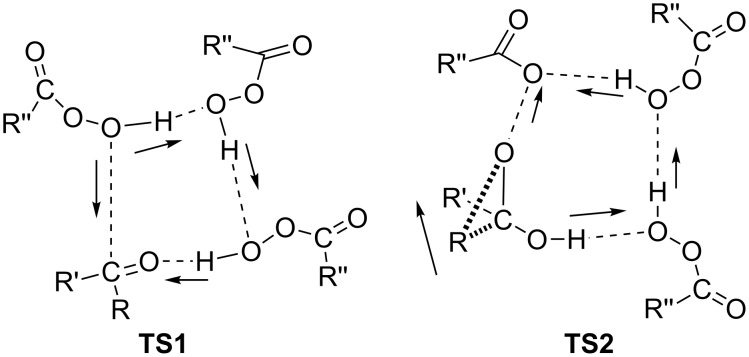
Proton movements in the transition states of the Baeyer–Villiger oxidation.

Despite the fact that the Baeyer–Villiger reaction is known since 1899, the mechanism of this reaction is still not fully understood. The nature of the acid catalyst [220] and the type of O–O-bond cleavage in the Criegee intermediate [221] were found to play an important role in this reaction. Probably the hydrogen bonds in Baeyer–Villiger reactions play an important role [222]. The tetramolecular transition states TS1 and TS2 are considered to be the two key steps determining the course of the oxidation: the nucleophilic addition of a peroxy acid molecule to ketone (TS1) and the migration of R and cleavage of O–O bond (TS2). Thus, electrophilic substrates favor TS1 and nucleophilic migrating groups prefer TS2 (Scheme 5).

**Scheme 6:**
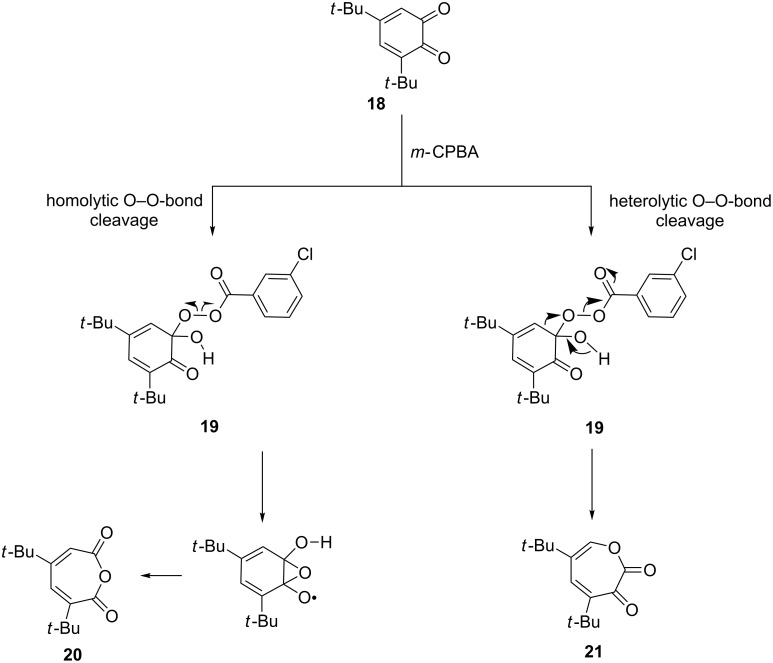
The dependence of the course of the Baeyer–Villiger oxidation on the type of O–O-bond cleavage in the Criegee intermediate.

The dependence of the course of the Baeyer–Villiger oxidation on the type of O−O-bond cleavage in the Criegee intermediate was studied in the oxidation reaction of 1,2-quinone **18** with perbenzoic acid [221]. The reaction gave two oxidation products – anhydride **20** and the seven-membered α-ketolactone **21**. The investigation of the reaction mechanism demonstrated that the formation of the seven-membered α-ketolactone **21** proceeds through the heterolytic O–O-bond cleavage in Criegee intermediate **19**, whereas the homolytic O–O cleavage affords anhydride **20** (Scheme 6).

**Scheme 7:**
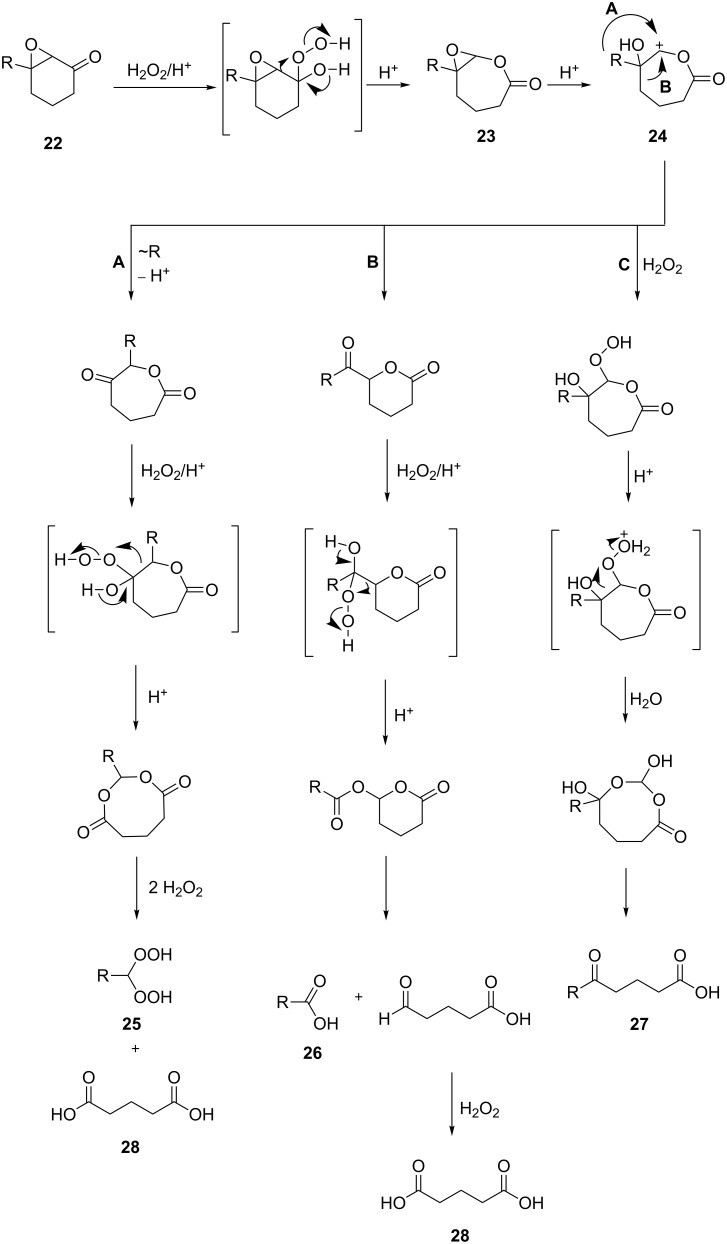
The acid-catalyzed Baeyer–Villiger oxidation of cyclic epoxy ketones **22**.

The acid-catalyzed Baeyer–Villiger oxidation of cyclic epoxy ketones **22** produces lactones of type **23**, which convert into carbenium ions **24** in the presence of the acid. Subsequently, these ions can be transformed with participation of H_2_O_2_ through three different pathways into dihydroperoxides **25**, dicarboxylic acids **28**, carboxylic acids **26**, and keto carboxylic acids **27** (Scheme 7, Table 1) [223].

**Table 1:** Oxidation of cyclic epoxy ketones **22a**–**c** by H_2_O_2_.

Epoxy ketone	R	**25**, %	**26**, %	**27**, %	**28**, %

**22a**	Me	**25a**, 12	**26a**, 6	**27a**, 15	**28a**, 53
**22b**	Et	**25b**, 19	^a^	^a^	^a^
**22c**	Ph	**25c**, 19	–	**27c**, 35	**28c**, 18

^a^The aqueous phase consisted of a complex mixture and could not be analyzed.

**Scheme 8:**
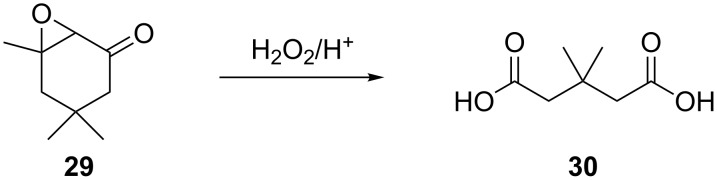
Oxidation of isophorone oxide **29**.

The oxidation of isophorone oxide (**29)** is an industrial process for the production of dimethylglutaric acid **30** (Scheme 8) [223].

**Scheme 9:**
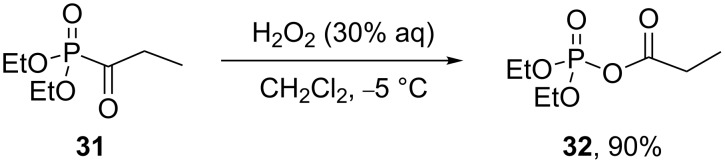
Synthesis of acyl phosphate **32** from acyl phosphonate **31**.

Acyl phosphate **32** can be synthesized from acyl phosphonate **31** in high yield by oxidation with H_2_O_2_ (Scheme 9) [224].

**Scheme 10:**
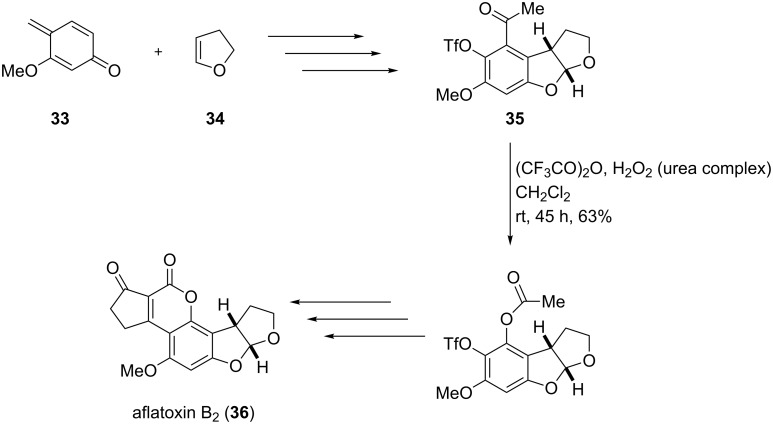
Synthesis of aflatoxin B_2_ **(36)**.

The Baeyer–Villiger oxidation provides a valuable tool for the synthesis of oxygenated natural products [218,225–226] as exemplified by the synthesis of aflatoxin B_2_ (**36**, Scheme 10) [227].

The Baeyer–Villiger reaction is also a key step in the multistep synthesis of cannabinergic lactones from dimethylheptylresorcinol. Two regioisomeric cannabinergic lactones were obtained, one of which possessed pronounced affinity towards the CB1 receptor and lower affinities for mCB2 and hCB2 receptors [228].

**Scheme 11:**
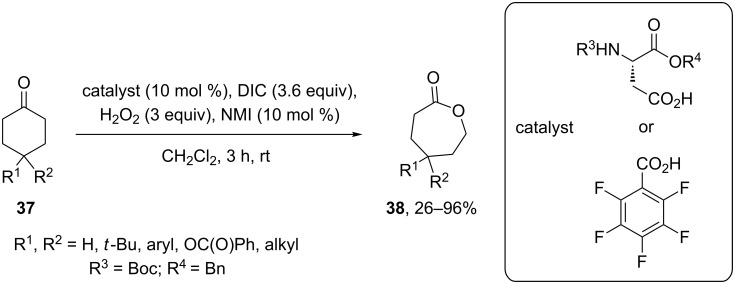
The Baeyer–Villiger rearrangement of ketones **37** to lactones **38**.

**Oxidation with H_2_O_2_–acid systems:** With in situ generated peracids from carbodiimide, hydrogen peroxide, and carboxylic acids as catalysts ketones **37** are rearranged to lactones **38** (Scheme 11) [229].

**Scheme 12:**
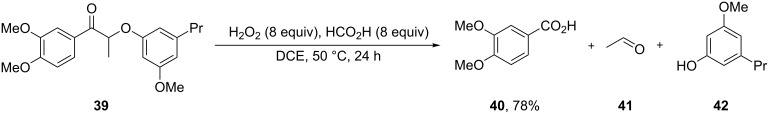
Synthesis of 3,4-dimethoxybenzoic acid (**40**) via Baeyer–Villiger oxidation.

3,4-Dimethoxybenzoic acid (**40**) was prepared with 78% yield by a Baeyer–Villiger reaction of substrate **39** with 30% H_2_O_2_, HCOOH and 1,2-dichloroethane at 50 °C for 24 h (Scheme 12) [230].

**Scheme 13:**
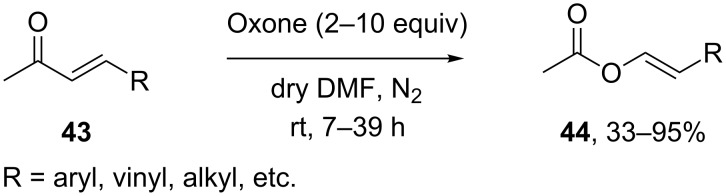
Oxone transforms α,β-unsaturated ketones **43** into vinyl acetates **44**.

Oxone is a convenient reagent for the transformation of α,β-unsaturated ketones **43** of determined stereochemistry into vinyl acetates **44** via the Baeyer–Villiger reaction in dry DMF for 7–39 h (Scheme 13) [231].

**Oxidation with H_2_O_2_–heteroorganic catalyst systems:** The activity of oxidizing systems such as H_2_O_2_/aryl benzyl selenoxide and H_2_O_2_/diaryl diselenide is similar to that of *m*-chloroperbenzoic acid [212,232–233]. The main advantage of these selenium-containing systems is that the catalysts are regenerated and can therefore be used at low loadings [234–236]. Some results of the oxidation of ketones and aldehydes **45a**–**c** to the corresponding esters **46a**–**c** using the H_2_O_2_/aryl benzyl selenoxide system are collected in Table 2 [232].

**Table 2:** Baeyer–Villiger oxidation of aldehyde **45a** and ketones **45b**,**c** using the H_2_O_2_/aryl benzyl selenoxide system.

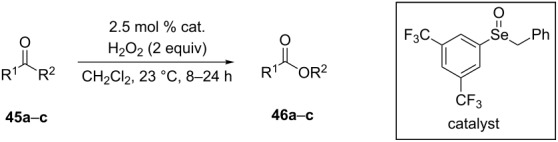			

Substrate	Time, h	Product	Yield, %

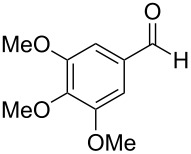 **45a**	8	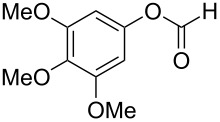 **46a**	96
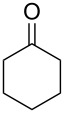 **45b**	24	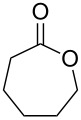 **46b**	94
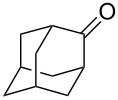 **45c**	18	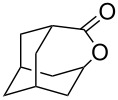 **46c**	98

The oxidation results of ketones **47a**,**b** and aldehydes **47c**–**e** to lactones **48a**,**b** and carboxylic acids **49a**–**c** promoted by the H_2_O_2_/diaryl diselenide system is presented in Table 3 [212].

**Table 3:** Baeyer–Villiger oxidation of ketones **47a**,**b** and aldehydes **47c**–**e** promoted by the H_2_O_2_/diaryl diselenide system.

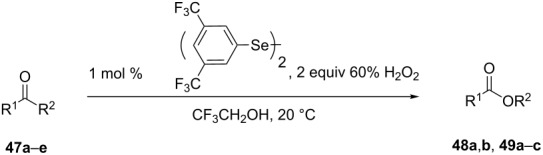				

Ketone	Time, h	Product	Conversion, %^a^	Selectivity (BV product), %^a^

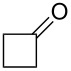 **47a**	1	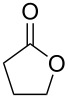 **48a**	99	90
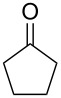 **47b**	8	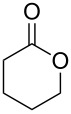 **48b**	95	94
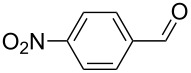 **47c**^b^	2	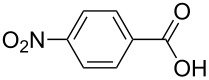 **49a**	98	98
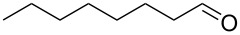 **47d**^b^	3	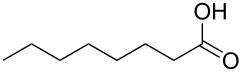 **49b**	88	96
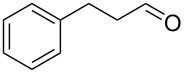 **47e**^b^	3	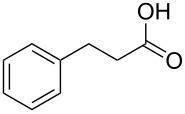 **49c**	>90	99

^a^Determined by GC; ^b^60 °C.

**Scheme 14:**
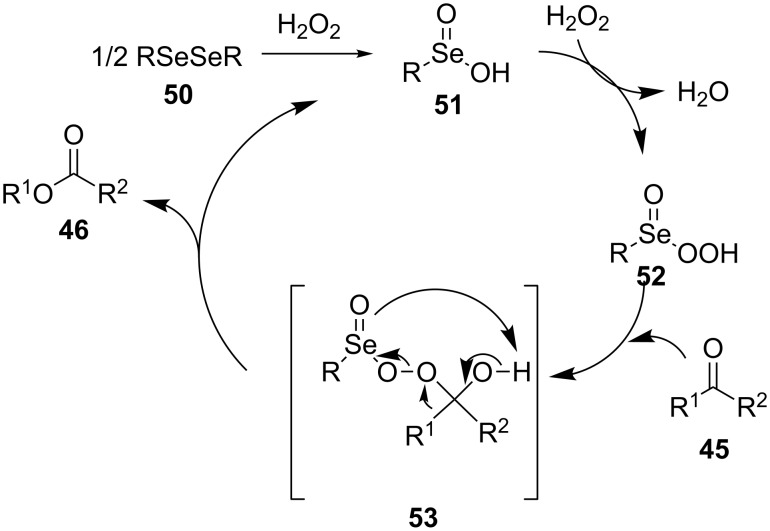
The Baeyer–Villiger oxidation of ketones **45** using diaryl diselenide and hydrogen peroxide.

In the first step of the catalytic cycle of the Baeyer–Villiger oxidation using diaryl diselenide **50** and hydrogen peroxide seleninic acid **51** is generated, which is then oxidized to perseleninic acid **52**. Oxidation of the ketone **45** by perseleninic acid **52** involves the intermediate peroxide **53** (Scheme 14) [235].

**Scheme 15:**
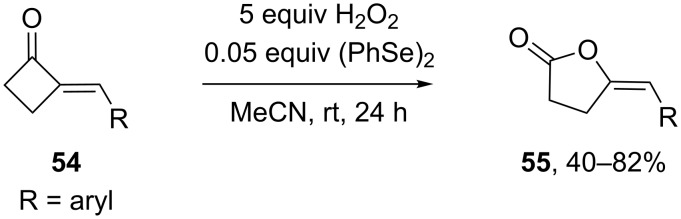
Baeyer–Villiger oxidation of (*E*)-2-methylenecyclobutanones.

Similarly, the versatile 4-methylenebutanolides **55** can be prepared from (*E*)-2-methylenecyclobutanones **54** in the presence of (PhSe)_2_/H_2_O_2_ at room temperature (Scheme 15). Likely the Baeyer–Villiger reaction proceeds through the formation of benzeneseleninoperoxoic anhydride [PhSe(O)O]_2_O in the first step, which then transforms to the active oxidant benzeneseleninoperoxoic acid PhSe(O)OOH [233].

**Scheme 16:**
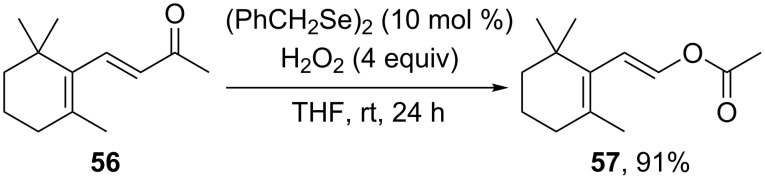
Oxidation of β-ionone (**56**) by H_2_O_2_/(BnSe)_2_ with formation of (*E*)-2-(2,6,6-trimethylcyclohex-1-en-1-yl)vinyl acetate (**57**).

The Baeyer–Villiger oxidation of (*E*)-α,β-unsaturated ketones to (*E*)-vinyl esters was performed with hydrogen peroxide and dibenzyl diselenide as pre-catalyst at room temperature [236]. Catalyzed by the dibenzyl diselenide, β-ionone (**56**) was oxidized by H_2_O_2_ with formation of (*E*)-2-(2,6,6-trimethylcyclohex-1-en-1-yl)vinyl acetate (**57**) with 91% yield (Scheme 16) [237].

The Baeyer–Villiger oxidation of ketones **58a**–**f** to form esters **59a**–**f** can be accomplished in good yields in the presence of H_2_O_2_ and arsenic-containing ion exchange resins on polystyrene as the catalyst (Table 4) [203,205].

**Table 4:** Oxidation of ketones **58a**–**f** with 90% H_2_O_2_ catalyzed by arsonated polystyrene.

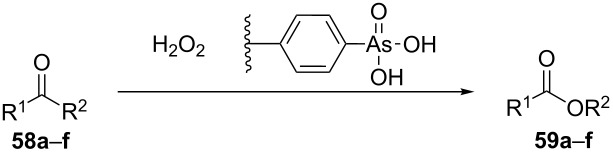				

Ketone	Time, h	Ketone/H_2_O_2_, ratio	Product	Yield based on the ketone consumed, %

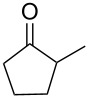 **58a**	11	1	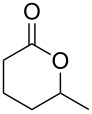 **59a**	92
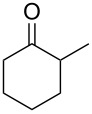 **58b**	5	5	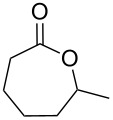 **59b**	100
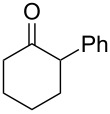 **58c**	15	1	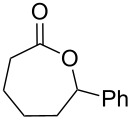 **59c**	100
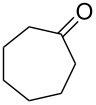 **58d**	23	5	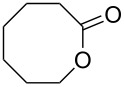 **59d**	29
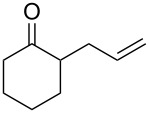 **58e**	9	1	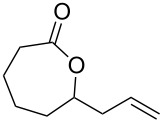 **59e**	70
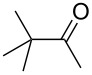 **58f**	25	5	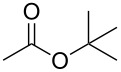 **59f**	100

**Scheme 17:**
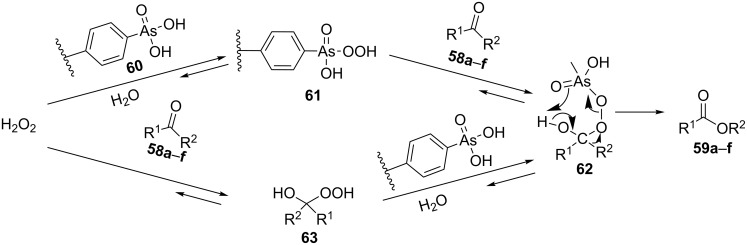
The mechanism of oxidation of ketones **58a**–**f** by hydrogen peroxide in the presence of arsonated polystyrene **60**.

The mechanism of the oxidation of ketones **58a**–**f** by hydrogen peroxide in the presence of arsonated polystyrene **60** as the catalyst is shown in Scheme 17. First, hydrogen peroxide reacts with the arsonic acid **60** to form peroxyarsonic acid **61** or it adds to ketones **58a**–**f** to form vicinal hydroperoxyalkanols **63**. In the second step the peroxyarsonic acid **61** adds to ketones **58a**–**f** or the vicinal hydroperoxyalkanols **63** interact with arsonated polystyrene **60** under formation of perester **62**. Finally, the decomposition of **62** gives esters **59a**–**f**.

A number of other modern oxidizing systems are based on transition metal-peroxo complexes. The use of transition metal complexes were also used as catalysts for the Baeyer–Villiger reaction and the first example was documented in 1978 [196,214]. For example, Mo(VI) peroxo complexes **64** and **65** were employed as the catalysts and 90% H_2_O_2_ served as the oxidizer (Table 5).

**Table 5:** Oxidation of cyclic ketones **45b**, **47b** and **58a**,**b** by H_2_O_2_ in the presence of Mo(VI) peroxo complex **64** as the catalyst.

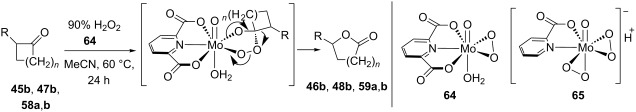		

Ketone	Product	Yield, %

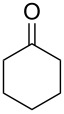 **45b**	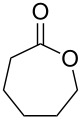 **46b**	10
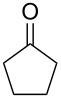 **47b**	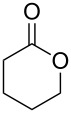 **48b**	40
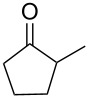 **58a**	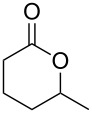 **59a**	82
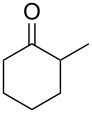 **58b**	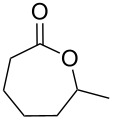 **59b**	10

**Scheme 18:**
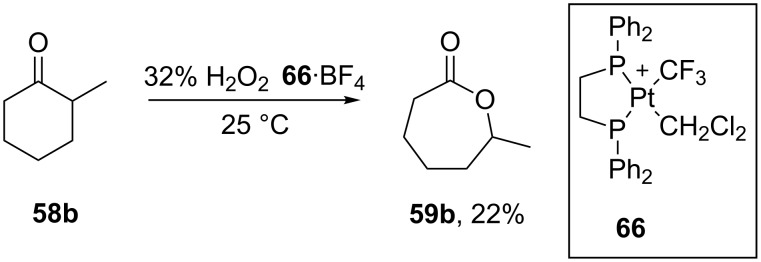
Oxidation of ketone (**58b**) by H_2_O_2_ to 6-methylcaprolactone (**59b**) catalyzed by Pt complex **66**·BF_4_.

The results obtained from the reactions using molybdenum systems have stimulated the search for new catalysts based on transition metal complexes. The usage of the platimum complex [(dppe)Pt(CF_3_(CH_2_Cl_2_)]BF_4_ (**66**·BF_4_) allowed the oxidation of 2-methylcyclohexanone (**58b**) in the presence of 32% H_2_O_2_ at room temperature to form 6-methylcaprolactone (**59b**) in 22% yield (Scheme 18) [238].

**Scheme 19:**
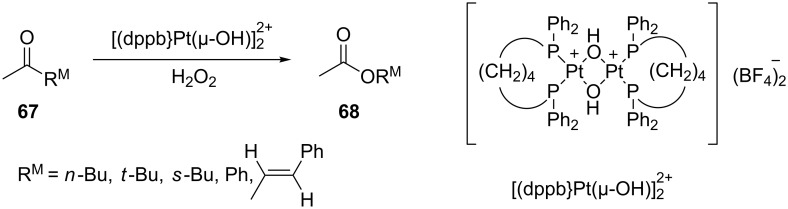
Oxidation of ketones **67** with H_2_O_2_ in the presence of [(dppb}Pt(µ-OH)]_2_^2+^.

Acyclic ketones **67** could be oxidized to the corresponding esters **68** in the presence of the catalyst [(dppb}Pt(µ-OH)]_2_^2+^, where dppb is butane-1,4-diylbis(diphenylphosphane) (Scheme 19) [208].

**Scheme 20:**
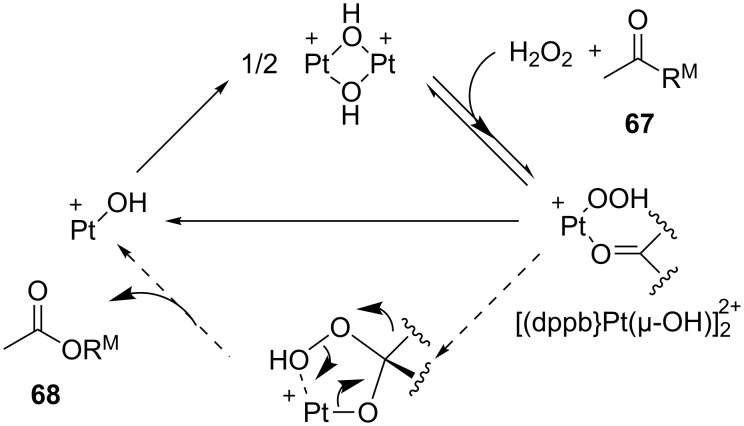
The mechanism of oxidation of ketones **67** in the presence of [(dppb}Pt(µ-OH)]_2_^2+^ and H_2_O_2_.

The oxidation mechanism of ketones **67** is displayed in Scheme 20.

The use of variable-valence metal complexes opened up a new field of application of the Baeyer–Villiger oxidation and there are now dozens of studies on this topic [196,239–246].

**Scheme 21:**
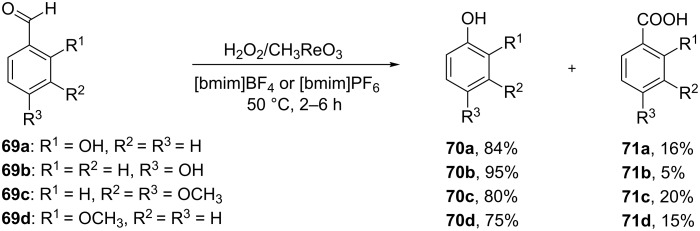
Oxidation of benzaldehydes **69** in the presence of the H_2_O_2_/MeReO_3_ system.

**Scheme 22:**
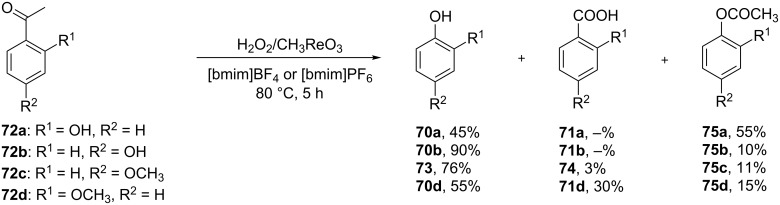
Oxidation of acetophenones **72** in the presence of the H_2_O_2_/MeReO_3_ system.

Hydroxylated and methoxylated benzaldehydes **69** (Scheme 21) and acetophenones **72** (Scheme 22) can be oxidized to the corresponding phenols **70a**–**d**, and **73** in good yields in the presence of the H_2_O_2_/MeReO_3_ system in ionic liquids [bmim]BF_4_ or [bmim]PF_6_ [247]. Benzoic acids **71a**–**d**, **74** and phenyl esters **75a**–**d** were reported as oxidation byproducts.

**Scheme 23:**
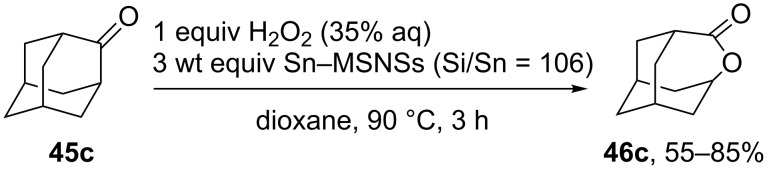
Baeyer–Villiger oxidation of 2-adamantanone (**45c**) in the presence of Sn-containing mesoporous silica nanospheres (Sn-MSNSs).

Sn-containing mesoporous silica nanospheres (Sn-MSNSs) with uniform crater-like mesopores exhibited high activities in the Baeyer–Villiger oxidation of 2-adamantanone (**45c**) (Scheme 23) [248].

The Baeyer–Villiger rearrangement of 2-adamantanone (**45с**) was performed using hydrogen peroxide (H_2_O_2_) and stannosilicate zeolites with nanosheet morphology and MFI topology (Sn-MFI-ns) as highly efficient catalysts [249]. The Sn-beta zeolites prepared by a steam-assisted conversion method are efficient catalysts for the Baeyer–Villiger reaction of cyclohexanone to ε-caprolactone [250]. A mesoporous Mg–Al-mixed oxide showed good catalytic efficiency in the Baeyer–Villiger oxidation of a series of ketones to the corresponding lactones and esters in the presence of diluted aqueous H_2_O_2_ and benzonitrile [251].

**Scheme 24:**
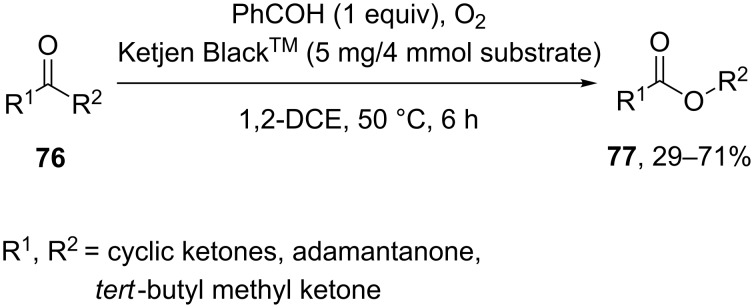
Aerobic Baeyer–Villiger oxidation of ketones **76** using metal-free carbon.

The Baeyer–Villiger oxidation of ketones **76** under the action of oxygen to the related esters **77** was performed using metal-free carbon (Ketjen Black) as a solid catalyst and benzaldehyde as the sacrificing agent. This metal-free carbon catalyst showed excellent catalytic activity and can be recycled after the reaction under oxygen atmosphere at 50 °C (Scheme 24) [252].

The boron-containing catalysts LiB(C_6_F_5_)_4_ or Ca[B(C_6_F_5_)_4_]_2_ were developed for the Baeyer–Villiger oxidation of ketones with aqueous H_2_O_2_ to give the lactones in high yields [253–254].

**Scheme 25:**
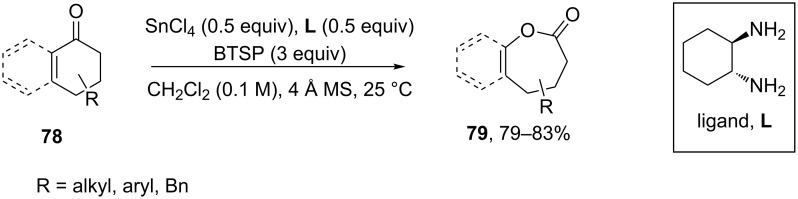
A regioselective Baeyer-Villiger oxidation of functionalized cyclohexenones **78** into a dihydrooxepine structures **79**.

A regioselective Baeyer–Villiger oxidation of functionalized cyclohexenones **78** lead to dihydrooxepine structures **79**. Here, the combination of SnCl_4_ and bis(trimethylsilyl)peroxide (BTSP), in the presence of *trans*-1,2-diaminocyclohexane as the ligand, generated the desired products **79** in high yields (Scheme 25) [255].

**Scheme 26:**
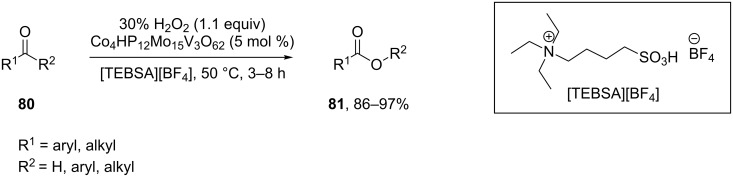
The oxidation of aldehydes and ketones **80** by H_2_O_2_ catalyzed by Co_4_HP_2_Mo_15_V_3_O_62_.

The Co_4_HP_2_Mo_15_V_3_O_62_-catalyzed oxidation of aldehydes and ketones **80** by hydrogen peroxide in ionic liquid [TEBSA][BF_4_] resulted in carboxylic acids and esters **81** in good to high yields (Scheme 26) [256].

**Scheme 27:**
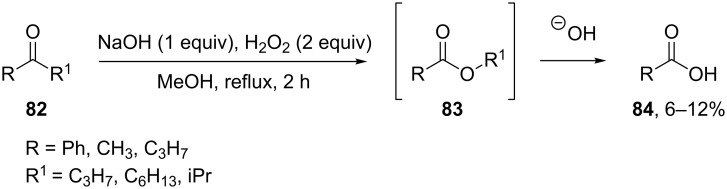
The cleavage of ketones **82** with hydrogen peroxide in alkaline solution.

**Oxidation with H_2_O_2_–base systems:** The oxidative cleavage of ketones **82** with hydrogen peroxide in alkaline solution yielded carboxylic acids **84**. The authors suggested that the reaction of a ketone with the hydroperoxide anion resulted in the intermediate esters **83**, which hydrolyzed in the basic reaction medium with formation of acids **84** (Scheme 27) [257].

**Scheme 28:**
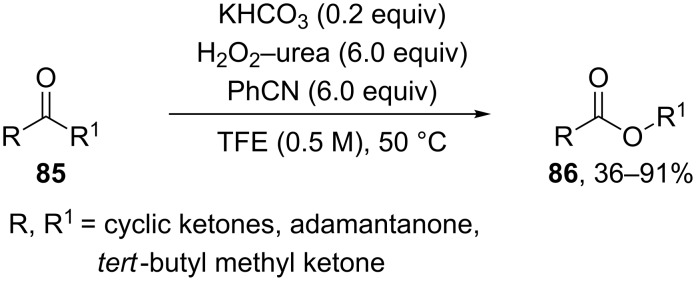
Oxidation of ketones **85** to esters **86** with H_2_O_2_–urea in the presence of KHCO_3_.

The use of hydrotalcites in the Baeyer–Villiger oxidation of various ketones resulted in high yields of the corresponding lactones or esters [258–260]. The esters **86** were synthesized by the reaction of ketones **85** with H_2_O_2_ and benzonitrile under basic reaction conditions (KHCO_3_) with the intermediate generation of peroxyimidic acids. This oxidation can be successfully applied to alkyl-containing ketones to give the target products in yields of 30–91% and good regioselectivity 7:1 to 20:1 (Scheme 28) [261].

**Asymmetric oxidation:** Asymmetric Baeyer–Villiger oxidation reactions can be performed using chiral acetals, organic hydroperoxides, chiral metal complexes and organocatalysts [262–263]. There are also Green chemistry approaches for Baeyer−Villiger oxidations based on enzyme-mediated processes, which are used for the preparation of chiral lactones. This type of biocatalysis is useful in synthetic chemistry and either isolated enzymes or living whole cells are applied for the oxidative production of valuable intermediates [264–269].

The asymmetric oxidation of 3-substituted cyclopentane-1,2-diones **87a**–**f** is an efficient tool in organic synthesis for the preparation of unsymmetrical γ-lactone acids **88a**–**f** with high optical purity and good yields (Table 6). These γ-lactone acids are valuable substrates for the synthesis of compounds with potentially useful pharmacological properties, such as homocitrates, alkyl- and aryl-substituted nucleosides [270–272].

**Table 6:** Asymmetric oxidation of 3-substituted cyclopentane-1,2-diones **87a**–**f** with the Ti(O-iPr)_4_/(+)DET/*t*-BuOOH system.

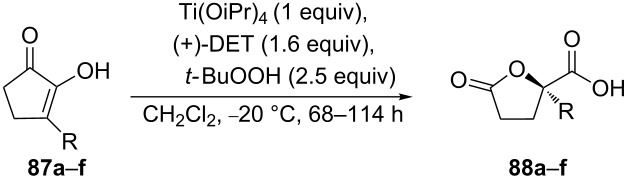			

γ-Lactone acid	R	Yield, %	ee, %

**88a**	-CH_3_	75	93
**88b**	-C_2_H_5_	72	93
**88c**	-CH_2_-OBn	75	96
**88d**	-Bn	83	96
**88e**	-C_6_H_5_	38	86
**88f**	4-F-C_6_H_4_-	43	86

**Scheme 29:**
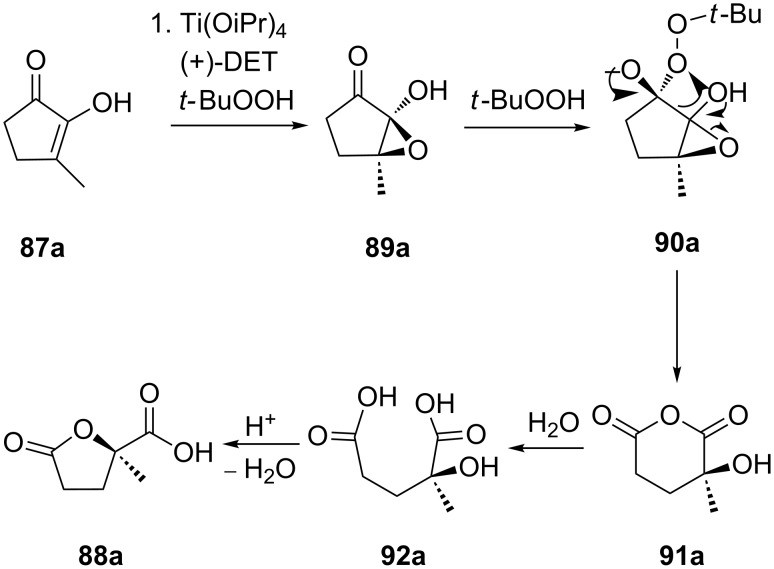
Mechanism of the asymmetric oxidation of cyclopentane-1,2-dione **87a** with the Ti(OiPr)_4_/(+)DET/*t*-BuOOH system.

The reaction starts with an asymmetric epoxidation of the substituted cyclopentane-1,2-dione **87a** to form epoxide **89a**. The second step involves the Baeyer–Villiger oxidation of epoxide **89a** to peroxide **90a** followed by the rearrangement into intermediate **91a**. The latter is hydrolyzed by H_2_O to form dicarboxylic acid **92a**, which is cyclized under the acidic conditions to γ-lactone acid **88a** (Scheme 29) [270].

**Scheme 30:**
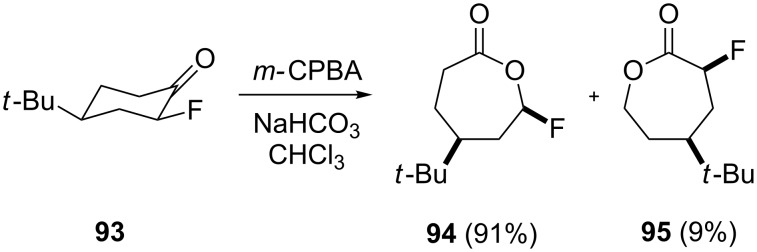
The oxidation of *cis*-4-*tert*-butyl-2-fluorocyclohexanone (**93**) with *m*-chloroperbenzoic acid.

In most cases, the Baeyer–Villiger oxidation is a stereospecific and regioselective process with retention of the configuration. The oxidation of *cis*-4-*tert*-butyl-2-fluorocyclohexanone (**93**) with *m*-chloroperbenzoic acid in the presence of NaHCO_3_ affords fluorolactones **94** and **95** in 91% and 9% yields, respectively (Scheme 30) [273].

However, in order to perform the asymmetric oxidation of 3-substituted cyclobutanones **96a**–**f** to the corresponding lactones **97a**–**f** (Table 7) [274], it is necessary to employ chiral Brønsted acids [274–277], organocatalysts [278–279] or enzymes [280–282] as the catalyst. The obtained asymmetric oxidation products can be used in the multistep synthesis of new biologically active compounds.

**Table 7:** Asymmetric oxidation of 3-substituted cyclobutanones **96a**–**f**.

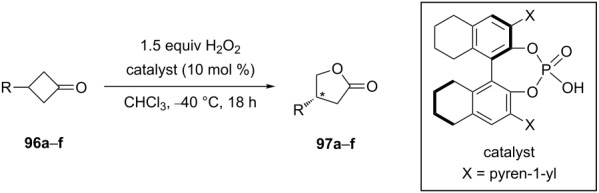			

Ketone	R	Yield, %	ee,% (conf.)

**96a**	C_6_H_5_	99	88 (*R*)
**96b**	4-MeC_6_H_4_	99	93 (*R*)
**96c**	4-FC_6_H_4_	99	84 (*R*)
**96d**	2-naphthyl	91	86 (*R*)
**96e**	C_6_H_5_CH_2_	99	58 (*S*)
**96f**	4-MeOC_6_H_4_CH_2_	99	57 (*S*)

**Scheme 31:**
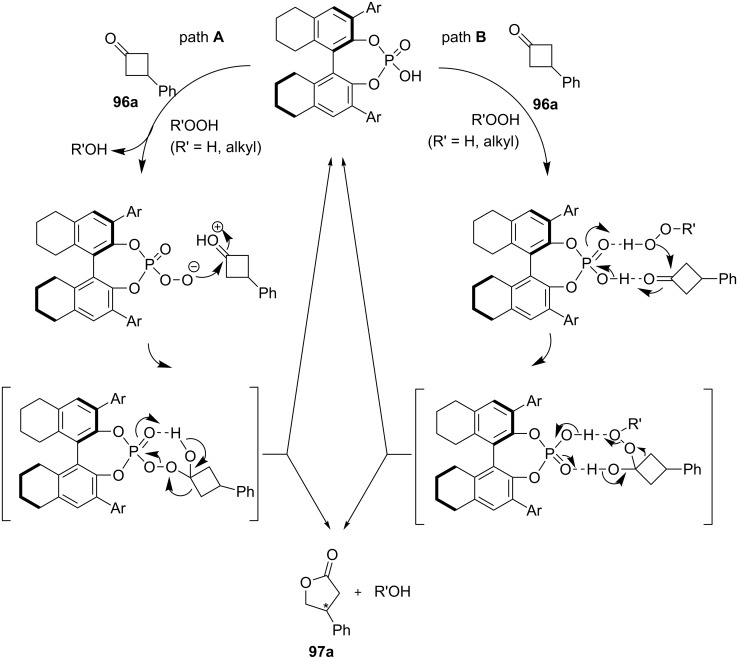
The mechanism of the asymmetric oxidation of 3-substituted cyclobutanone **96a** in the presence of chiral phosphoric acid.

Possible mechanisms for the asymmetric oxidation of 3-substituted cyclobutanone **96a** with H_2_O_2_ catalyzed by chiral phosphoric acid are presented in Scheme 31 [275].

**Scheme 32:**
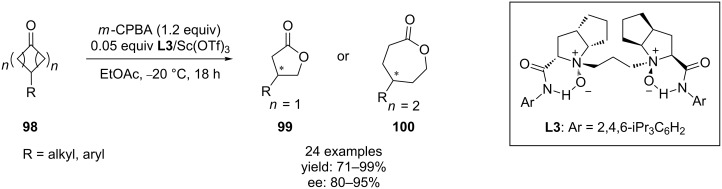
Enantioselective Baeyer–Villiger oxidation of cyclic ketones **98**.

A number of optically active ε- and γ-lactones **99**, **100** was prepared by the enantioselective Baeyer–Villiger oxidation of racemic cyclic ketones **98** in up to 99% yield and 95% ee using the chiral *N*,*N*′-dioxide–Sc(III) complex as catalyst (Scheme 32) [283].

In another work, a chiral *N*,*N*′-dioxide–Sc(III) complex promoted Baeyer–Villiger oxidation was applied as instrument for a kinetic resolution of racemic 2-substituted cyclopentanones with formation of the 6-substituted δ-lactones in up to 98% ee and >95% regioselectivity [284].

**Scheme 33:**
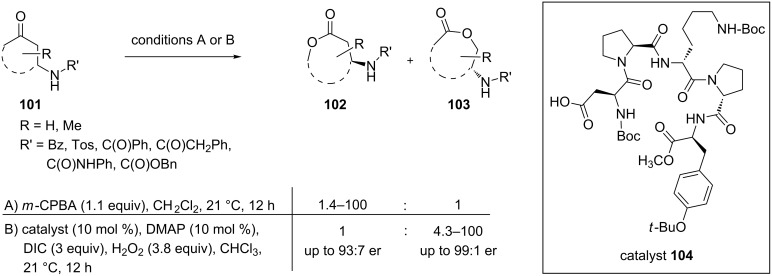
Regio- and enantioselective Baeyer–Villiger oxidation of cyclic ketones **101**.

A highly regio- and enantioselective Baeyer–Villiger oxidation of cyclic ketones **101** bearing amido, ureido, or sulfonamido functional groups to lactones **102** and **103** was carried out using the peptide-based catalyst **104**. Hydrogen-bonding interactions are responsible for both types of selectivity. Notably, a reversal of the typically seen selectivity was observed with the peptide catalyst (Scheme 33) [285].

**Versatility of the Baeyer–Villiger reaction with respect to starting reactants:** The Baeyer–Villiger reaction cannot only be performed with ketones but also with acetals and aldimines as the starting substrates. The oxidation of cycloalkanone acetals **105a**–**g** with performic acid generated in situ provides a new route to dicarboxylic acids **106a**–**g** and hydroxycarboxylic acids **107a**–**g** (Table 8) [286].

**Table 8:** Oxidation of cycloalkanone acetals **105a–g**.

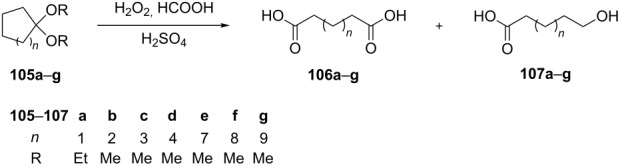				

Ketal	H_2_O_2_ (6% ethereal solution)		H_2_O_2_ (30% aqueous solution)	
	Yield of **106**, %	Yield of **107**, %	Yield of **106**, %	Yield of **107**, %

**105a**	14	51	11	61
**105b**	6	68	traces	61
**105c**	63	15	44	17
**105d**	77	16	57	21
**105e**	74	14	69	21
**105f**	62	22	72	15
**105g**	72	19	65	23

**Scheme 34:**
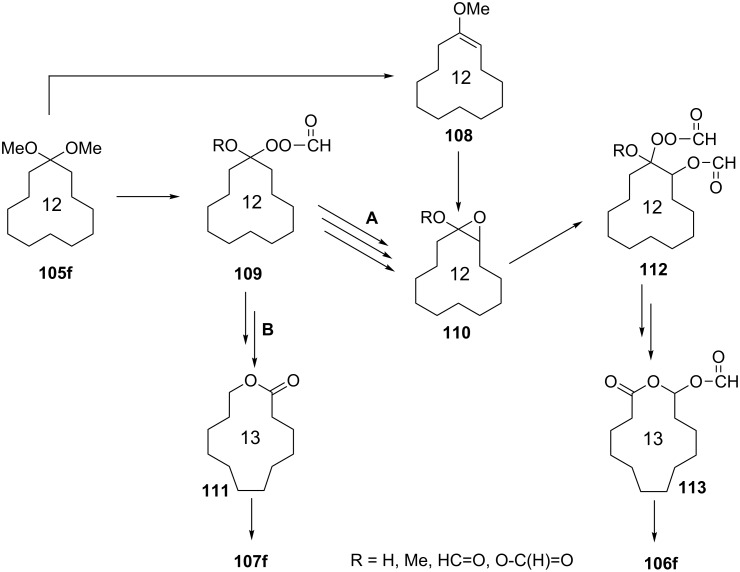
The proposed mechanism of the Baeyer–Villiger oxidation of acetal **105f**.

The proposed mechanism of the oxidation of acetal **105f** is shown in Scheme 34.

In the first step of the reaction, the elimination of methanol from **105f** and formation of **108** takes place. Probably perester **109** is formed alongside of **108**. After formation of **109**, the reaction proceeds by two different routes **A** and **B** (second stage). The first route **A** leads to formation of epoxide **110**, whereas the second route (**B**) proceeds through the Baeyer–Villiger reaction with formation of lactone **111** and subsequent acid hydrolysis to give **107f**. At the third stage (route **A**), ether **112** is formed from **110** and subsequently rearranged by a Baeyer–Villiger reaction into **113**, which is oxidized to form **106f**.

This method can be applied to the synthesis of dodecanedioic acid, which is used in anticorrosive composites, polyester and polyamide threads, and lubricants, for the synthesis of tridecanedioic acid, and as a component of perfume formulations.

**Scheme 35:**
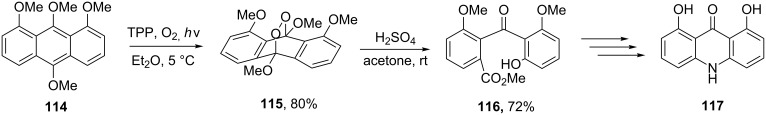
Synthesis of hydroxy-10*H*-acridin-9-one **117** from tetramethoxyanthracene **114**.

Scheme 35 presents the synthesis of hydroxy-10*H*-acridin-9-one **117** starting from tetramethoxyanthracene **114** through the formation of peroxide **115**, which rearranges through an acid-catalyzed Baeyer–Villiger-type rearrangement into **116**. Hydroxy-10*H*-acridin-9-ones **117** proved to be promising antipsoriatic agents [287].

The oxidation of aldimines **118a**–**f** with *m*-chloroperbenzoic acid in the presence of boron trifluoride etherate produces amides **119a**–**f** in good yields (Table 9). The products of this transformation are strongly dependent on the electronic properties of the aromatic substituents at the carbon atom of the aldimines [288]. In the case of electron-donating substituents on the aryl fragment (Ar), formamides **119a**–**c** are obtained as the result of imine oxidation and aryl migration. On the other hand, electron-withdrawing substituents on the aryl group (Ar) promote the formation of amides **119d**–**f** as result of hydride migration.

**Table 9:** Oxidation of aldimines **118a-f** to amides by *m-*CPBA-BF_3_·Et_2_O system.

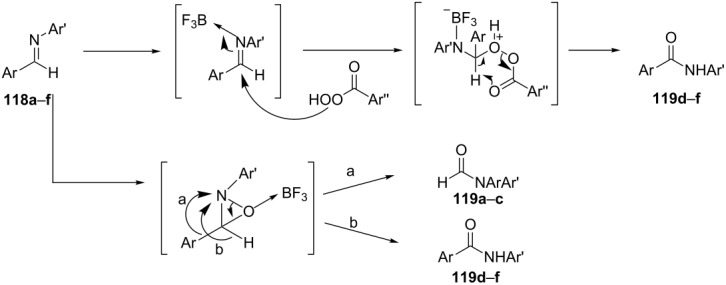			

Compound	Imine	Product	Yield, %

**118a**	C_6_H_5_CH=NC_6_H_5_	HCON(C_6_H_5_)_2_	82
**118b**	*p*-Me-C_6_H_4_CH=NC_6_H_5_	HCONC_6_H_5_ *p*-Me-C_6_H_4_	90
**118c**	*p*-MeO-C_6_H_4_CH=NC_6_H_5_	HCONC_6_H_5_ *p*-MeO-C_6_H_4_	91
**118d**	*p*-NO_2_-C_6_H_4_CH=NC_6_H_5_	*p*-NO_2_-C_6_H_4_CONHC_6_H_5_	71
**118e**	*p*-NC-C_6_H_4_CH=NC_6_H_5_	*p*-NC-C_6_H_4_CONHC_6_H_5_	79
**118f**	*p*-F_3_C-C_6_H_4_CH=NC_6_H_5_	*p*-F_3_C-C_6_H_4_CONHC_6_H_5_	75

**Scheme 36:**
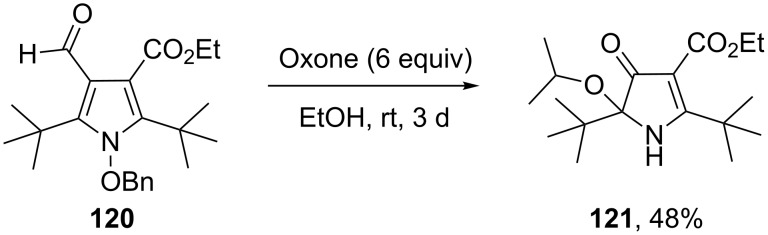
The Baeyer–Villiger oxidation of the fully substituted pyrrole **120**.

The sterically hindered and fully substituted pyrrole **120** underwent a Baeyer–Villiger reaction to yield a 4,5-dihydro-1*H*-ketopyrrole **121** (Scheme 36) [289].

#### 1.2 Criegee rearrangement

**Scheme 37:**
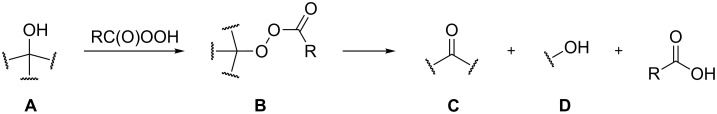
The Criegee rearrangement.

The Criegee rearrangement involves the transformation of a peroxide, mainly peroxyesters **B**, into carbonates, esters, or ketones **C** and alcohols **D** through an oxygen insertion or consecutive oxygen insertions. The peroxyester **B** is initially prepared from a tertiary alcohol **A** and a peracid. In addition, the peroxy ester can also be prepared via the reaction of a ketone and a peracid (i.e., through a Baeyer–Villiger oxidation); the additional product of peracid to ketone is often referred to as the Criegee intermediate. From this point of view, the Baeyer–Villiger oxidation is a subset of the Criegee rearrangement (Scheme 37) [290].

**Scheme 38:**
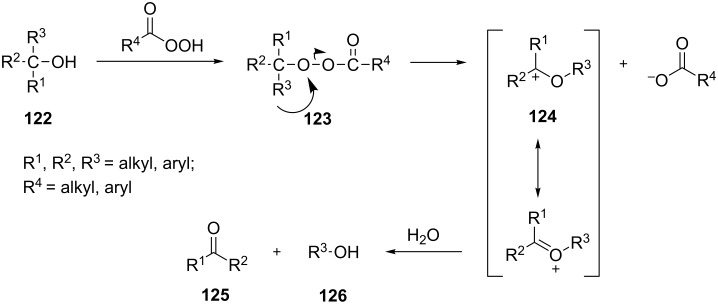
The mechanism of the Criegee reaction of a peracid with a tertiary alcohol **122**.

As mentioned above the Criegee reaction and the Baeyer–Villiger oxidation are related processes and both reactions involve the formation of the Criegee intermediate. The distinguishing feature of the Criegee rearrangement is that the Criegee intermediate rearranges into a carbocation. The mechanism of the Criegee reaction is presented in Scheme 38.

Initially the reaction of the peracid with the tertiary alcohol **122** produces perester (Criegee intermediate) **123**. One alkyl substituent migrates from the carbon atom to the adjacent oxygen atom and replaces the carboxylic acid moiety to form carbocation **124**. Then, the addition of water to carbocation **124** affords ketone **125** and alcohol **126**. *p*-Nitroperbenzoic acid is usually used to oxidize tertiary alcohols because the anion of this acid is a good leaving group.

**Scheme 39:**
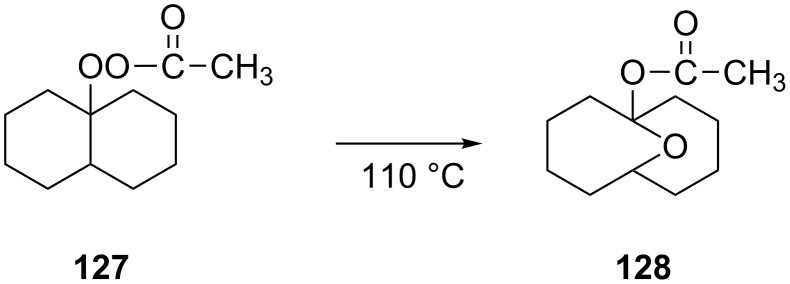
Criegee rearrangement of decaline ethylperoxoate **127** into ketal **128**.

The Criegee rearrangement was discovered in 1944 in the reaction of decaline ethylperoxoate **127** that rearranged into isomeric ester ketal **128** (Scheme 39) [291].

**Scheme 40:**
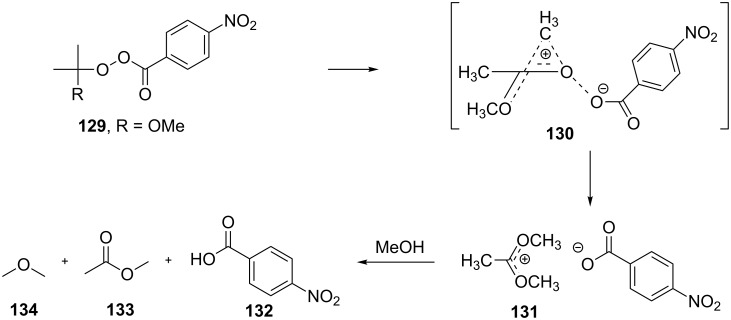
The ionic cleavage of 2-methoxy-2-propyl perester **129**.

The mechanism of the Criegee rearrangement was studied using 2-alkoxy-2-propyl per-4-nitrobenzoates [292]. It was shown that the ionic cleavage of 2-methoxy-2-propyl perester **129** to *p*-nitrobenzoic acid (**132**), methyl acetate (**133**) and dimethyl ether (**134**) occurred through transition state **130** with generation of dimethoxycarbonium ion **131** (Scheme 40).

Investigations using aromatic peroxy esters **129** demonstrated that the migratory ability of the migrating group R decreases in the series *t*-Bu > C_6_H_5_ > iPr > OEt > OMe > Et > Me [293–294].

**Scheme 41:**
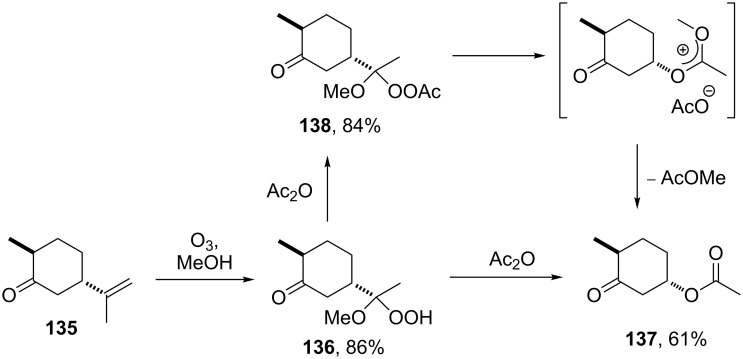
The Criegee rearrangement of α-methoxy hydroperoxide **136**.

The Criegee rearrangement of α-methoxy hydroperoxide **136** obtained from (+)-*trans*-dihydrocarvone **135** produces *trans*-5-acetoxy-2-methylcyclohexanone **137** and intermediate peroxyacetate **138** (Scheme 41) [295].

**Scheme 42:**
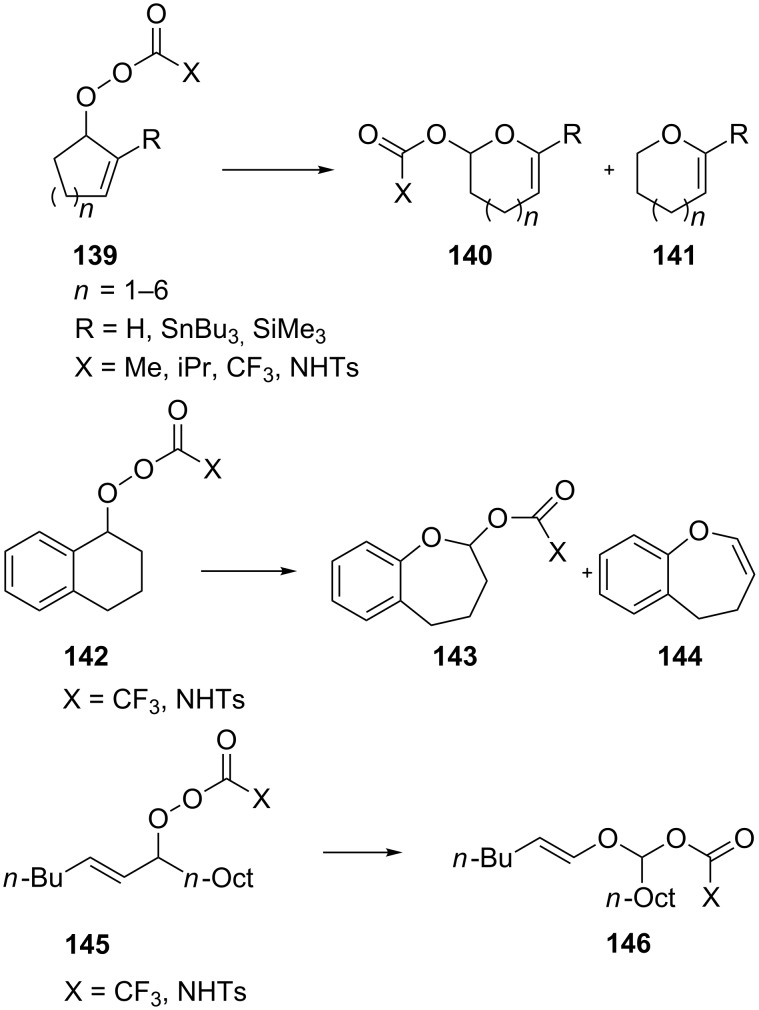
Synthesis of enol esters and acetals via the Criegee rearrangement.

Later on, the Criegee rearrangement was extended [296] to peroxides **139**, **142**, and **145** which made it possible to selectively synthesize both cyclic **140**, **141**, **144** and acyclic enol esters **146** and acetal **143** (Scheme 42).

The Criegee rearrangement of 1-hydroperoxy-2-oxabicycloalkanes **147a**–**d** in formic or acetic acid containing catalytic amounts of sulfuric acid affords ω-alkoxy-(ω-3)-hydroxyalkanoic acid lactones **148a**–**d** and **149a**–**d** (Table 10) [297].

**Table 10:** Synthesis of ω-alkoxy-(ω-3)-hydroxyalkanoic acid lactones **148a**–**d** and **149a**–**d** from 1-hydroperoxy-2-oxabicycloalkanones **147a**–**d**.

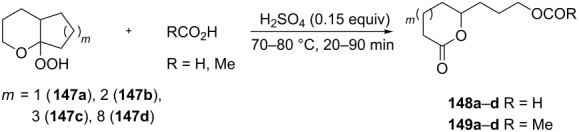				

Substrate	RCOOH	Time (min)	Lactone	Yield, %

**147a**	HCOOH	20	**148a**	64
**147a**	AcOH	20	**149a**	65
**147b**	HCOOH	20	**148b**	68
**147b**	AcOH	20	**149b**	70
**147c**	HCOOH	30	**148c**	57
**147c**	AcOH	30	**149c**	65
**147d**	HCOOH	90	**148d**	68
**147d**	AcOH	90	**149d**	53

**Scheme 43:**
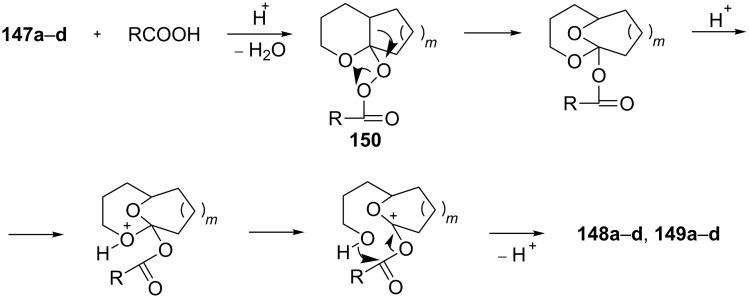
Proposed mechanism of the transformation of 1-hydroperoxy-2-oxabicycloalkanones **147a**–**d**.

The transformation of 1-hydroperoxy-2-oxabicycloalkanones **147a**–**d** into ω-alkoxy-(ω-3)-hydroxyalkanoic acid lactones **148a**–**d** and **149a**–**d** is proposed to occur through intermediate peroxy ester **150** (Scheme 43).

**Scheme 44:**
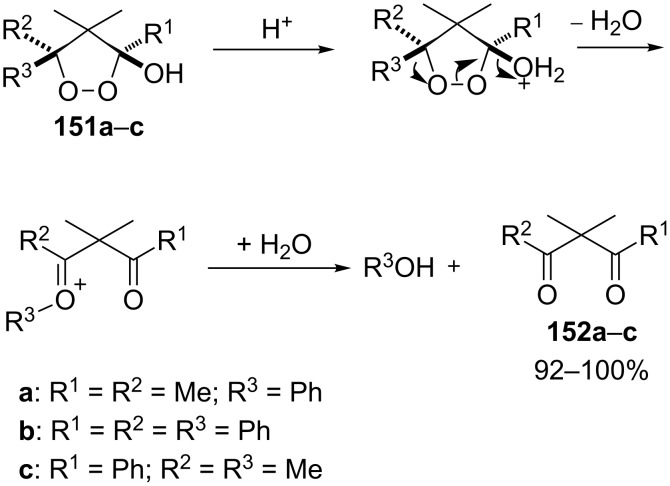
Transformation of 3-hydroxy-1,2-dioxolanes **151** into diketone derivatives **152**.

1,2-Dioxolanes and related cyclic systems have attracted considerable attention from synthetic chemists as they may be used for the preparation of biologically active compounds. Under acidic conditions, 3-hydroxy-1,2-dioxolanes **151** are rearranged similarly to the Criegee mechanism into diketone derivatives **152** (Scheme 44) [298].

**Scheme 45:**
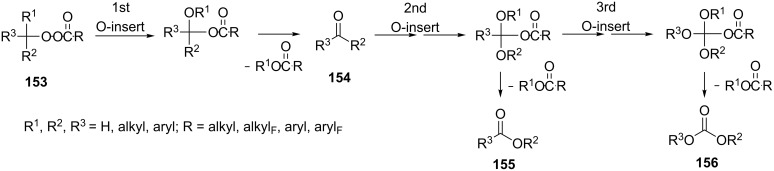
Criegee rearrangement of peroxide **153** with the mono-, di-, and tri-O-insertion.

Unlike the Baeyer–Villiger rearrangement, in which only mono-O-insertion can take place, the Criegee rearrangement of peroxide **153** in an acidic medium and under solvent-free conditions does not have such limitations. Thus, the latter reaction can proceed sequentially through the mono-, di-, and tri-O-insertion steps with formation of ketone **154**, ester **155** and carbonate ester **156** (Scheme 45) [299–300].

**Scheme 46:**
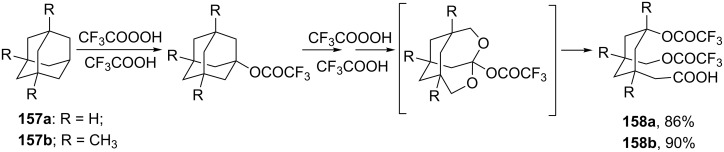
The sequential Criegee rearrangements of adamantanes **157a**,**b**.

The selective double Criegee rearrangement next to a tertiary carbon was shown in the oxidative fragmentation at the bridgehead position of adamantanes **157a**,**b**. The reaction employed the trifluoroperacetic acid (TFPAA)/trifluoroacetic acid (TFAA) system and afforded compounds **158a**,**b** in high yields (Scheme 46) [300].

**Scheme 47:**
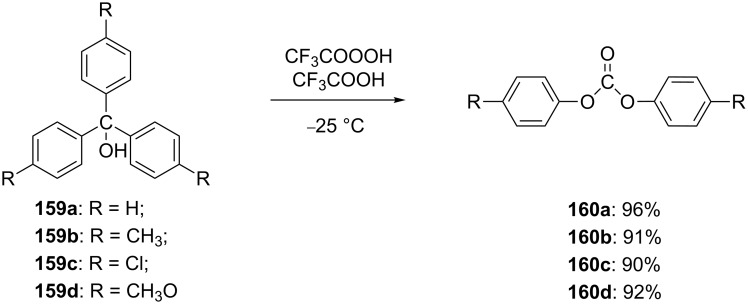
Synthesis of diaryl carbonates **160a**–**d** from triarylmethanols **159a**–**d** through successive oxygen insertion.

This method for the insertion of an oxygen atom was applied to the oxidation of triarylmethanols **159a**–**d** [299]. The successive insertion of oxygen atoms gave rise to diaryl carbonates **160a**–**d** in good yields (Scheme 47).

**Scheme 48:**
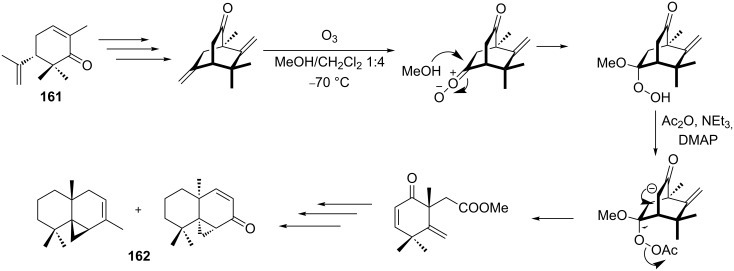
The synthesis of sesquiterpenes **162** from ketone **161** with a Criegee rearrangement as one key step.

In the last years, new enantiospecific approaches for the synthesis of sesquiterpenes **162** from ketone **161** were developed [301–307]. In these methods, the Criegee rearrangement represents one key step and one example is presented in Scheme 48 [303].

**Scheme 49:**
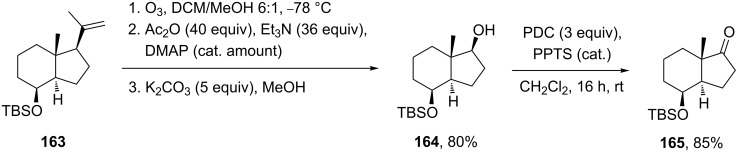
Synthesis of *trans*-hydrindan derivatives **164**, **165**.

A method for the large-scale synthesis of a *trans*-hydrindan derivatives **164**, **165** related to vitamin D, based on the Criegee rearrangement of alkene **163** was realized (Scheme 49) [308].

Carbonyl oxides (Criegee intermediates) are one of the most important compounds in tropospheric chemistry [309]. Direct investigations of formaldehyde oxide (CH_2_OO) or acetaldehyde oxide (CH_3_CHOO) reactions with water vapor, SO_2_, NO_2_ were carried out [310–312].

#### 1.3 Hock rearrangement

**Scheme 50:**
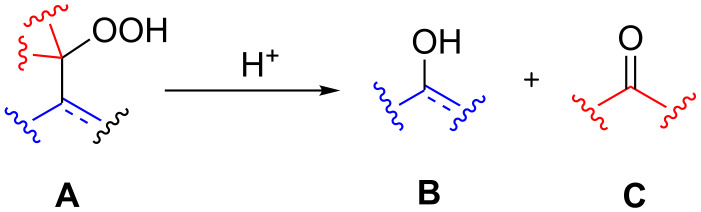
The Hock rearrangement.

The Hock rearrangement is a protic or Lewis acid-promoted rearrangement of hydroperoxides **A** resulting in a C–C bond cleavage to form alcohol **B** and carbonyl compound **C** (Scheme 50) [313].

**Scheme 51:**
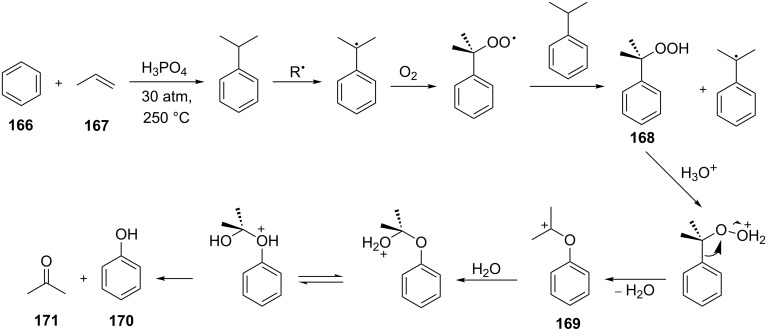
The general scheme of the cumene process.

The Hock rearrangement is a key step in the cumene process, which is used for the industrial production of phenol (**170**) and acetone (**171**) from benzene (**166**) and propylene (**167**) in the presence of air and radical initiators. The cumene process was described by Udris and Sergeev in 1947 [314–315] and independently by Hock in 1944 [316–317]. The general scheme of the cumene process, involving the formation of cumene hydroperoxide is shown in Scheme 51.

The cumene process involves the acid-catalyzed rearrangement of cumene hydroperoxide (**168**) as a key step. The reaction starts with the protonation of the terminal oxygen atom of cumene hydroperoxide (**168**) followed by the migration of the phenyl group from the benzylic carbon atom to the peroxide oxygen atom and the elimination of a water molecule to form carbocation **169**. The carbocation **169** is attacked by a water molecule, a proton is transferred to the oxygen atom attached to the phenyl group, and finally the cleavage of the adduct yields phenol (**170**) and acetone (**171**).

**Scheme 52:**
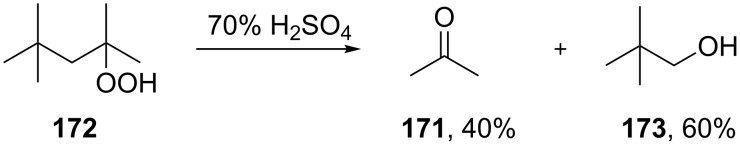
The Hock rearrangement of aliphatic hydroperoxides.

The Hock rearrangement of aliphatic hydroperoxides proceeds quite readily in concentrated H_2_SO_4_ [318] or superacids [319] (Scheme 52). This is associated with higher resistance of these compounds toward acid-catalyzed rearrangements compared with benzylic or allylic hydroperoxides. For example, aliphatic hydroperoxides are not cleaved in 5–50% aqueous H_2_SO_4_ but on the contrary, these compounds are produced under these conditions. More efficient catalysts are the compounds Sn(OTf)_2_ and La(OTf)_3_ which can be used for the transformation of 2-hydroperoxy-2,4,4-trimethylpentane (**172**) into neopentyl alcohol (**173**) and acetone (**171**). The Sn(OTf)_2_ and La(OTf)_3_-catalyzed reaction afforded neopentyl alcohol (**173**) in 62 and 70% yield, respectively [320].

The hydrogen peroxide promoted ring expansion for the synthesis of oxabicycles **176a**–**c** was described for the first time in 1985 [321]. The reaction involved the solvolysis of homoallylic brosylates **174a**–**c** or spiro cyclopropyl carbinols **175a**–**c** in the THF/H_2_O_2_ system, resulting in the increase in the ring size by two atoms and the formation of hydroperoxy oxabicyclo derivatives **176a**–**c** (Table 11).

**Table 11:** Solvolysis of brosylates **174a–c** and spiro cyclopropyl carbinols **175a–c** in the THF/H_2_O_2_ system.

Substrate	Product (yield, %)	Substrate	Product (yield, %)

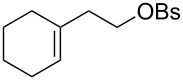 **174a**	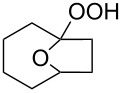 **176a** (78%)	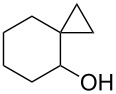 **175a**	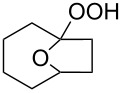 **176a** (90%)
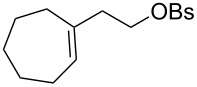 **174b**	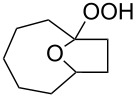 **176b** (73%)	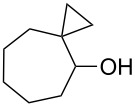 **175b**	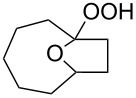 **176b** (91%)
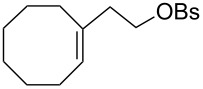 **174c**	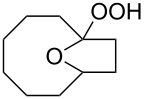 **176c** (84%)	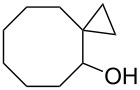 **175c**	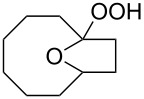 **176c** (91%)

**Scheme 53:**
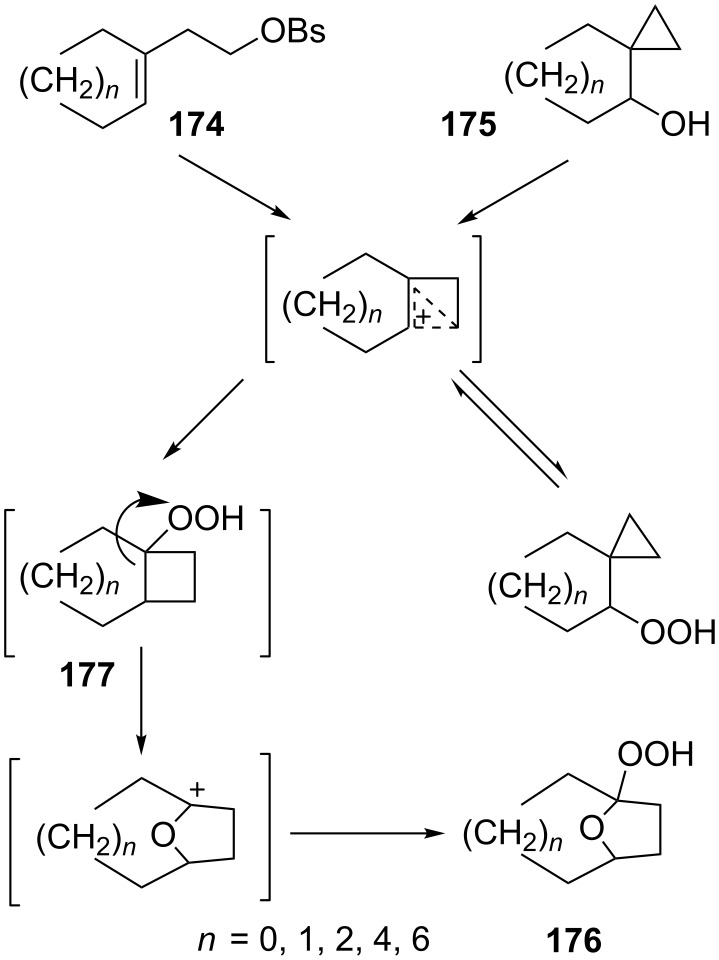
The mechanism of solvolysis of brosylates **174a–c** and spiro cyclopropyl carbinols **175a–c** in THF/H_2_O_2_.

The mechanism of the solvolysis of **174** or **175** in the THF/H_2_O_2_ system involves the formation of solvolytically generated cyclobutyl hydroperoxides **177** followed by the rearrangement of the latter into oxa-bridged, hydroperoxyhemiketals **176** (Scheme 53).

The fragmentation of hydroperoxy acetals **178a**–**e** in the presence of Ca(OCl)_2_ or *t*-BuOCl as the catalysts in CH_3_CN generating esters **179a**–**e** proceeds through the Hock-like rearrangement mechanism (Table 12) [322].

**Table 12:** Fragmentation of hydroperoxy acetals **178a**–**e** catalyzed by Ca(OCl)_2_ or *t*-BuOCl.

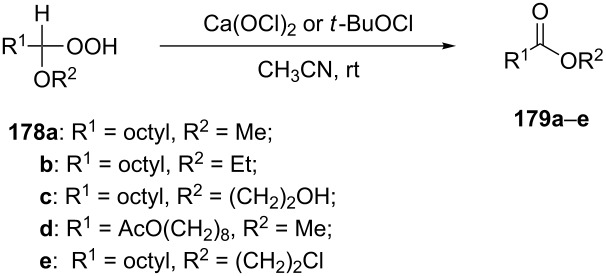							

Substrate	Product	Ca(OCl)_2_ (equiv)	Time (min)	Yield, %	*t*-BuOCl (equiv)	Time (min)	Yield, %

**178a**	**179a**	1.3	10	75	0.25	15	78
**178b**	**179b**	1.3	10	86	1.2	10	85
**178c**	**179c**	1.3	10	83	1.2	10	84
**178d**	**179d**	1.3	10	85	0.25	15	85
**178e**	**179e**	1.3	10	82	1.2	10	84

**Scheme 54:**
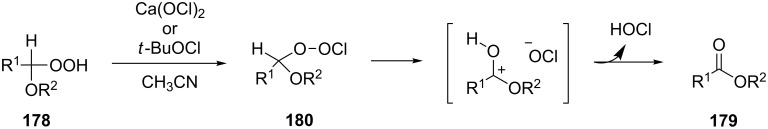
The fragmentation mechanism of hydroperoxy acetals **178** to esters **179**.

The fragmentation of hydroperoxy acetals **178** to esters **179** involves the formation and heterolytic fragmentation of intermediate secondary chloroperoxides **180**. The possible mechanism of the process is presented in Scheme 54.

**Scheme 55:**
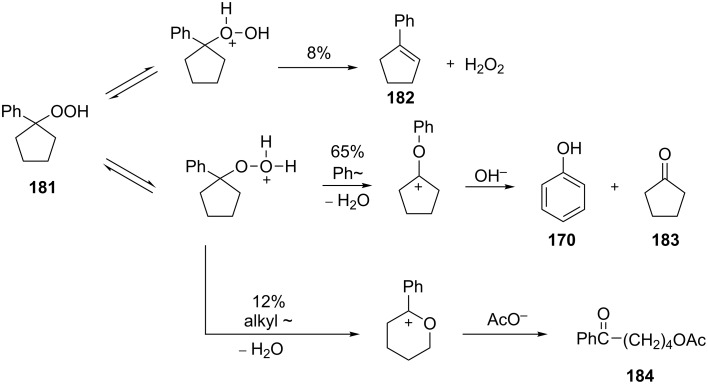
The acid-catalyzed rearrangement of phenylcyclopentyl hydroperoxide **181**.

The acid-catalyzed rearrangement of phenylcyclopentyl hydroperoxide **181**, involving the Hock reaction, is accompanied by the formation of a series of products: 1-phenylcyclopentene (**182**), phenol (**170**), cyclopentanone (**183**), and 5-acetoxyvalerophenone (**184**) (Scheme 55) [323].

**Scheme 56:**
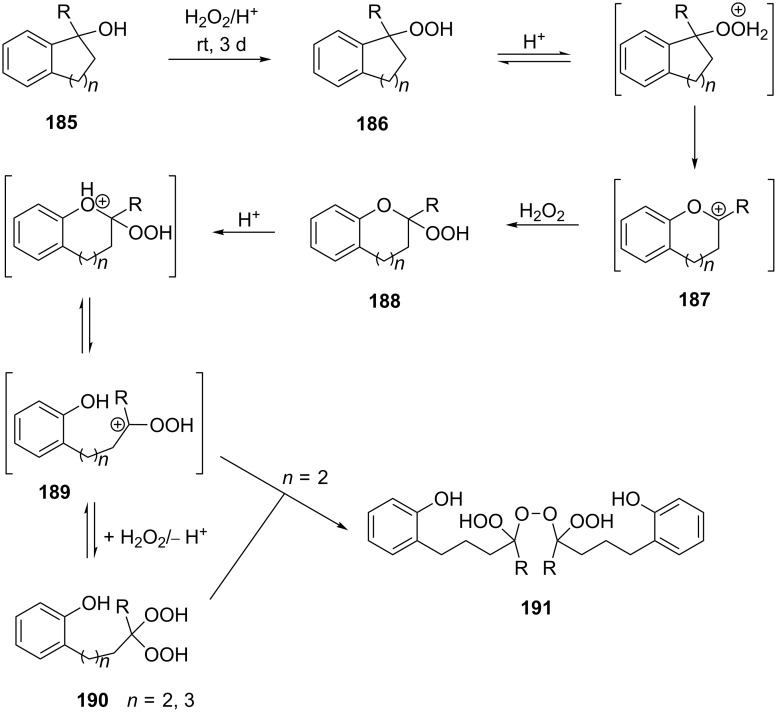
The peroxidation of tertiary alcohols in the presence of a catalytic amount of acid.

An attempt was made [324] to synthesize hydroperoxides through the peroxidation of tertiary alcohols in the presence of a catalytic amount of acid. The treatment of **185** with H_2_O_2_ in the presence of a catalytic amount of H_2_SO_4_ for 72 hours did not lead to the formation of products via the Hock rearrangement of hydroperoxides, bicyclic hydroperoxides and *о*-hydroxyphenyl alkyl ketones. Instead, cyclic 2-methylchroman-2-yl hydroperoxide **188**, geminal bishydroperoxides **190**, and condensation products of peroxides such as **191** were isolated (Scheme 56).

The reaction mechanism presumably involves the following steps: the replacement of the hydroxy group by hydrogen peroxide to form tertiary hydroperoxides **186**, the acid-catalyzed rearrangement of compounds **186** into cyclic phenoxycarbenium ions **187**, and the addition of the second hydrogen peroxide molecule to **187** resulting in the formation of cyclic phenoxy hydroperoxide **188**. The latter was isolated as the major product in the case of the six-membered ring (*n* = 1). In the case of the seven-membered ring (*n* = 2), geminal dihydroperoxide **190** and bridged bis(hydroxy)dialkyl peroxide **191** were obtained instead of **188**. In case of the eight-membered ring (*n* = 3) an exclusive transformation into geminal dihydroperoxide **190** was observed (Table 13).

**Table 13:** Yields of products **188**, **190**, and **191**.

Entry	R	*n*	**185**	(**185**:H_2_O_2_)	Yield, %		

					**188**	**190**	**191**

1	Me	1	**a**	(1:10)	**a** (65)		
2	Me	2	**b**	(1:10)		**b** (30)	**b** (48)
3	Et	2	**b**	(1:10)		**c** (26)	**c** (36)
4	Me	3	**c**	(1:10)		**d** (12)	

**Scheme 57:**
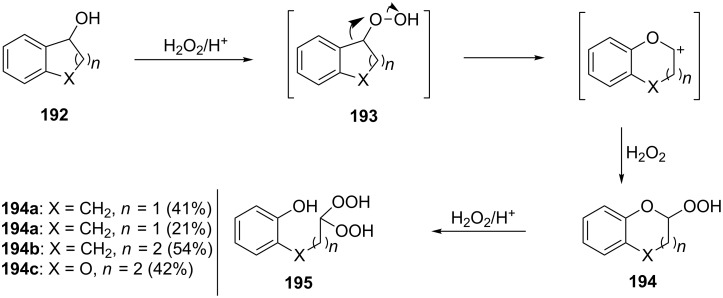
The acid-catalyzed reaction of bicyclic secondary alcohols **192** with hydrogen peroxide.

The formation of geminal dihydroperoxides **195** was also observed in the acid-catalyzed reaction of bicyclic secondary alcohols **192** with hydrogen peroxide. This reaction starts with the formation of bicyclic hydroperoxides **193** followed by the acid-catalyzed rearrangement with intermediate formation of peroxy hemiacetal **194**. The latter is finally transformed into primary geminal bishydroperoxides **195** (Scheme 57) [325].

**Scheme 58:**
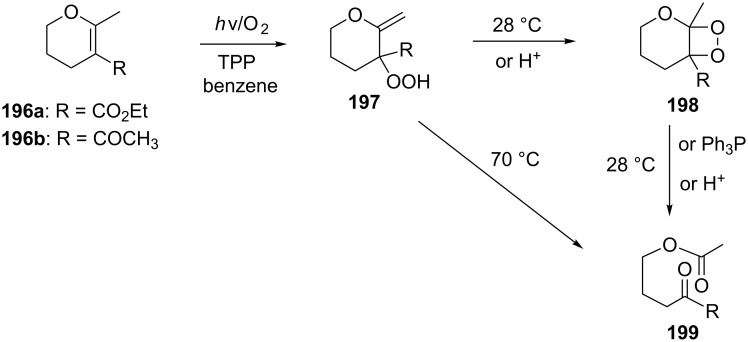
The photooxidation of 5,6-disubstituted 3,4-dihydro-2*H*-pyrans **196**.

The photooxidation of 5,6-disubstituted 3,4-dihydro-2*H*-pyrans **196** generates the stable hydroperoxide **197** as the major product, which rearranges into dioxetane **198** at 28 °C in CCl_4_ within 13 h. Compounds **198** can be further transformed into keto esters **199** by treatment for 24 h with triphenylphosphine in CCl_4_ or concentrated HCl in CCl_4_. When compound **197** is heated at 70 °C its rearrangement into **199** occurs very rapidly and dioxetane **198** was not detected (Scheme 58) [326–327].

**Scheme 59:**
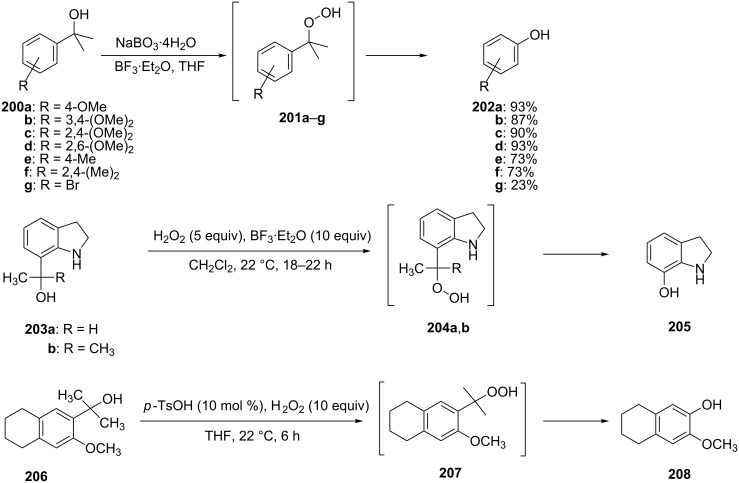
The oxidation of tertiary alcohols **200a**–**g**, **203a**,**b**, and **206**.

The oxidation of tertiary alcohols **200a**–**g**, **203a**,**b**, and **206**, involving the rearrangement of hydroperoxides **201a**–**g**, **204a**,**b**, and **207**, occurs in good yields in the presence of such systems as NaBO_3_·4H_2_O/BF_3_·Et_2_O [328], H_2_O_2_/BF_3_·Et_2_O, and H_2_O_2_/*p*-TsOH [329] (Scheme 59). The Hock rearrangement can be used to prepare alcohols **202a**–**g**, **205**, and **208** containing electron-donating substituents.

**Scheme 60:**
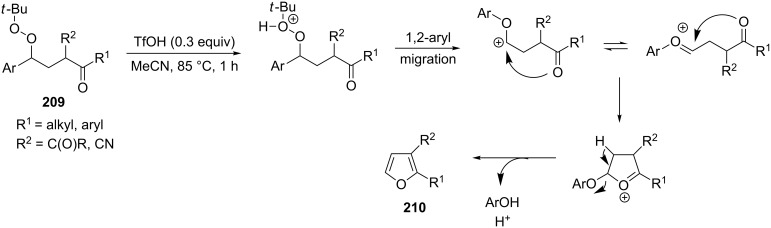
Transformation of functional peroxide **209** leading to 2,3-disubstitued furans **210** in one step.

The intramolecular capture of the cationic intermediate derived from the Hock rearrangement of peroxyketone **209** provides a direct and efficient one-step synthesis of 2,3-disubstituted furans **210** (Scheme 60) [330].

**Scheme 61:**
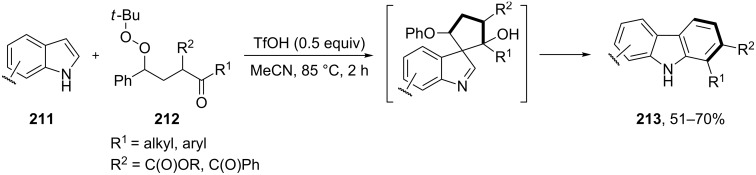
The synthesis of carbazoles **213** via peroxide rearrangement.

The benzannulation of indoles **211** can be performed with γ-carbonyl *tert*-butyl peroxides **212** catalyzed by trifluoromethanesulfonic acid to give carbazoles **213**. The key step of this approach is based on the acid-catalyzed rearrangement of *tert*-butyl peroxides (Scheme 61) [331].

**Scheme 62:**
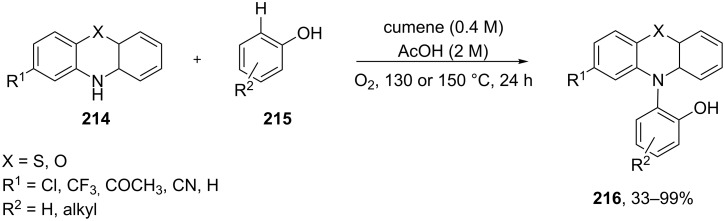
The construction of C–N bonds using the Hock rearrangement.

The direct dehydrogenative construction of C–N bonds between unprotected phenols **215** and a series of 10*H*-phenoxazines and 10*H*-phenothiazines **214** with formation of **216** was carried out using a Hock-like activation with O_2_ followed by amine oxidation (Scheme 62) [332].

**Scheme 63:**
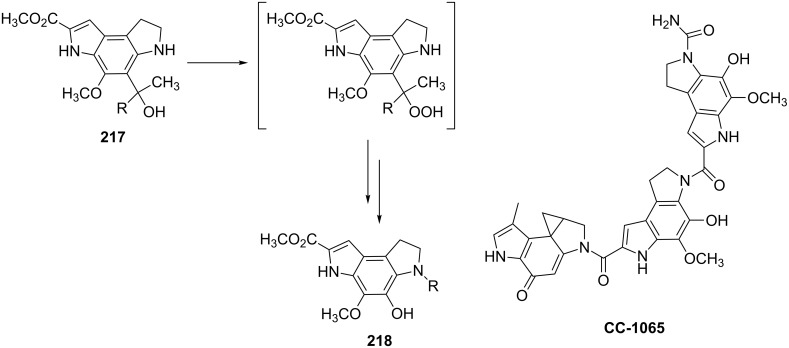
The synthesis of moiety **218** from **217** which is a structural motif in the antitumor–antibiotic of **CC-1065**.

The Hock rearrangement plays an important role not only in fine organic synthesis but also in biological processes. Scheme 63 shows the proposed mechanism for the biosynthetic conversion of **217** to **218**, which is an important component of the structural skeleton of the antitumor–antibiotic **CC-1065** [333].

**Scheme 64:**
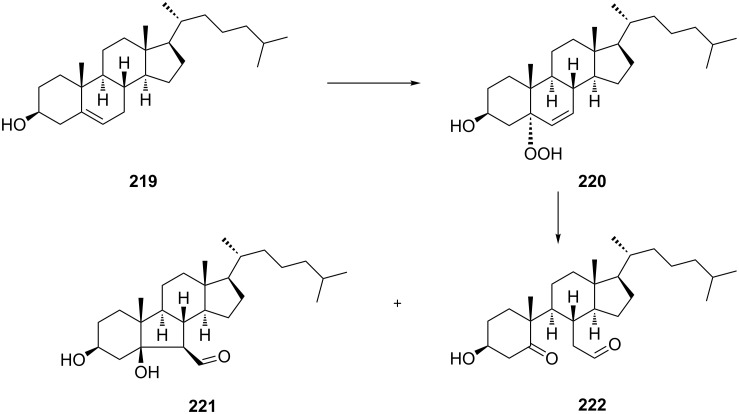
The in vivo oxidation steps of cholesterol (**219**) by singlet oxygen.

The synthetic model of the in vivo oxidation of cholesterol (**219**) by singlet oxygen produces cholesterol-5α-OOH **220**, which is subjected to a Hock reaction to form the aldolization product **221** and keto aldehyde (atheronal A, **222**) (Scheme 64) [67].

**Scheme 65:**
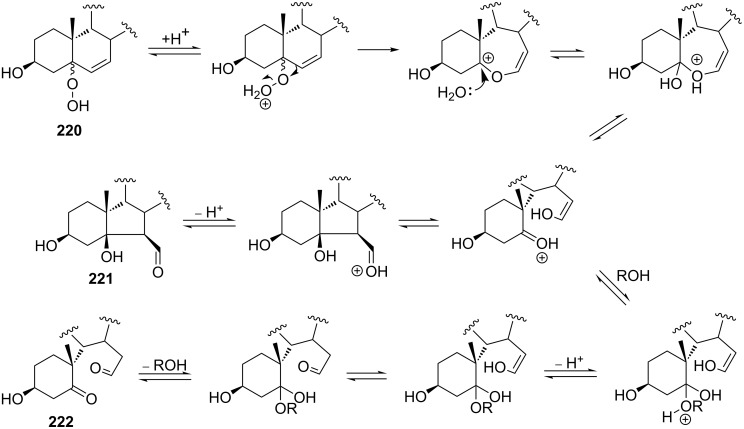
The proposed mechanism of the rearrangement of cholesterol-5α-OOH **220**.

Keto aldehyde (atheronal A, **222**) exhibits proatherogenic activity and plays a causal role in the development of cardiovascular diseases [66]. The proposed mechanism of the rearrangement of cholesterol-5α-OOH **220** is presented in Scheme 65.

Therefore, the acid-catalyzed Hock rearrangement of hydroperoxide **220** is a key step in the oxidation of cholesterol (**219**).

**Scheme 66:**
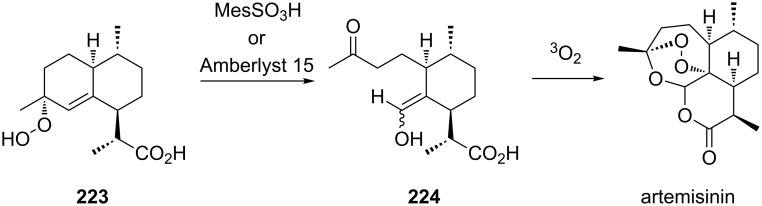
Photochemical route to artemisinin via Hock rearrangement of **223**.

In a photochemical route developed for the synthesis of artemisinin the Hock rearrangement of hydroperoxide **223** selectively affords enol **224**. This reactive intermediate **224** is then ﬁnally oxidized into artemisinin (Scheme 66) [334].

#### 1.4 Kornblum−DeLaMare rearrangement

**Scheme 67:**
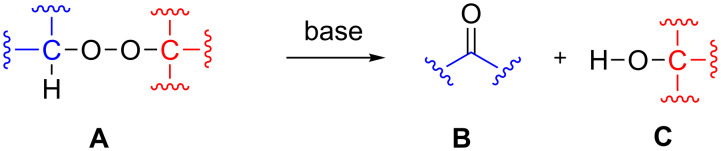
The Kornblum–DeLaMare rearrangement.

The Kornblum−DeLaMare rearrangement (KDLM) is a rearrangement of organic peroxides **A** containing a primary or secondary carbon atom into ketones **B** and alcohols **C** mainly under base-catalyzed reaction conditions (Scheme 67) [335].

**Scheme 68:**
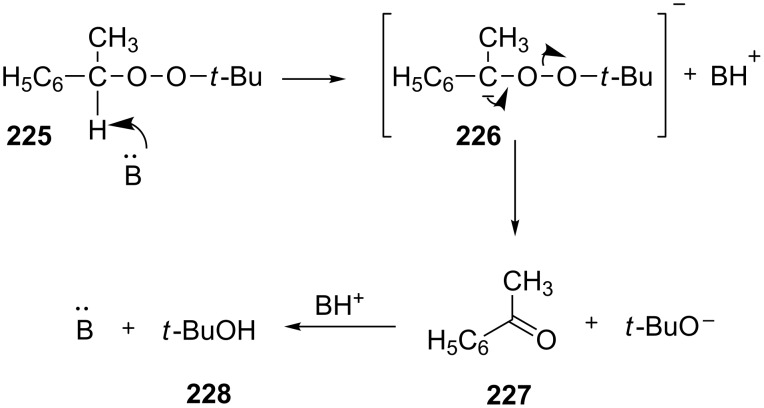
Kornblum–DeLaMare transformation of 1-phenylethyl *tert*-butyl peroxide (**225**).

In 1951, Kornblum and DeLaMare observed that the treatment of 1-phenylethyl *tert*-butyl peroxide (**225**) with KOH, NaOEt, or pyridine resulted in the decomposition of **225** to give acetophenone (**227**) and *tert*-butanol (**228**). A three-step mechanism for this reaction was proposed (Scheme 68) [336–337].

The reaction commences with a base-mediated α-proton abstraction from **225** to form carbanion **226** and the latter decomposes to yield the *tert*-butoxide anion and acetophenone (**227**). These steps occur presumably in a concerted manner. Finally, the protonation of the *tert*-butoxide anion results in the formation of *tert*-butanol (**228**). As alternative bases Et_3_N [338–339], phosphorus ylides [340] and LiOH [341–342] can be used and the Kornblum–DeLaMare rearrangement proceeds also on SiO_2_ [343].

The Kornblum–DeLaMare rearrangement is a convenient tool in organic chemistry for the conversion of monocyclic endoperoxides. These compounds are discussed in this review in the order of increasing ring size and the number of the starting substrates.

**Scheme 69:**
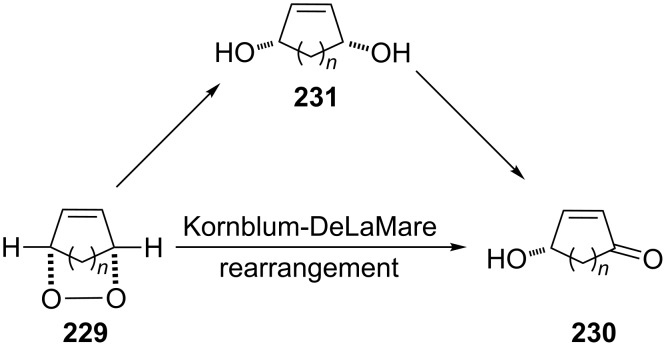
The synthesis 4-hydroxyenones **230** from peroxide **229**.

The treatment of unsubstituted bicyclic endoperoxides **229** by bases affords 4-hydroxyenones **230** [344] which are useful precursors in asymmetric organic syntheses. Alternative synthetic methods towards this class of compounds normally require a metal-catalyzed or biocatalyzed oxidation of diols **231** in an additional reaction step [345] (Scheme 69).

**Scheme 70:**
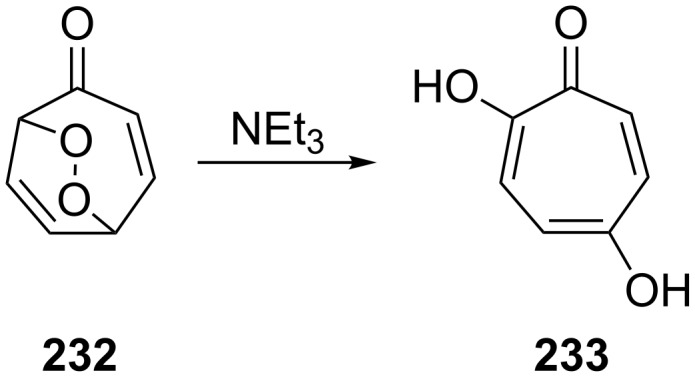
The Kornblum–DeLaMare rearrangement of peroxide **232**.

The treatment of endoperoxide **232** with triethylamine in ethanol at room temperature results in the O–O-bond cleavage to form 5-hydroxytropolone (**233**) (Scheme 70) [346].

**Scheme 71:**
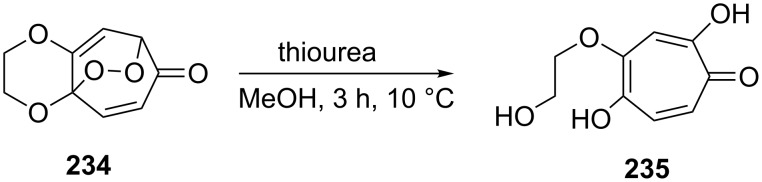
The reduction of peroxide **234**.

It is interesting to note, that a reduction of the bicyclic endoperoxide **234** with thiourea in methanol at 10 °C produces similar to KDLM product tropolone **235** in 94% yield (Scheme 71) [347].

**Scheme 72:**
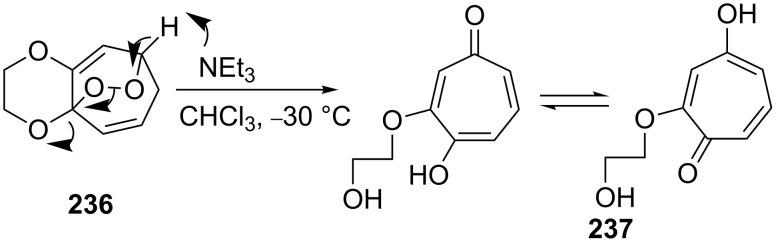
The Kornblum–DeLaMare rearrangement of endoperoxide **236**.

The Kornblum–DeLaMare reaction of the endoperoxide **236** with triethylamine in chloroform at −30 °C affords tropolone **237** in 97% yield (Scheme 72) [347].

Tropolones exhibit a broad spectrum of biological activities, including antibacterial, antiviral, antifungal, anti-allergic, anti-oxidant, and anti-inflammatory [348–349].

**Scheme 73:**
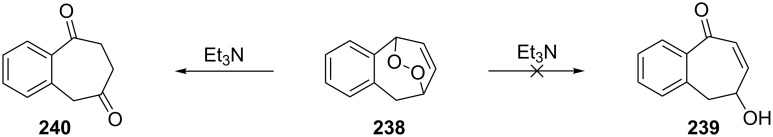
The rearrangement of peroxide **238** under Kornblum–DeLaMare conditions.

The treatment of endoperoxide **238** with Et_3_N gave 1,4-diketone **240** in quantitative yield instead of expected hydroxy ketone **239** (Scheme 73) [350–352].

**Scheme 74:**
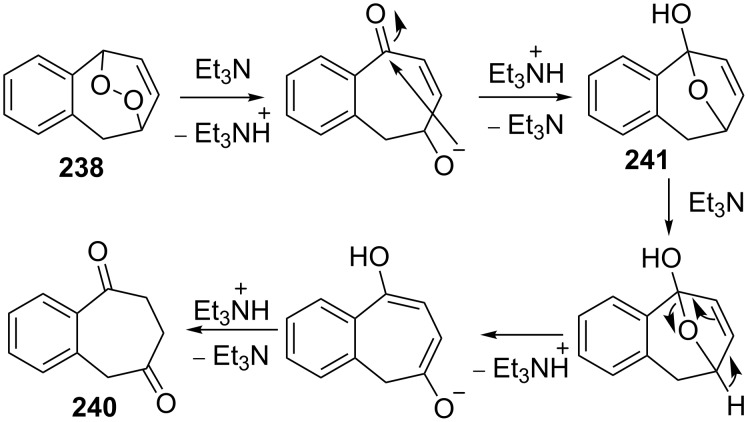
The proposed mechanism of rearrangement of peroxide **238**.

The endoperoxide **238** is presumably converted into hemiketal **241**, which is rearranged in several steps into diketone **240** (Scheme 74) [351].

**Scheme 75:**
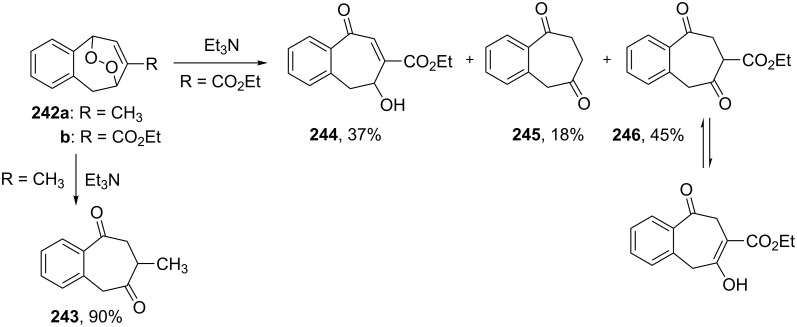
The Kornblum–DeLaMare rearrangement of peroxides **242a**,**b**.

The reaction of endoperoxide **242a** containing an electron-donating substituent at the double bond with bases results in the rearrangement product diketone **243**. Under the same conditions, the base-catalyzed rearrangement of endoperoxide **242b** containing an electron-withdrawing substituent leads to a product mixture of hydroxy ketone **244**, and diketones **245** and **246** (Scheme 75) [353].

**Scheme 76:**
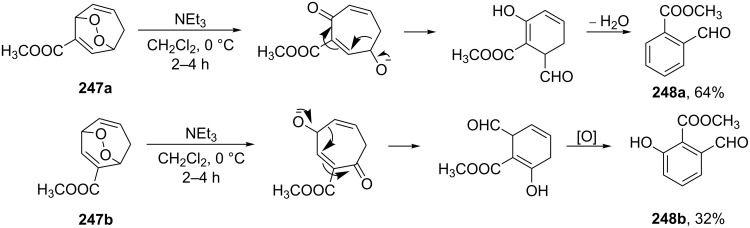
The base-catalyzed rearrangements of bicyclic endoperoxides having electron-withdrawing substituents.

A further study [352] on the base-catalyzed rearrangements of substituted bicyclic endoperoxides showed that the pathway of the rearrangement is largely determined by the position of the substituent. The rearrangement of endoperoxides **247a**,**b** containing an electron-withdrawing substituent in the seven-membered ring occurs mainly via a retro-aldol cleavage giving rise to formyl benzoates **248a**,**b** (Scheme 76).

**Scheme 77:**
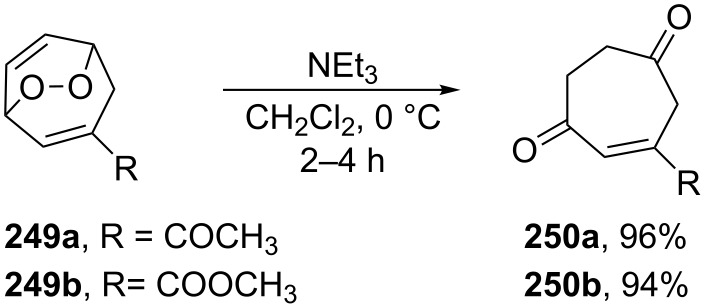
The base-catalyzed rearrangements of bicyclic endoperoxides **249a**,**b** having electron-donating substituents.

On the other hand, endoperoxides **249a**,**b** bearing electron-withdrawing groups (ester, acetyl) attached to the seven-membered ring are isomerized to diketones **250a**,**b** (Scheme 77) [345].

**Scheme 78:**
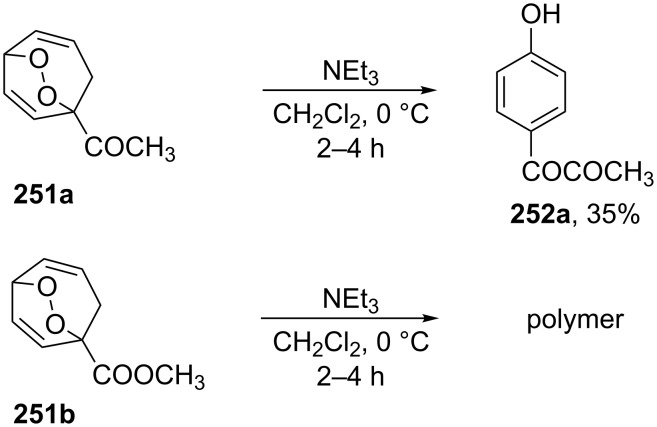
The base-catalyzed rearrangements of bridge-head substituted bicyclic endoperoxides **251a**,**b**.

The Kornblum–DeLaMare reaction of endoperoxide **251a** containing an electron-withdrawing substituent at the bridge head atom lead to the 1,2-dicarbonyl compound **252a** whereas the ester **251b** polymerized upon treatment with triethylamine (Scheme 78).

**Scheme 79:**
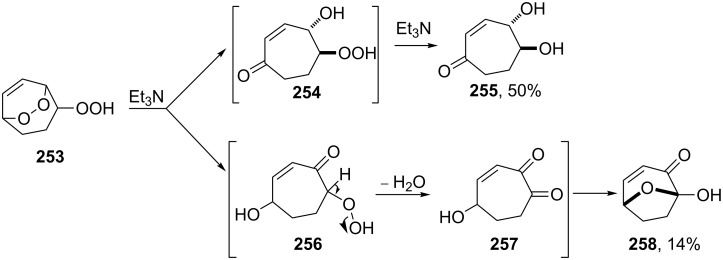
The Kornblum–DeLaMare rearrangement of hydroperoxide **253**.

The disproportionation of endoperoxide **253** promoted by triethylamine affords β- and γ-hydroxy hydroperoxides **254** and **256**. Under these conditions, the reaction afforded oxodiol **255** and diketone **257**, which cyclized to hemiketal **258** as the products (Scheme 79) [354].

**Scheme 80:**
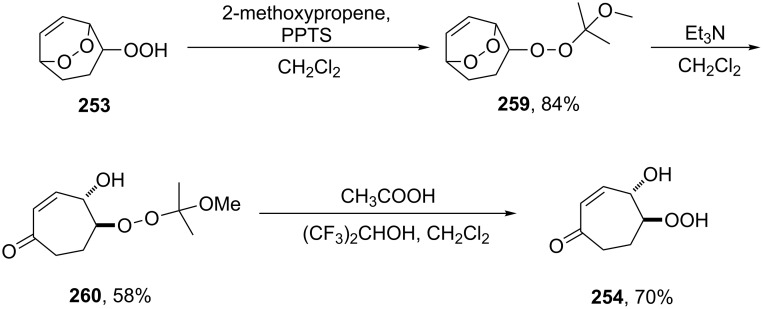
Synthesis of β-hydroxy hydroperoxide **254** from endoperoxide **253**.

As the above reaction did not allow the isolation of hydroperoxide **254**, an alternative strategy towards this compound was developed. The introduction of a protecting group into endoperoxide **253** using 2-methoxypropene gave protected peroxide **259**. The subsequent triethylamine-catalyzed rearrangement of **259** leads to protected intermediate **260** the treatment of which under acidic conditions afforded hydroperoxide **254** in 70% yield (Scheme 80).

One approach to the enantioselective synthesis of 4-hydroxyenones **262** is based on the Kornblum–DeLaMare rearrangement of *meso*-endoperoxides **261** catalyzed by a chiral base [345] (Table 14).

**Table 14:** Enantioselective rearrangement of meso-endoperoxides **261a**–**f** into 4-hydroxy enones **262a**–**f**.

No	Endoperoxide		Reaction conditions^a^	No	Product	Yield,%	eе, %

**261a**	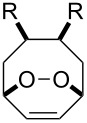	R = H	5 mol % cat., rt, 6 h	**262a**	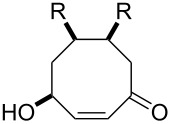	97	99
**261b**		R,R = OC(Me)_2_O	5 mol % cat., rt, 10 h	**262b**		99	99

**261c**	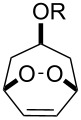	R = H	5 mol % cat., 0 °C, 24 h	**262c**	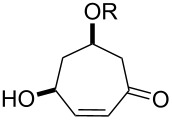	99	87
**261d**		R = TBS	5 mol % cat., rt, 12 h	**262d**		83	99

**261e**	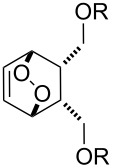	R = Bn	10 mol % cat., rt, 24 h	**262e**	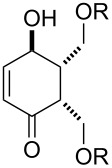	90	96
**261f**		R = -C(Me)_2_-	10 mol % cat., rt, 36 h	**262f**		76	89

^a^cat. =
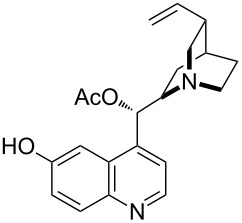

**Scheme 81:**
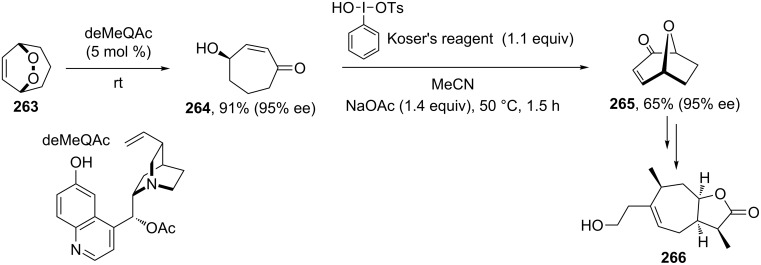
The amine-catalyzed rearrangement of bicyclic endoperoxide **263**.

The amine-catalyzed rearrangement of bicyclic endoperoxide **263** produced (*S*)-(+)-4-hydroxycyclohept-2-en-1-one (**264**), which was oxidized to bicyclic ketone **265**. The synthetic value of chiral bicyclic ketone **265** was demonstrated by the transformation of this compound into (+)-sundiversifolide (**266**) (Scheme 81) [355].

**Scheme 82:**
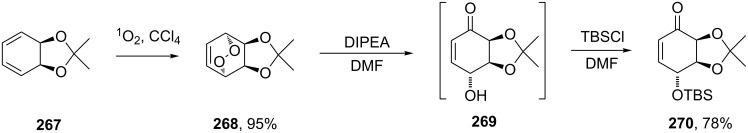
The base-catalyzed rearrangement of *meso*-endoperoxide **268** into **269**.

The photooxidation of diene **267** followed by the base-catalyzed rearrangement of *meso*-endoperoxide **268** lead to (±)-*trans,cis*-4-hydroxy-5,6-di-*O*-isopropylidenecyclohex-2-en-1-one (**269**). The protection of the hydroxy group in compound **269** provides an efficient route to functionalized 4-hydroxy-2-cyclohexene-1-ones **270** (Scheme 82) [356].

**Scheme 83:**
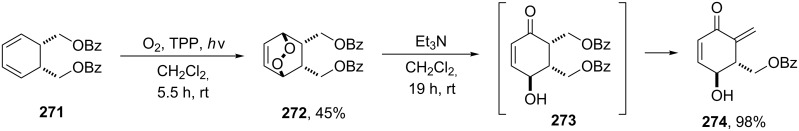
The photooxidation of **271** and subsequent Kornblum–DeLaMare reaction.

The photooxidation of **271** in the presence of tetraphenylporphyrin produces endoperoxide **272,** which undergoes a Kornblum–DeLaMare transformation when treated with triethylamine. The obtained product 4-hydroxycyclohexen-2-one **273** releases benzoic acid through β-elimination under the basic conditions to give cyclohexadienone **274** (Scheme 83) [357].

The base-catalyzed isomerization of bicyclic saturated fulvene endoperoxides **275** is employed as one approach to the preparation of 2-alkenylcyclopentanones **276** and cyclopentenones **277** [358]. Thus, the treatment of a solution of endoperoxides **275** in CH_2_Cl_2_ with triethylamine while increasing the temperature from 0 °C to room temperature affords hydroxyketone **276**. The use of the stronger base DBU results in the formation of 2-vinyl-2-cyclopentenones **277** in high yield (Table 15).

**Table 15:** DBU-catalyzed isomerization−dehydration of saturated fulvene endoperoxides **275** to form 2-vinyl-2-cyclopentenones **277**.

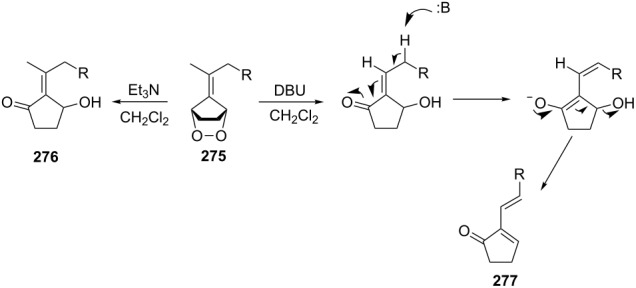		

Endoperoxide	Cyclopentenone	Yield, %

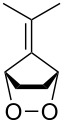 **275a**	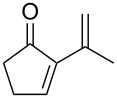 **277a**	76
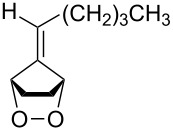 **275b**	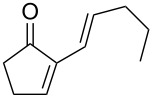 **277b**	83
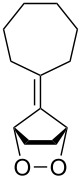 **275c**	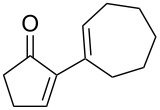 **277c**	82
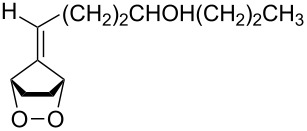 **275d**	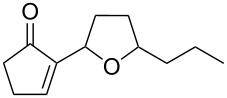 **277d**	68

**Scheme 84:**
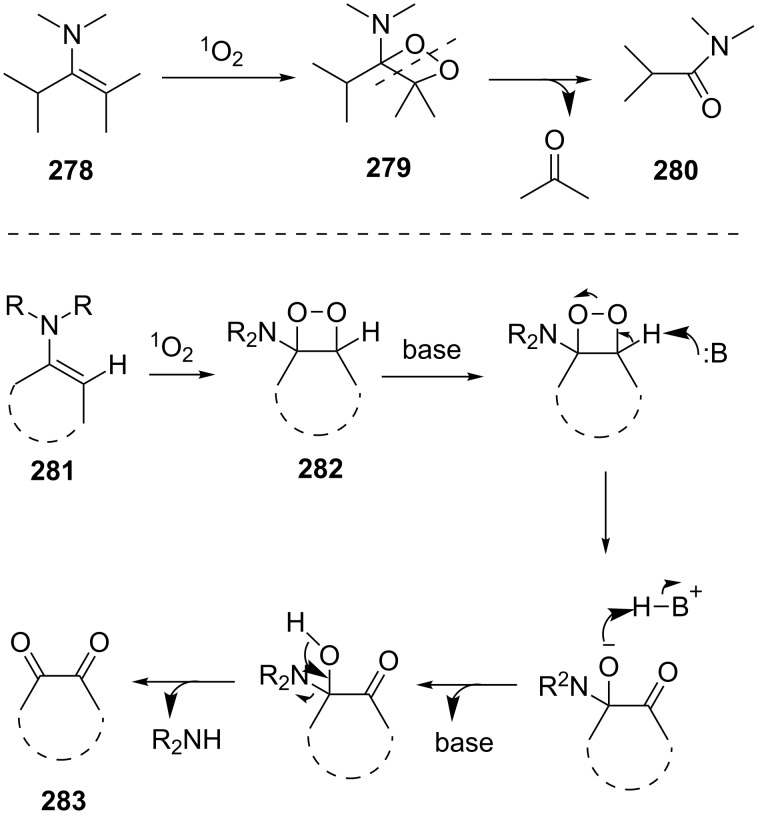
The Kornblum–DeLaMare rearrangement as one step in the oxidation reaction of enamines.

In the case of acyclic enamine **278**, the initial dioxetane product from the photochemical oxidation of **279** rearranged to amide **280**. The reactions using cyclic enamines **281** involve the Kornblum–DeLaMare rearrangement of dioxetanes **282** into 1,2-diketones **283** (Scheme 84) [359–360].

**Scheme 85:**
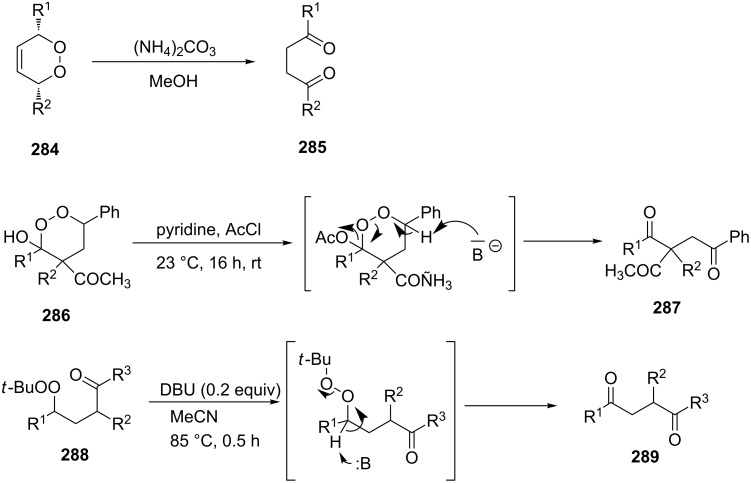
The Kornblum–DeLaMare rearrangement of 3,5-dihydro-1,2-dioxenes **284**, 1,2-dioxanes **286**, and *tert*-butyl peroxides **288**.

The Kornblum–DeLaMare rearrangement of 1,2-dioxenes **284** [361], 1,2-dioxanes **286** [362], and *tert*-butyl peroxides **288** [330,363] produces 1,4-dicarbonyl compounds **285**, **287**, and **289**, respectively (Scheme 85). These compounds are versatile starting substrates for the synthesis of various heterocyclic systems, such as furan, thiophene, and pyrrole derivatives.

**Scheme 86:**
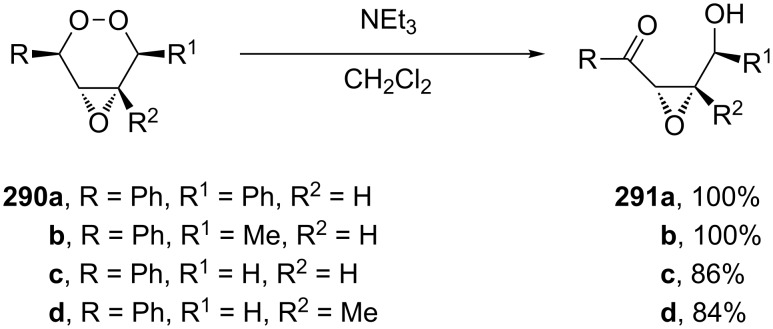
The Kornblum–DeLaMare rearrangement of epoxy dioxanes **290a**–**d**.

The reaction of unsymmetrical epoxy dioxanes **290a**–**d** with triethylamine is accompanied by the 1,2-dioxane-ring opening to form 4-hydroxy-2,3-epoxy ketones **291a**–**d** in high yields. The base catalysis involves the abstraction of the most acidic α-proton in the vicinity of the O–O bond followed by the rearrangement accompanied by the O–O-bond cleavage to form 4-hydroxy-2,3-epoxy ketones (Scheme 86) [364].

**Scheme 87:**
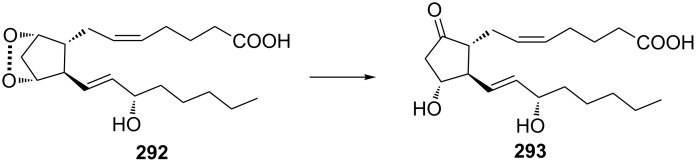
Rearrangement of prostaglandin H_2_ **292**.

The Kornblum–DeLaMare rearrangement is of special synthetic value in view of the synthesis of biologically active compounds. For instance, prostaglandin H_2_ (**292**) containing the bicyclic [2.2.1]endoperoxide moiety is rearranged in situ into prostaglandin Е_2_ (**293**) (Scheme 87) [365–366].

**Scheme 88:**
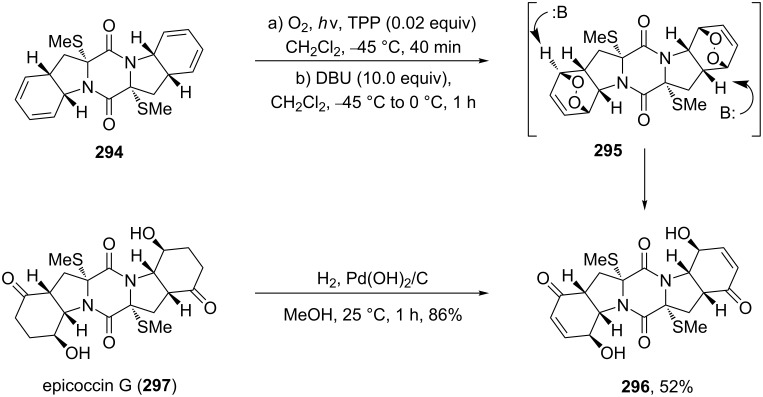
The synthesis of epicoccin G (**297**).

Nicolaou et al. [367] described the synthesis of epicoccin G (**297**) and related diketopiperazines **296** through the photooxidation of **294** and the Kornblum–DeLaMare rearrangement of peroxide **295** (Scheme 88).

**Scheme 89:**
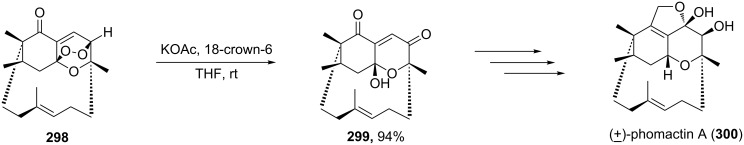
The Kornblum–DeLaMare rearrangement used in the synthesis of phomactin A.

The base-catalyzed transformation of organic peroxide **298** was used to synthesize compound **299**, a precursor for the synthesis of the natural compound phomactin A (**300**). Phomactin A is a representative of a new class of platelet-activating factor (PAF) antagonists (Scheme 89) [368].

**Scheme 90:**
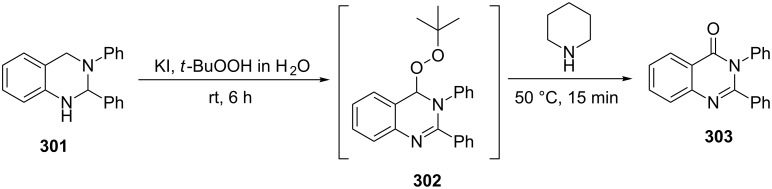
The Kornblum–DeLaMare rearrangement in the synthesis of 3*H-*quinazolin-4-one **303**.

In another study [369], the transformation of peroxide **302**, produced from **301**, was applied to prepare compounds such as 3*H-*quinazolin-4-one **303**, which is a core subunit of some important quinazolinone-based drugs (Scheme 90).

**Scheme 91:**
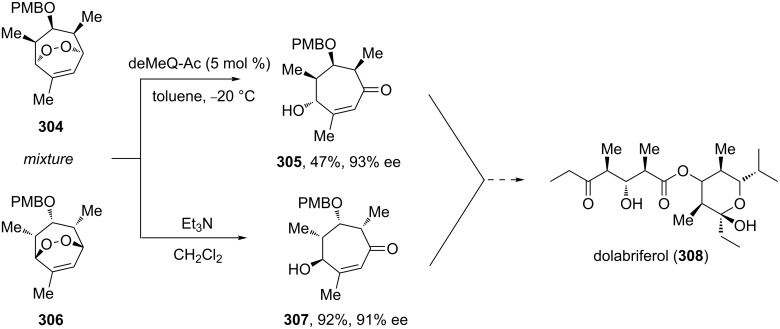
The Kornblum–DeLaMare rearrangement in the synthesis of dolabriferol (**308**).

The Kornblum–DeLaMare rearrangement is one of the steps in the synthesis of the natural compound angelone from *Nauclea*, a plant species widely acclaimed for its anti-inflammatory and antibacterial utilities in traditional Chinese herbal medical formulations [370]. A Kornblum–DeLaMare enantiomeric resolution was also used to obtain both fragments of the polypropionate metabolite dolabriferol from a common precursor. The endoperoxide **304** was converted into ketone **305** with the help of the pseudo-enantiomeric quinine-derived catalyst (deMeQ-Ac) in toluene with moderate 47% yield. The peroxide **306** was transformed into ketone **307** with good 92% yield by using Et_3_N (Scheme 91) [371].

**Scheme 92:**
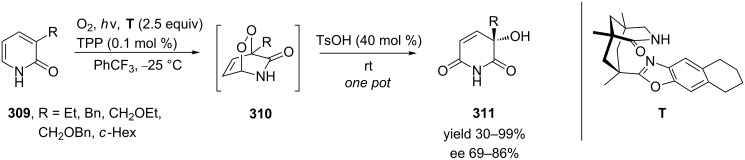
Sequential transformation of 3-substituted 2-pyridones **309** into 3-hydroxypyridine-2,6-diones **311** in one pot.

A sequence consisting of a template-mediated photooxygenation and an acid-catalyzed Kornblum−DeLaMare rearrangement of the intermediate endo-peroxides **310** was used in a one-pot transformation of 3-substituted 2-pyridones **309** into the respective 3-hydroxypyridine-2,6-diones **311** with good enantioselectivity (69–86% ee) (Scheme 92) [372].

**Scheme 93:**
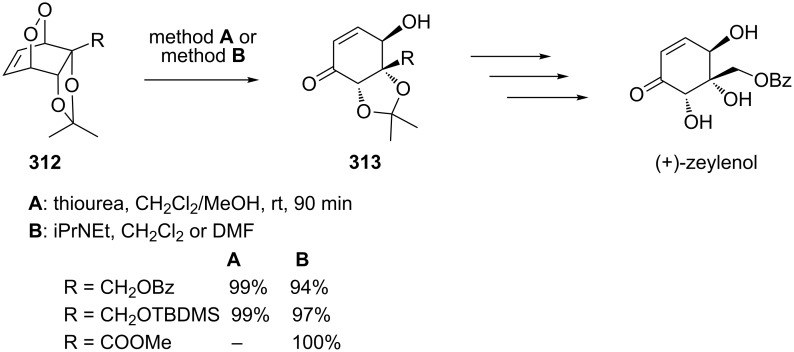
The Kornblum–DeLaMare rearrangement of peroxide **312** into hydroxy enone **313**.

The Kornblum–DeLaMare rearrangement of peroxide **312** into hydroxy enone **313** with high yields and regioselectivity has been reported in the total synthesis of (+)-zeylenol and its congeners (Scheme 93) [373].

**Scheme 94:**
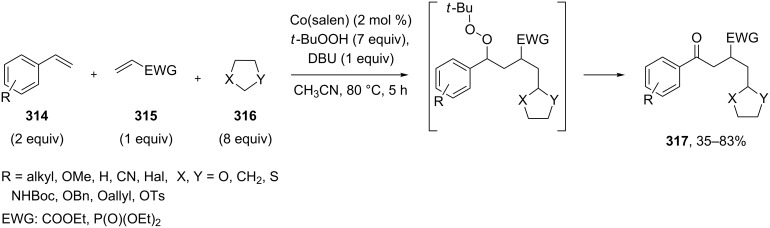
The Kornblum–DeLaMare rearrangement in the synthesis of polyfunctionalized carbonyl compounds **317**.

The polyfunctionalized carbonyl compounds **317** were prepared via crossover oxidative coupling of ethers **316** with electron-deficient alkenes **315** and vinylarenes **314** in the presence of Co(salen) and TBHP under mild conditions. The transformation involved the combination of a tandem radical reaction and a Kornblum−DeLaMare rearrangement in a one-pot process (Scheme 94) [374].

**Scheme 95:**
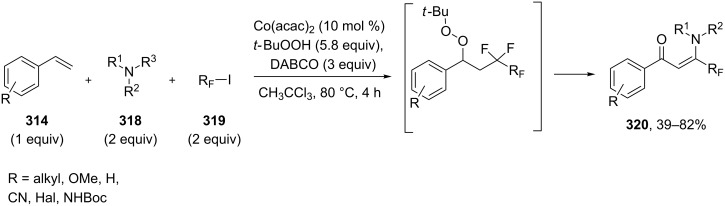
The Kornblum–DeLaMare rearrangement in the synthesis of (*Z*)-β-perfluoroalkylenaminones **320**.

The readily available compounds styrenes **314**, amines **318** and perfluoroalkyl iodides **319** were transformed into (*Z*)-β-perfluoroalkylenaminones **320** via a Co(acac)_2_/TBHP-promoted multicomponent radical reaction involving sequential fluoroalkylation and Kornblum-DeLaMare rearrangement (Scheme 95) [375].

**Scheme 96:**
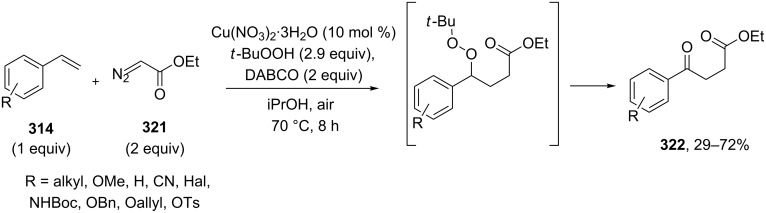
The Kornblum–DeLaMare rearrangement in the synthesis of γ-ketoester **322**.

Peroxy products resulted from the reaction of styrenes **314**, ethyl diazoacetate (**321**), and TBHP underwent a Kornblum–DeLaMare rearrangement with formation of γ-ketoester **322** (Scheme 96) [376].

**Scheme 97:**
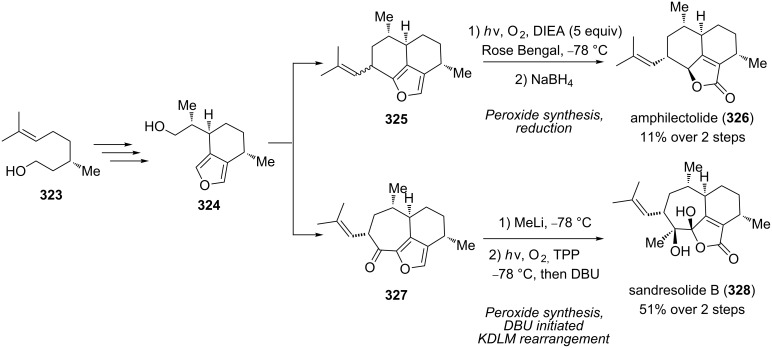
The Kornblum–DeLaMare rearrangement in the synthesis of diterpenoids **326** and **328**.

The Kornblum–DeLaMare rearrangement is a final step in the total synthesis of the diterpenoids amphilectolide (**326**) and sandresolide B (**328**) from a common furan building block **324**, which was synthesized from **323**. Amphilectolide was obtained through a photooxygenation of **325** in the presence of diisopropylethylamine (DIEA), followed by a one-pot reduction of the intermediate peroxide with sodium borohydride. Sandresolide B was prepared from **327** using tetraphenylporphyrin as a photosensitizer and DBU as a base in 51% yield over two steps (Scheme 97) [377].

**Scheme 98:**
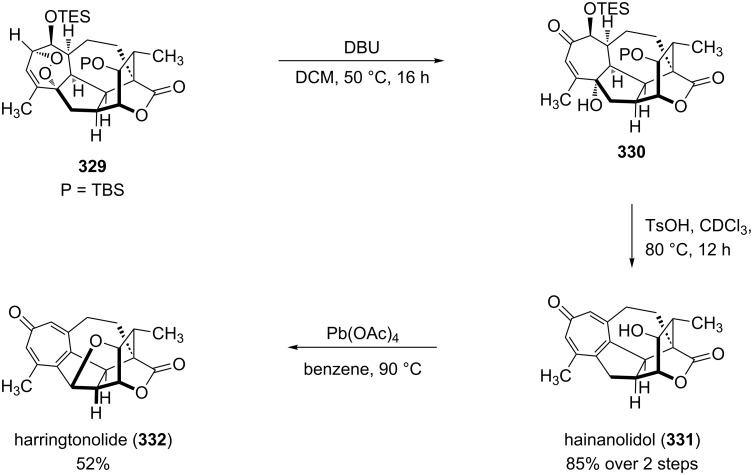
The synthesis of natural products hainanolidol (**331**) and harringtonolide (**332**) from peroxide **329**.

The total synthesis of the natural products hainanolidol (**331**) and harringtonolide (**332**) includes a DBU-promoted Kornblum–DeLaMare rearrangement of endoperoxide **329** to ketone **330** (Scheme 98) [378].

**Scheme 99:**
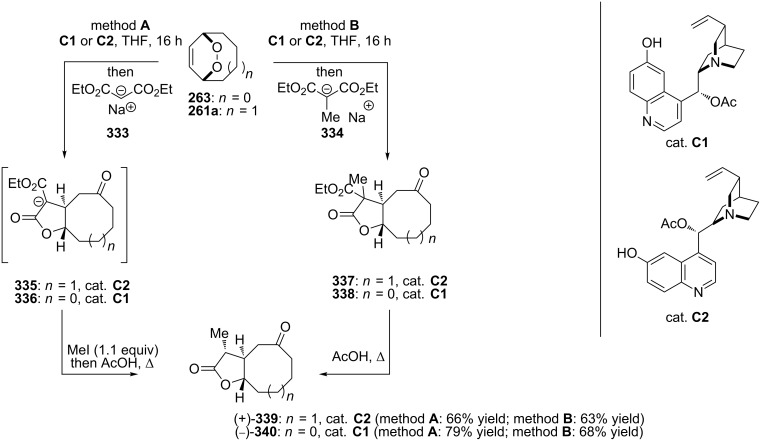
The synthesis of *trans*-fused butyrolactones **339** and **340**.

The reaction of the sodium salts of 1,3-dicarbonyl compounds **333**, **334** with endoperoxides **263** and **261a** in the presence of an organocatalyst affords the *trans*-fused butyrolactones **339** and **340** in high yield. The reaction proceeds via the formation of bicycles **335**, **336** in the case of method A and **337**, **338** in the case of method B (Scheme 99) [379].

**Scheme 100:**
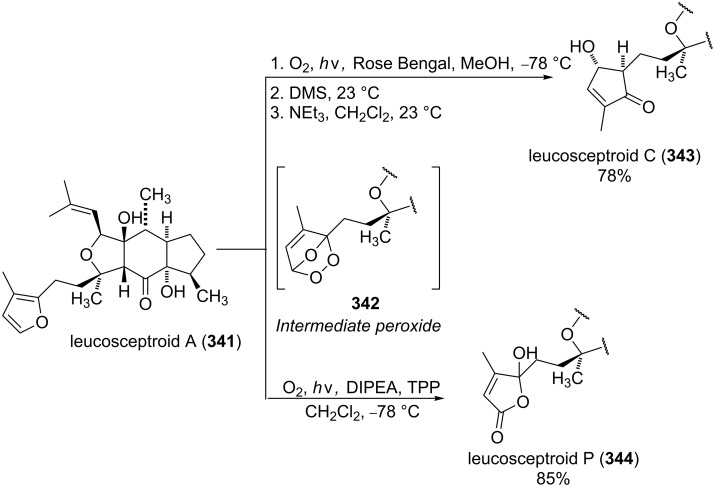
The synthesis of leucosceptroid C (**343**) and leucosceptroid P (**344**) via the Kornblum–DeLaMare rearrangement.

The leucosceptroid A (**341**) produced leucosceptroid C (**343**) and its diastereomer in 78% yield (1:1 dr) under the base-induced reduction of the initial endoperoxide intermediate. Irradiation of a solution of leucosceptroid A (**341**) in an oxygen-saturated dichloromethane solution containing a catalytic amount of tetraphenylporphyrin (TPP) and *N*,*N*-diisopropylethylamine cleanly produced **344** (85% yield). The latter compound represents the base-promoted Kornblum–DeLaMare rearrangement product of endoperoxide **342** (Scheme 100) [380].

It is worth mentioning that the synthesis of 4-hydroxycyclopentenone **343** and litsaverticillols was achieved in a similar way in other works [381–384].

#### 1.5 Dakin oxidation of arylaldehydes or acetophenones

**Scheme 101:**
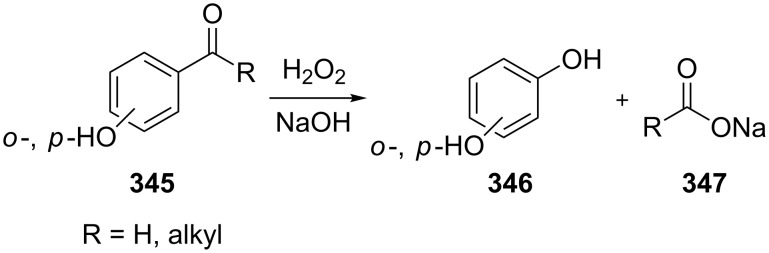
The Dakin oxidation of arylaldehydes or acetophenones.

Generally, the Dakin oxidation is a reaction, in which *o*- or *p*-hydroxylated benzaldehydes or acetophenones **345** react with hydrogen peroxide in the presence of a base to form *o-* or *p*-dihydroxybenzene **346** and carboxylate **347** (Scheme 101) [385–386].

**Scheme 102:**
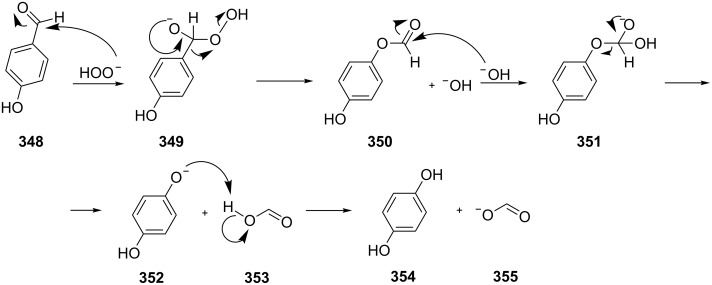
The mechanism of the Dakin oxidation.

Actually, the Dakin oxidation is a special case of the Baeyer–Villiger oxidation. Mechanistically, the Dakin oxidation starts with the nucleophilic addition of a hydroperoxide anion to the carbonyl carbon atom of benzaldehyde (**348**) to form intermediate **349** followed by its rearrangement to phenyl ester **350**. The subsequent nucleophilic addition of a hydroxide anion to the carbonyl group of phenyl ester **350** yields intermediate **351**, which undergoes a rearrangement accompanied by the elimination of phenoxide anion **352** and carboxylic acid **353**. Then, the phenoxide anion **352** deprotonates the carboxylic acid **353** to produce *p*-dihydroxybenzene (**354**) and the corresponding carboxylate anion **355** (Scheme 102) [385,387].

The nucleophilic addition of the hydroperoxide to the carbon atom of a carbonyl group and the [1,2]-aryl migration are the two rate-determining reaction steps in the Dakin oxidation process [387]. The total rate of the Dakin oxidation depends on the nucleophilicity of the hydroperoxide, the electrophilicity of the carbonyl carbon atom, the nature of alkyl substituents in the proximity of the carbonyl group, the existence of other functional groups in the aromatic ring, and the alkalinity of the reaction mixture. Generally, hydroxybenzaldehydes are more reactive in the Dakin oxidation than hydroxyacetophenones. This is due to the fact, that the carbonyl carbon atom of ketones is less electrophilic than the carbonyl carbon atom of an aldehyde. Under weakly basic conditions, *о*-hydroxybenzaldehydes and *о*-hydroxyacetophenones are oxidized more rapidly than *p*-hydroxybenzaldehydes and *p*-hydroxyacetophenones, whereas *m*-hydroxybenzaldehydes and *m*-hydroxyacetophenones are unreactive [387]. Electron-donating substituents in the *ortho* and *para* positions of the aromatic ring enhance the electron density on the migrating carbon atom thus promoting the [1,2]-aryl migration and accelerating the oxidation. Electron-donating substituents in the *meta* position have little effect on the electron density on the migrating carbon atom. Electron-withdrawing substituents in the *ortho* and *para* positions of the aromatic ring reduce the electron density on the migrating carbon atom, interfering with the [1,2]-aryl migration. The hydroperoxide anion is a more reactive nucleophile than neutral hydrogen peroxide. The reaction rate of the oxidation of hydroxyphenylaldehydes or ketones increases with increasing pH value, however, at pH higher than 13.5 the oxidation does not take place [387].

The efficient oxidation of hydroxylated aldehydes and ketones to hydroquinones and catechols was performed using a complex of urea with hydrogen peroxide as an oxidant [388]. The main advantage of this method is, that the reaction is performed under solvent-free conditions and provides the products in high yields.

**Scheme 103:**
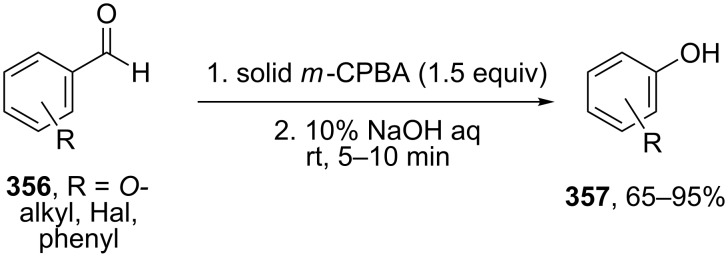
A solvent-free Dakin reaction of aromatic aldehydes **356**.

A solvent-free Dakin reaction of aromatic aldehydes **356** with *m*-CPBA resulted in corresponding phenols **357** with high yields within a few minutes (Scheme 103) [389].

**Scheme 104:**
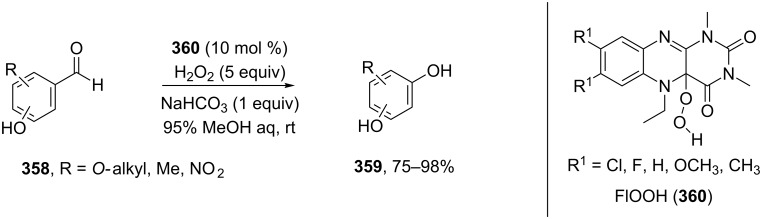
The organocatalytic Dakin oxidation of electron-rich arylaldehydes **358**.

The phenols **359** were prepared from electron-rich arylaldehydes **358** by a flavin-catalyzed Dakin oxidation under the action of H_2_O_2_ and sodium bicarbonate with high yields (Scheme 104) [390].

**Scheme 105:**
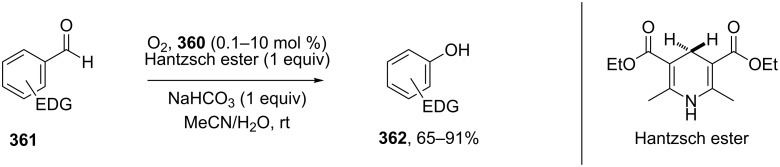
The Dakin oxidation of electron-rich arylaldehydes **361**.

The flavin-catalyzed Dakin oxidation provides a more selective formation of phenols in comparison with the base-catalyzed rearrangement. The Dakin oxidation of arylaldehydes **361** is performed in the presence of molecular oxygen as the oxidant, a flavin organocatalyst and a Hantzsch ester. The oxidation products, catechols and electron-rich phenols **362**, were prepared with 0.1–10 mol % of catalyst, 1 equiv of the Hantzsch ester, and O_2_ or air in a stoichiometric amount (Scheme 105) [391].

**Scheme 106:**
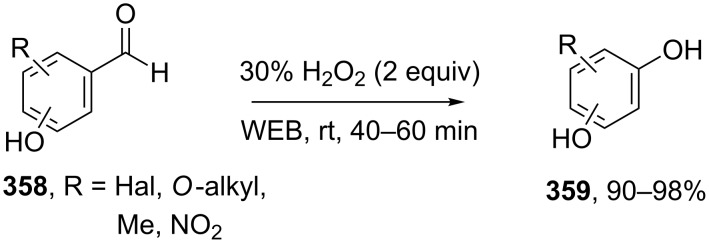
The Dakin oxidation of arylaldehydes **358** in water extract of banana (WEB).

Dakin reactions of benzaldehydes **358** with H_2_O_2_ were successfully performed in natural feedstock extract ‘Water Extract of Banana’ (WEB) at room temperature under aerobic conditions in short reaction times. Under these conditions, phenols **359** could be obtained with 90–98% yields (Scheme 106) [392]. The WEB was prepared by extraction of banana ash with distilled water. The authors suggested that the potassium carbonate and sodium carbonate present in the extract serve as the internal base to promote the Dakin oxidation.

**Scheme 107:**
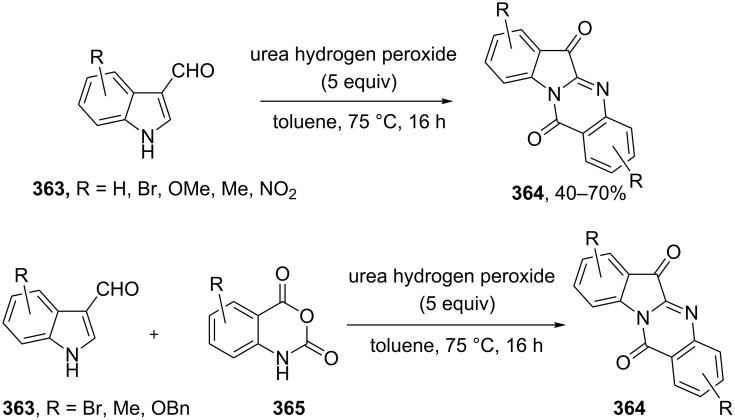
A one-pot approach towards indolo[2,1-*b*]quinazolines **364** from indole-3-carbaldehydes **363** through the Dakin oxidation.

The Dakin oxidation was applied for the synthesis of indolo[2,1-*b*]quinazolines **364** from indole-3-carbaldehydes **363**. In the first step, the oxidation of indole-3-carbaldehydes **363** with further cyclization leads to isatoic anhydrides **365**. Then, the anhydrides **365** react with indole-3-carbaldehydes **363** to produce the target indolo[2,1-*b*]quinazolines **364** (Scheme 107) [393].

The Dakin oxidation is widely used for the synthesis of benzenediols and alkoxyphenols. For example, catechol generated from *o*-alkoxybenzaldehydes is employed as the starting reagent in the synthesis of catecholamine derivatives [394]. Catechols, for example are substrates in the manufacture of synthetic adhesives and coatings. Their multifaceted reactions with both, organic and inorganic reagents, make catechols widely applied compounds for surface modifications [395].

Vanillin was oxidized under Dakin conditions under formation of 2-methoxyhydroquinone with 97% yield. This vanillin-derivative was used as a building block in the synthesis of bio-based compounds applicable in polymer field [396].

The Dakin oxidation of mixtures of lignin depolymerization products is an important process for increasing the number of hydroxy groups in arene cycles. Then, these byproducts are glycidylated with mixtures of epoxy monomers. The obtained products are interesting compounds for the synthesis of bio-based epoxy thermosets with outstanding thermomechanical indexes [397].

**Scheme 108:**
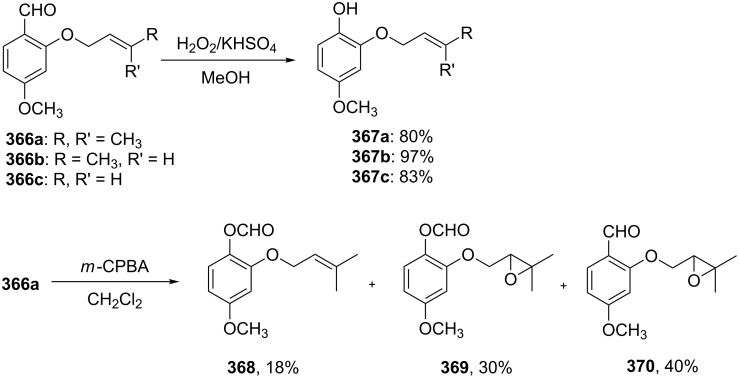
The synthesis of phenols **367a–c** from benzaldehydes **366a-c** via acid-catalyzed Dakin oxidation.

**Acid-catalyzed Dakin oxidation:** The mechanism of Dakin oxidation under mild acidic conditions is similar to the base-catalyzed mechanism. A 30–35% aqueous H_2_O_2_/acid system can be employed as the oxidizing agent to synthesize phenols **367a**–**c** from benzaldehydes **366a–c**. The oxidation of **366a** using traditional peracids produces a mixture of aryl formate **368** and epoxides **369** and **370** (Scheme 108) and cannot be applied to substrates containing peracid-labile functional groups [398].

The addition of boric acid to the H_2_O_2_/acid system leads to an increase in the yield of phenols **372a**–**f** even in the case of benzaldehydes **371a–c** or acetophenones **371d–f** containing electron-donating groups in the *meta* position or electron-withdrawing groups in the *ortho* or *para* positions with respect to the carbonyl group (Table 16) [399].

**Table 16:** Acid-catalyzed Dakin oxidation of benzaldehydes **371a–c** and acetophenones **371d–f** by H_2_O_2_/H_3_BO_3_ in THF.

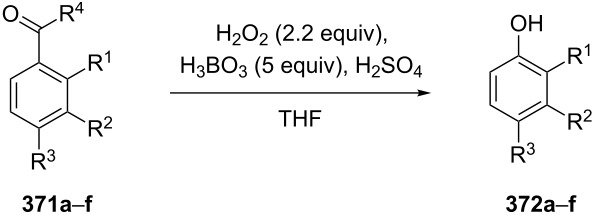			

Compound	Carbonyl compound	Reaction time, h	Yield, % **372a–f**

**371a**	R^1^ = R^2^ = R^3^ = R^4^ = H	12	74
**371b**	R^1^ = OH, R^2^ = R^3^ = R^4^ = H	7	80
**371c**	R^1^ = R^2^ = R^4^ = H, R^3^ = OH	24	90
**371d**	R^1^ = OH, R^2^ = R^3^ = H, R^4^ = Me	36	90
**371e**	R^1^ = R^2^ = R^3^ = H, R^4^ = Me	24	63
**371f**	R^1^ = R^2^ = H, R^3^ = NO_2_, R^4^ = Me	48	60

**Scheme 109:**
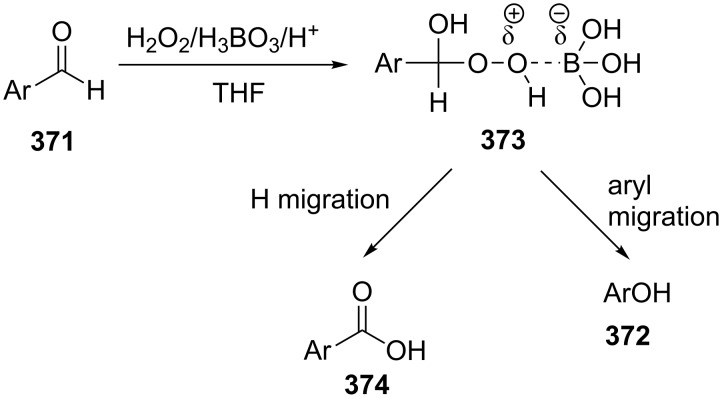
Possible transformation paths of the highly polarized boric acid coordinated H_2_O_2_–aldehyde adduct **373**.

Presumably, the coordination of the H_2_O_2_–aldehyde adduct **373** by the highly polarized boric acid is responsible for the increased yields of phenols **372**. The adduct **373** easily eliminates a borate ion with concerted migration of the aryl group giving phenols **372**. The migrating rate of the aryl group is higher in comparison with hydride migration and formation of **374** (Scheme 109).

#### 1.6 Elbs persulfate oxidation of phenols

**Scheme 110:**
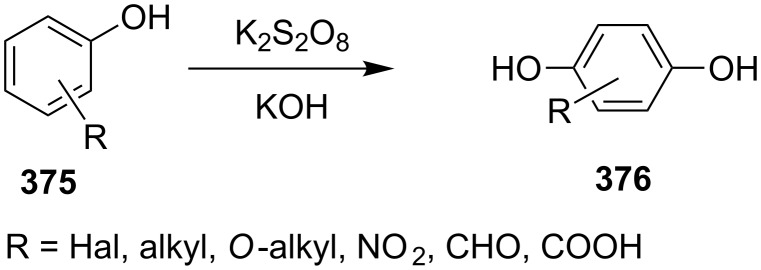
The Elbs oxidation of phenols **375** to hydroquinones.

The Elbs oxidation is the oxidation of phenols **375** with potassium persulfate in the presence of alkali hydroxides to form *p*-hydroquinones **376** (Scheme 110) [400–401].

**Scheme 111:**
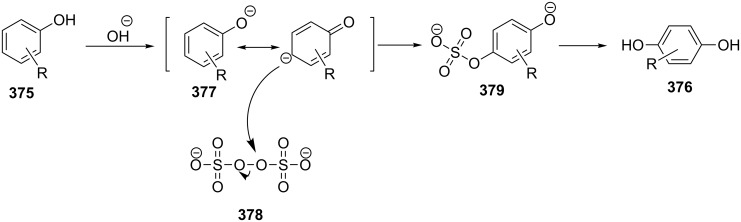
The mechanism of the Elbs persulfate oxidation of phenols **375** affording *p*-hydroquinones **376**.

The Elbs oxidation is a multistep process, which commences by the formation of the phenolate anion **377**. This is followed by the nucleophilic substitution of peroxide oxygen in the peroxydisulfate ion **378** [402] and the resulting sulfoxy group positioned in the *para* position (compound **379**) is hydrolyzed the with formation of *p*-hydroquinone **376** (Scheme 111).

The oxidation of phenols containing electron-donating substituents to dihydroxybenzenes gives products in higher yields compared with phenols containing electron-withdrawing substituents (Table 17) [403–405].

**Table 17:** Oxidation of phenols **375a–f** with potassium persulfate in the presence of alkali.

Phenol	Product	Yield, %	Phenol	Product	Yield, %

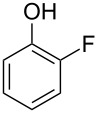 **375a**	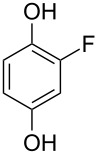 **376a**	47	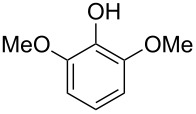 **375d**	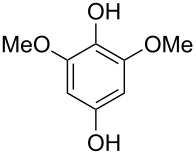 **376d**	69
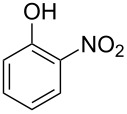 **375b**	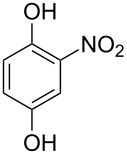 **376b**	35	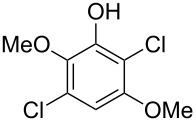 **375e**	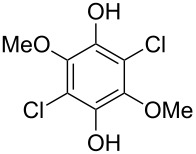 **376e**	42
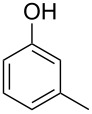 **375c**	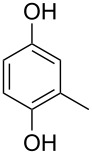 **376c**	66	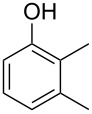 **375f**	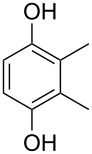 **376f**	49

**Scheme 112:**
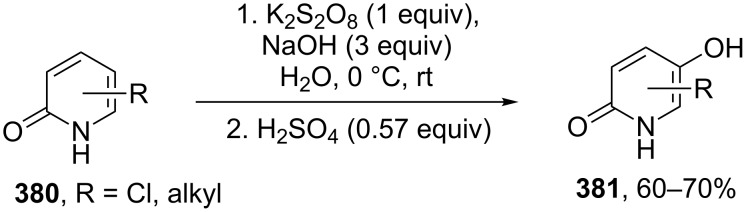
Oxidation of 2-pyridones **380** under Elbs persulfate oxidation conditions.

The main drawback of the persulfate-mediated Elbs oxidation of phenols, are the normally observed moderate conversions and yields. Remarkably, under the above Elbs oxidation conditions 5-hydroxy-2-pyridones **381** were prepared from pyridines **380** with good yields (Scheme 112) [406].

**Scheme 113:**
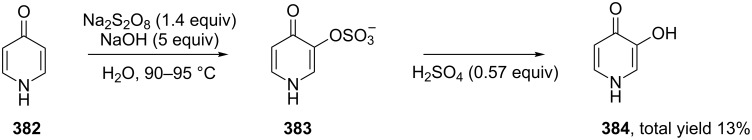
Synthesis of 3-hydroxy-4-pyridone (**384**) via an Elbs oxidation of 4-pyridone (**382**).

Later, the synthesis of 3-hydroxy-4-pyridone (**384**) via the Elbs oxidation of 4-pyridone (**382**) and isolation of 4-pyridone-3-sulfate (**383**) was described (Scheme 113) [407]. The synthesis of 5-hydroxy-6-bromo-2-pyridone was described under similar conditions [408].

#### 1.7 Schenck and Smith rearrangements

**Scheme 114:**
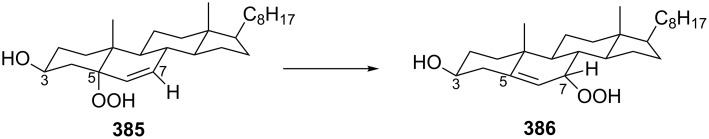
The Schenck rearrangement.

In 1958, Schenck observed that the storage of 5α-hydroperoxide **385** in chloroform for 3 days results in the shift of the OOH group from the 5α to 7α position and a double-bond migration with formation of **386**. This reaction is nowadays known as the Schenck rearrangement (Scheme 114) [409–411].

**Scheme 115:**
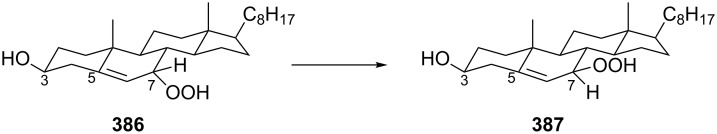
The Smith rearrangement.

In 1973, Smith discovered another type of rearrangement of allylic hydroperoxides [412]. The 7α-hydroperoxide **386** underwent a 20–30% isomerization to the 7β-hydroperoxide **387** if a solution of **386** in ethyl acetate was kept at 40 °C for 48 h (Scheme 115). This process is called the Smith rearrangement.

The mechanisms of these, at first glance simple, reactions were systematically investigated 40 years after their discovery.

**Scheme 116:**
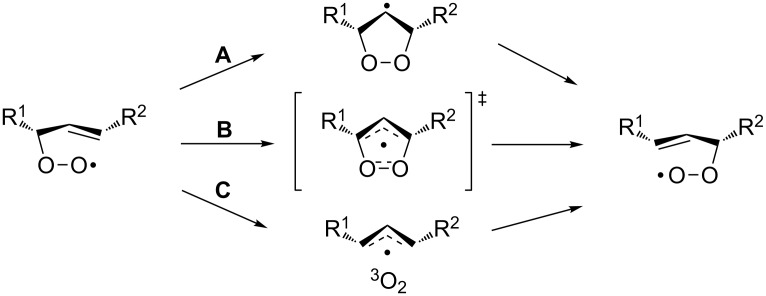
Three main pathways of the Schenck rearrangement.

Three main pathways for the Schenck rearrangement have been proposed (Scheme 116). Path **A** involves the cyclization resulting in the formation of a carbon-centered radical. Path **B** comprises the formation of a transition state with the electron density distributed over a cyclic system. Path **C** proceeds through a dissociation to form an allylic radical and triplet oxygen (Scheme 116) [186,413].

**Scheme 117:**
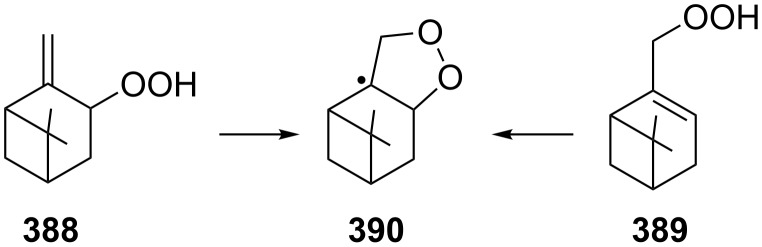
The isomerization of hydroperoxides **388** and **389**.

Path **A**, the initially considered most favorable pathway, was excluded because the isomerization of hydroperoxides **388** and **389** following this route would lead to a β-scission ring opening of **390** (Scheme 117).

**Scheme 118:**
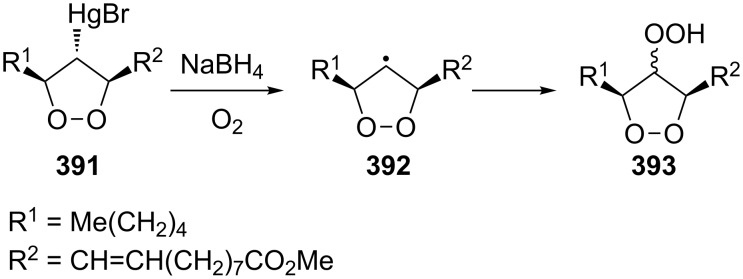
Trapping of dioxacyclopentyl radical **392** by oxygen.

However, this process was not observed and none of the possible carbon-centered radicals **390** was trapped by molecular oxygen [414]. Meanwhile, it is known that the dioxacyclopentyl radical **392** formed from **391** is trapped by oxygen to form hydroperoxide **393** (Scheme 118) [415].

**Scheme 119:**
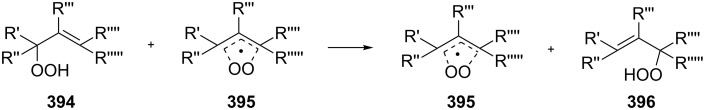
The hypothetical mechanism of the Schenck rearrangement of peroxide **394**.

It was hypothesized that the Schenck rearrangement of peroxide **394** proceeds through a cyclic structure **395** according to the pathway shown in Scheme 119 [414].

However, this hypothesis was also rejected because the ESR spectra recorded after the photolysis of 5α- and 7α-hydroperoxides **385** and **386** showed that the tertiary allylperoxyl radical and secondary allylperoxyl radical are separate and distinct species, and that they do not have the common cyclic structure **395** [416].

**Scheme 120:**
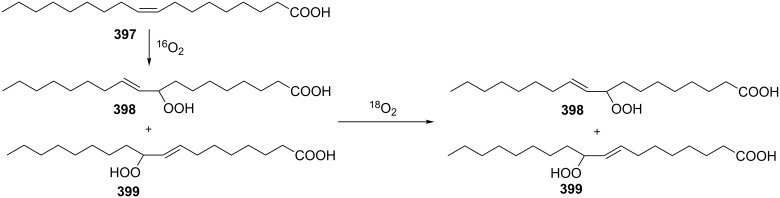
The autoxidation of oleic acid (**397**) with the use of labeled isotope ^18^O_2_.

In a study using labeled isotope ^18^O_2_ it was found that the two hydroperoxides **398** and **399** derived from autoxidation of oleic acid (**397**) underwent the Schenck rearrangement without incorporating dioxygen from the atmosphere (Scheme 120) [417–418]. Later on, Beckwith and Davies confirmed this fact for cholesterol hydroperoxide [416] and the hydroperoxide generated from valencene [419].

Based on these results, no formation of triplet oxygen occurs in the reaction, thus excluding path **C** in Scheme 116. Instead, a cyclic transition state (path **B**, Scheme 116) became more likely, which was confirmed by the stereoselective rearrangement of optically pure olefinic hydroperoxides [420].

However, the study on the rearrangement of hydroperoxides **398**, **399** obtained from oleic acid (**397**) using stereochemical, oxygen-isotopic labeling and solvent viscosity analyses demonstrated that, in hexane, a small amount of atmospheric oxygen is incorporated into the product. The replacement of the solvent by more viscous dodecane and then by octadecane led to a decreased content of atmospheric oxygen in the final product [421–422]. These results provided evidence that the Schenck rearrangement proceeds also through path **C** in Scheme 116.

**Scheme 121:**
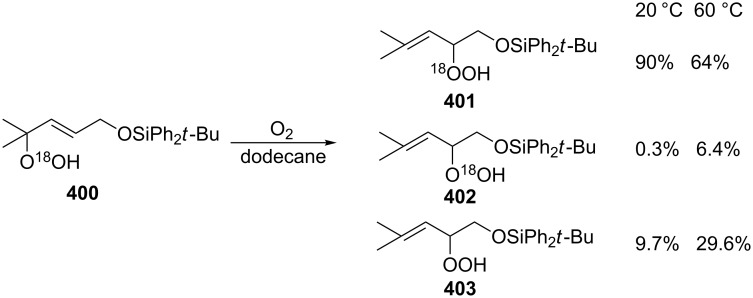
The rearrangement of ^18^O-labeled hydroperoxide **400** under an atmosphere of ^16^O_2_.

Besides, path **C** was also confirmed by the rearrangement of ^18^O-labeled hydroperoxide **400** under an atmosphere of ^16^O_2_ with formation of isotopomers **401**–**403** (Scheme 121) [423].

Examples of the Schenck rearrangement are given in Table 18.

**Table 18:** Examples of the Schenck rearrangement.

Entry	Allylic isomer **A**	Allylic isomer **B**	Ref.

1	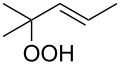 **404a**	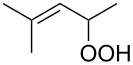 **404b**	[424]
	At 40 °C in non-polar solvents, an approximately equimolar mixture of **A** and **B** is formed		
2	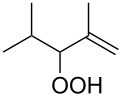 **405a**	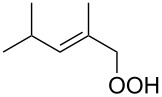 **405b**	[414]
	In hexane, **A** is rearranged to an equilibrium mixture of ~80% **A** and ~20% **B**		
3	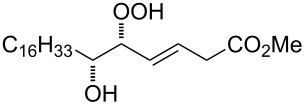 **406a**	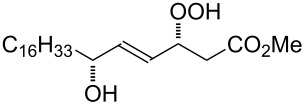 **406b**	[425]
	At 60–70 °C in C_6_H_6_ or MeCN in the presence of TBHN or AIBN within 16–22 h, a 50:50 **A**:**B** mixture is formed		
4	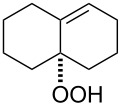 **407a**	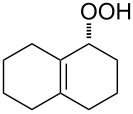 **407b**	[426]
	In CCl_4_ at 40 °C for 141 h, the rearrangement proceeds by 80%		
5	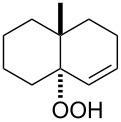 **408a**	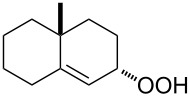 **408b**	[427]
	In CDCl_3_, the rearrangement of **A** into **B** is completed in 24 h		
6	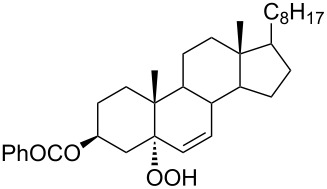 **409a**	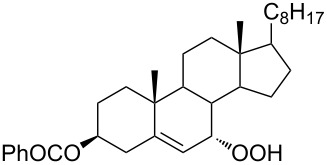 **409b**	[428]
	In CDCl_3_, the rearrangement is completed in 72 h		
7	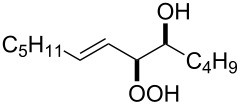 **410a**	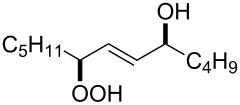 **410b**	[429]
	In C_6_H_6_ in presence of 10 equiv TBHP and 20 mol % DTBN at 40 °C for 16 h, isomers **A** and **B** are formed in equal amounts		
8	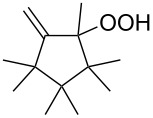 **411a**	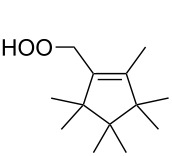 **411b**	[430]
	In CDCl_3_ the rearrangement is completed in 48 h		
9	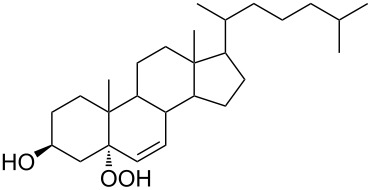 **412a**	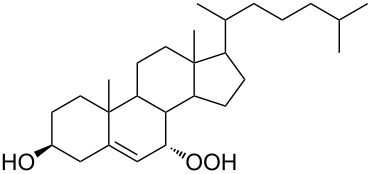 **412b**	[431]
	In CHCl_3_ for 5 d at room temperature, only partial conversion		
10	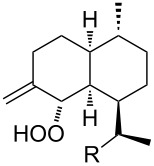 **413a**	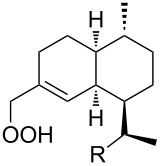 **413b**	[432]
	In CDCl_3_ the rearrangement is completed after 3–4 weeks; R: CO_2_H, CO_2_Me, CH_2_OH, CH_3_		
11	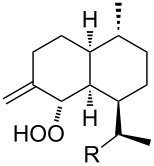 **414a**	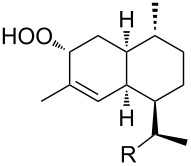 **414b**	
	In CDCl_3_ the rearrangement is completed after 2–4 weeks; R: CO_2_H, CO_2_Me, CH_2_OH, CH_3_		
12	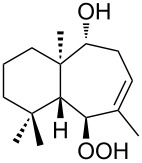 **415a**	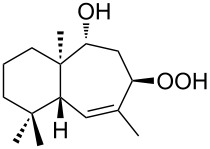 **415b**	[433]
	In CDCl_3_ the rearrangement is completed after 2 d		
13	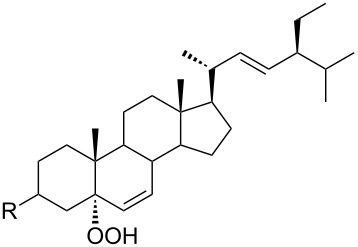 **416a**	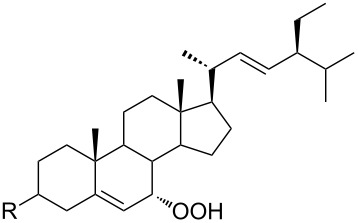 **416b**	[434]
	In pyridine for 24 h, R: OH,CH_3_COO, F, Cl, conversion 12–58%		
14	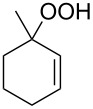 **417a**	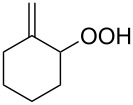 **417b**	[435]
	In a 5 M solution of LiClO_4_ in Et_2_O the rearrangement is completed in 24 h		
15	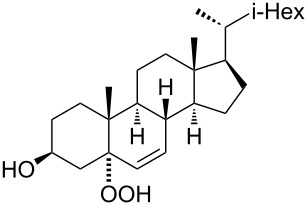 **418a**	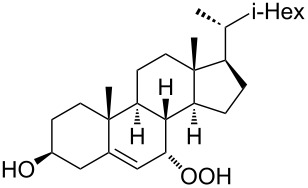 **418b**	[436]
	In CDCl_3_/D_2_O, lyophilized PBS buffer at pH 7 for 20 h, the conversion is 14%		
16	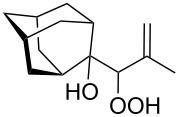 **419a**	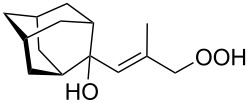 **419b**	[136]
	In CH_2_Cl_2_ at −78 °C with BF_3_·OEt_2_ (1 mol %)		
17	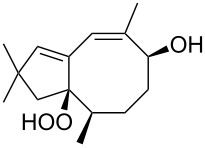 **420a**	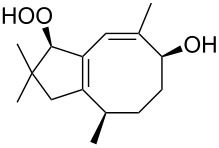 **420b**	[437]
	In MeCN/H_2_O, only partial conversion.		

**Scheme 122:**
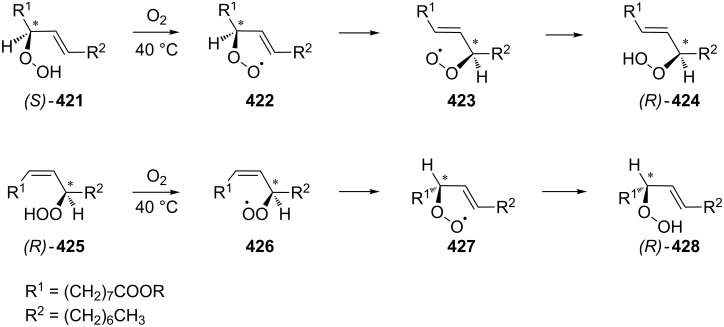
The rearrangement of the oleate-derived allylic hydroperoxides (*S*)-**421** and (*R*)-**425**.

The Schenck rearrangement takes also place with allylic hydroperoxides derived from lipids. The rearrangement of the oleate-derived allylic hydroperoxides (*S*)-**421**, and (*R*)-**425** involved free radicals includes the oxygen-centered radicals **422**, **423**, **426**, **427**. The *E*-oleate hydroperoxide *(S)*-**421** transforms into the corresponding *(R)*-*E*-product **424** at room temperature with a high (*S*) → (*R*) stereoselectivity of more than 97%. A decreased selectivity (~90%) was observed for product **428** obtained from the *Z*-hydroperoxide *(R)*-**425**. In this case, the configurational direction of the reaction was (*R*) → (*R*) (Scheme 122) [438].

**Scheme 123:**
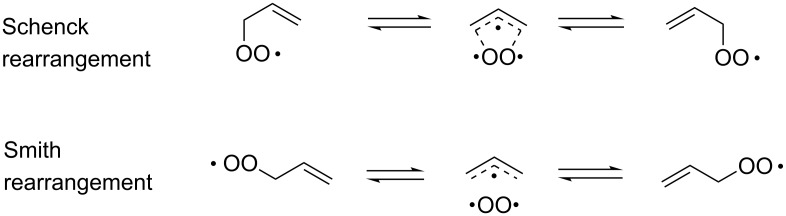
Mechanisms of Schenck and Smith rearrangements.

The Smith rearrangement is a free-radical chain reaction in which atmospheric oxygen may play a greater role than in the Schenck rearrangement. Apparently, the Smith rearrangement proceeds through a dissociation to the allylic radical and ^3^O_2_. Presumably, the distance between these active species is large enough to allow an exchange with atmospheric oxygen (Scheme 123). The Schenck and Smith rearrangements are both a consequence of the reversibility of the reaction of allyl radicals with triplet dioxygen and differ mechanistically in the degree of separation of these two components [186]. There are only a few examples of the Smith rearrangement known and some of them are collected in Table 19.

**Table 19:** Examples of the Smith rearrangement.

Entry	Allylic isomer **B**	Allylic isomer **C**	Comments	Ref.

1	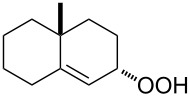 **429a**	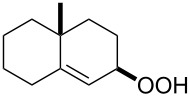 **429b**	In CDCl_3_ within 259 h, approximately 5% of **B** was transformed into **C**	[427]
2	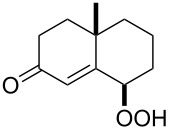 **430a**	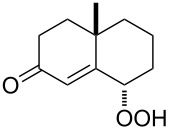 **430b**	In CHCl_3_ at room temperature within 150 h, the **B**:**C** ratio reached 1.8:1	[439]
3	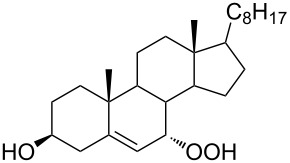 **431a**	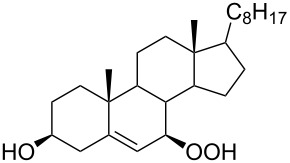 **431b**	In CDCl_3_ at 40 °C within 3.5 h, **B** is transformed into **C** by 20%. In EtOAc at 40 °C, the yield of **C** was 25–30%	[416]
			In CHCl_3_ after 5 d at room temperature, only partial conversion	[431]
4	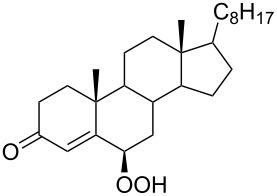 **432a**	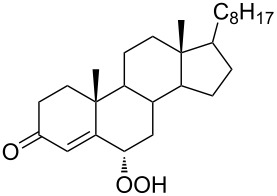 **432b**	In CDCl_3_, the **B**:**C** ratio reached 1:1.5	[439]

**Scheme 124:**
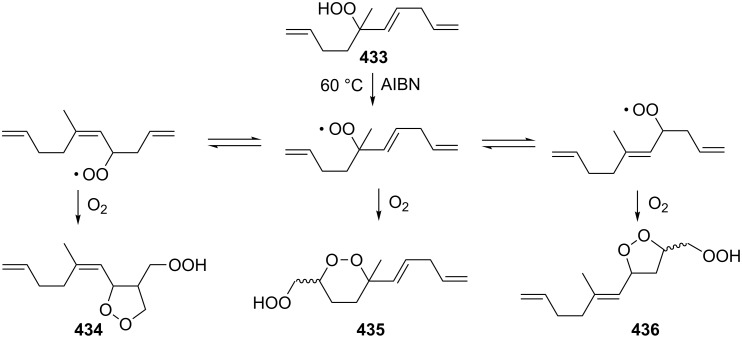
The rearrangement and cyclization of **433**.

In diene or triene-containing systems (**433**), both the rearrangement and cyclization of allylic peroxyl radicals can take place with formation of **434**–**436** (Scheme 124) [440].

#### 1.8 Wieland rearrangement

**Scheme 125:**
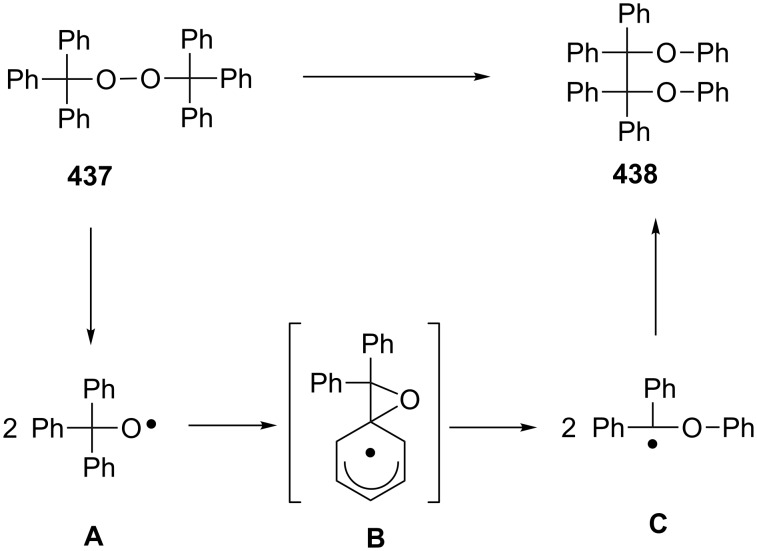
The Wieland rearrangement.

In 1911 Wieland performed the decomposition of bis(triphenylmethyl)peroxide (**437**) under an atmosphere of CO_2_ in boiling xylene for 10 min and obtained the crystalline product **438** in 70% yield (Scheme 125) [441].

The mechanism of the Wieland rearrangement involves the following three steps: Initial formation of O-centered radical **A**, the rearrangement of radical **A** into diphenylphenoxymethyl radical **C**, and the dimerization of radical **C** [442–443].

**Scheme 126:**
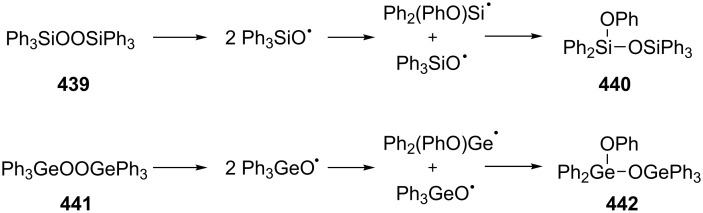
The rearrangement of bis(triphenylsilyl) **439** or bis(triphenylgermyl) **441** peroxides.

Radical 1,2-aryl migrations from silicon or germanium to oxygen is similar to the Wieland rearrangement. The thermal decomposition of either bis(triphenylsilyl) **439** or bis(triphenylgermyl) **441** peroxides leads to the rearranged products **440**, **442** in high yields (Scheme 126) [444–445].

### 2 Unnamed rearrangements of organic peroxides and related processes

#### 2.1 Protic acid-catalyzed rearrangements of organic peroxides and related processes

**Scheme 127:**
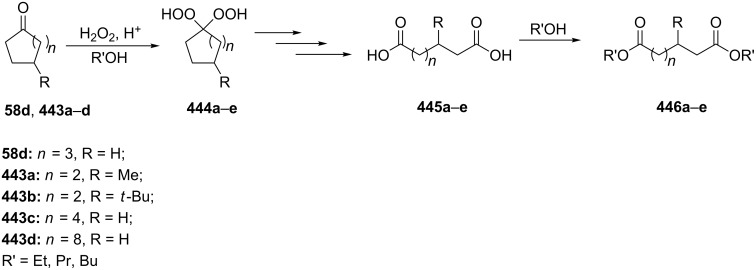
The oxidative transformation of cyclic ketones.

The oxidative transformation of cyclic ketones **58d** and **443a–d** in the reaction with hydrogen peroxide in alcohols in the presence of sulfuric acid proceeds through the formation of geminal dihydroperoxides **444a–e**. The latter compounds are oxidized to dicarboxylic acids **445a–e** followed by their transformation into the corresponding dicarboxylates **446a–e**, rather than formation of lactones via the Baeyer–Villiger reaction (Scheme 127) [446].

This transformation requires the following key conditions to proceed: a reaction temperature higher than 80 °C, the H_2_SO_4_ concentration in the range of 0.2–1.0 mol/L, and a molar ratio of hydrogen peroxide/ketone in the range of 5–10. The corresponding dibutyl esters were prepared in 53−70% yields by oxidation in butanol, which keeps the temperature in the range of 98−106 °C (Table 20).

**Table 20:** Examples of oxidation of ketones **58d**, **443a**–**d** in butanol to diesters **446a**–**e**.

Ketone	Diester	Yield of diester, %	

		aqueous H_2_O_2_ solution	ethereal solution of H_2_O_2_

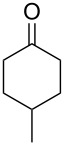 **443a**	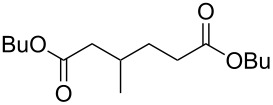 **446a**	59	64
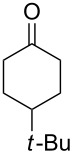 **443b**	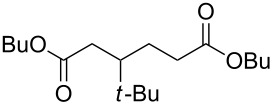 **446b**	57	63
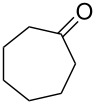 **58d**	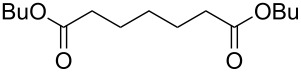 **446c**	62	67
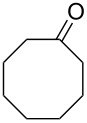 **443c**	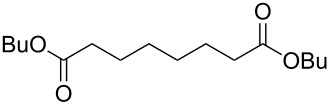 **446d**	61	65
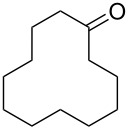 **443d**	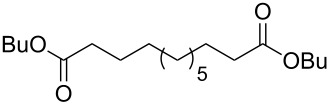 **446e**	64	70

**Scheme 128:**
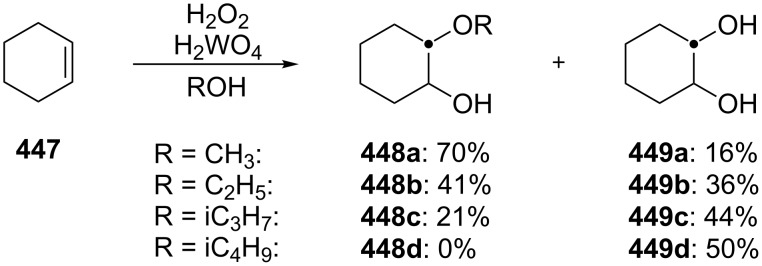
The hydroxylation of cyclohexene (**447**) in the presence of tungstic acid.

In a study on the hydroxylation of compounds containing a double bond to the corresponding α-glycols, the tungstic acid-catalyzed reaction of cyclohexene (**447**) with 90% hydrogen peroxide in methanol, ethanol, or isopropanol afforded the corresponding 2-alkoxycyclohexanols **448a–c** in 70, 41, and 21% yields, respectively, as well as the *trans*-1,2-cyclohexanediols **449a–d** (Scheme 128) [447].

**Scheme 129:**
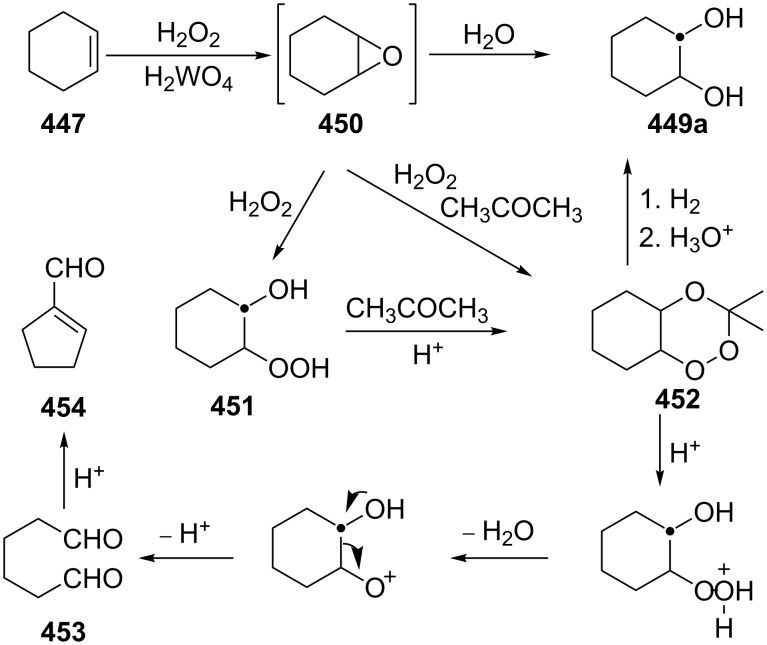
The oxidation of cyclohexene (**447**) under the action of hydrogen peroxide.

A detailed study on the hydroxylation of cyclohexene (**447**) in *tert*-butanol using 30% hydrogen peroxide showed that in this reaction the formation and rearrangements of 2-hydroperoxyalkanols **451** is involved. The treatment of 2-hydroperoxycyclohexanol (**451**) with acetone afforded the cyclic peroxide **452**. The acid-catalyzed rearrangement of the peroxide **452** gave dialdehyde **453**, which further transformed into aldehyde **454**. The isolation and characterization of the latter compound was crucial to an understanding of the oxidation of olefins to aldehydes under the action of hydrogen peroxide (Scheme 129).

**Scheme 130:**
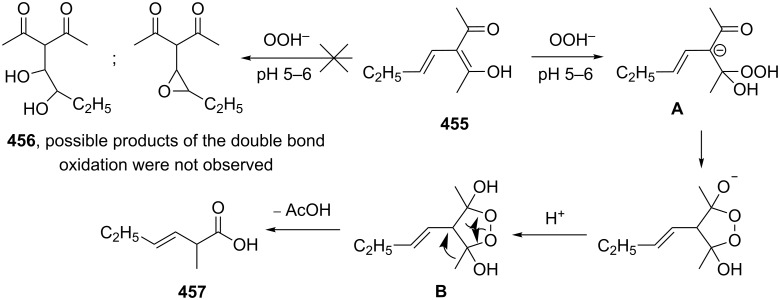
The reaction of butenylacetylacetone **455** with hydrogen peroxide.

The study of the reactions of various unsaturated molecules with hydrogen peroxide demonstrated that the reaction of butenylacetylacetone **455** with H_2_O_2_ at pH 5–6 at 38–40 °C produces 2-methyl-3-hexenoic acid (**457**). Other possible products **456** resulting from a double-bond oxidation reaction were not observed. Apparently, the formation of carbanion **A** is the driving force of this reaction. Carbanion **A** transforms into the symmetrical dihydroxyperoxide **B**, which subsequently rearranges through a deacetoxylation to finally afford 2-methyl-3-hexenoic acid (**457**) (Scheme 130) [448].

**Scheme 131:**
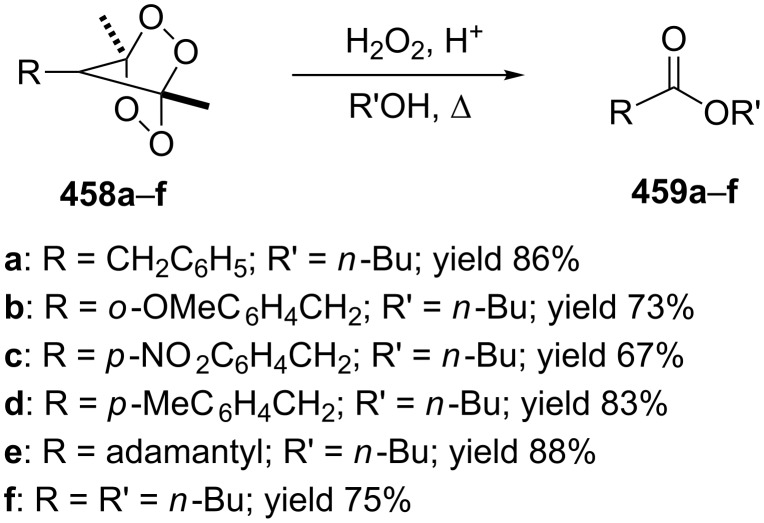
The oxidation of bridged 1,2,4,5-tetraoxanes.

The oxidation of bridged 1,2,4,5-tetraoxanes **458** upon heating in an acidic medium in the presence of H_2_O_2_ is leading to esters **459** (Scheme 131) [449].

**Scheme 132:**
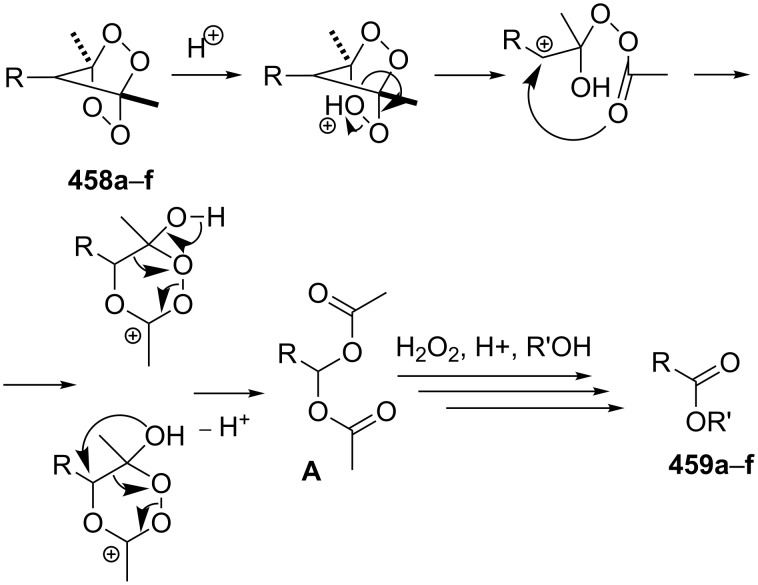
The proposed mechanism for the oxidation of bridged 1,2,4,5-tetraoxanes.

It is assumed that the reaction of tetraoxanes **458a–f** proceeds as an acid-catalyzed oxidative transformation, similar to the Baeyer–Villiger and Hock rearrangements, to yield intermediate **A**. This is further transformed into esters **459a–f** through the oxidation of the CH group and esterification (Scheme 132).

**Scheme 133:**
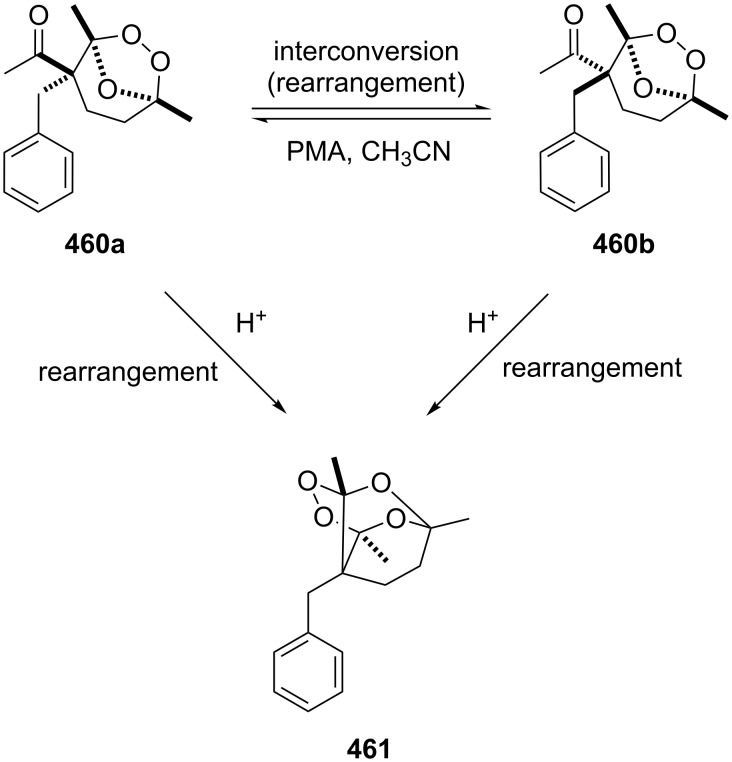
The rearrangement of ozonides.

In another study [450], the rearrangement of isomeric ozonides was described. Here, the ozonides **460a**,**b** were interconverted and rearranged into the tricyclic monoperoxide **461** under the action of phosphomolybdic acid (PMA). This result is attributable to the protic acid nature of PMA as well as its ability to form peroxo compounds containing M–O–O groups that influence the direction of the reaction (Scheme 133).

The observed interconversion of ozonides may be useful for the interpretation of the data on the ozonolysis of unsymmetrical unsaturated compounds.

**Scheme 134:**
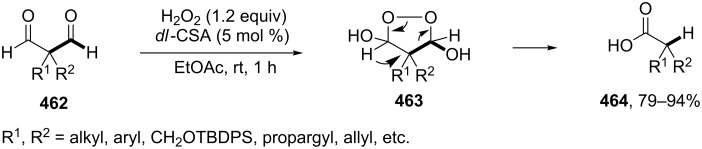
The acid-catalyzed oxidative rearrangement of malondialdehydes **462** under the action of H_2_O_2_.

Carboxylic acids **464** were prepared through a camphorsulfonic acid-catalyzed oxidative rearrangement of a 1,2-dioxolane intermediate **463** prepared from malondialdehydes **462** and H_2_O_2_ (Scheme 134) [451].

#### 2.2 Lewis acid-catalyzed cleavage of peroxides

**Scheme 135:**
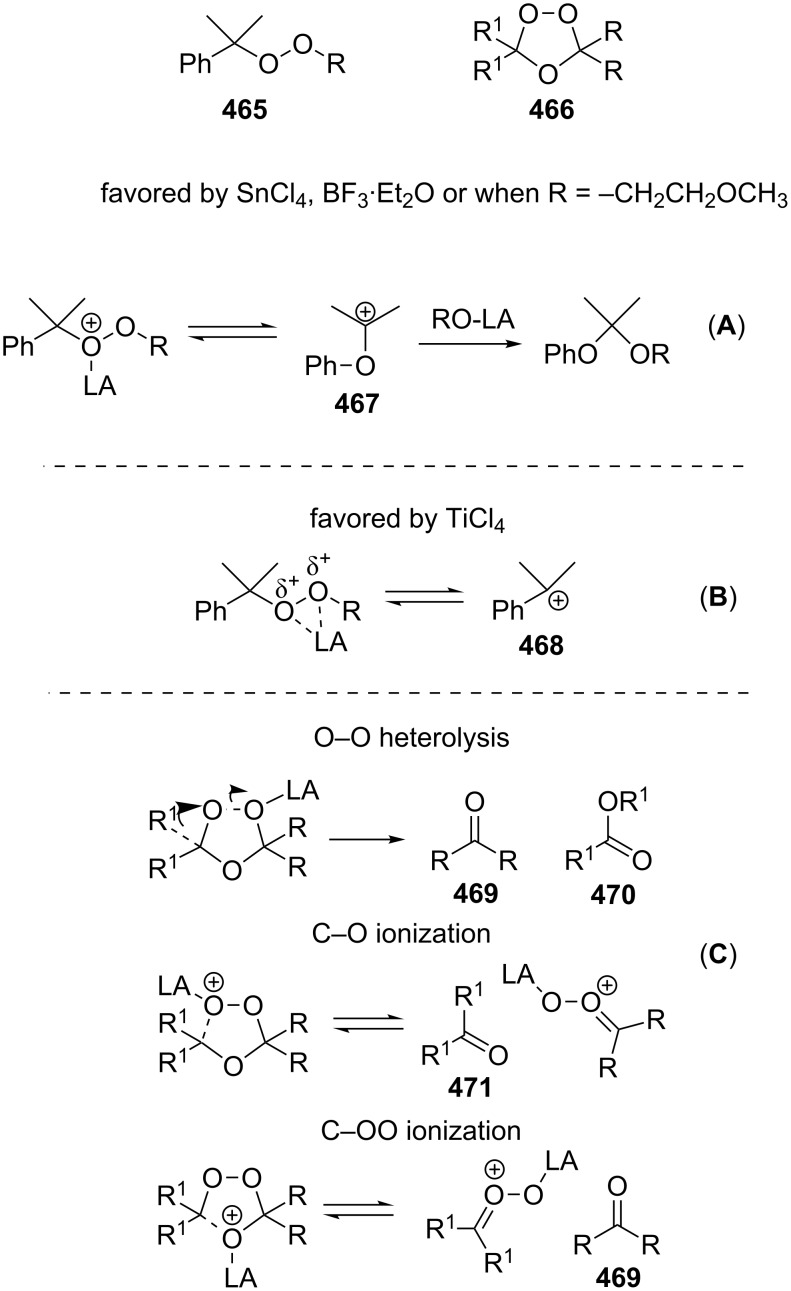
Pathways of the Lewis acid-catalyzed cleavage of dialkyl peroxides **465** and ozonides **466**.

The Lewis acid-catalyzed cleavage of peroxides follows mainly two pathways: the O–O-bond heterolysis to form an oxycarbenium ion **467** accompanied by the migration of the adjacent substituent, and the acid-catalyzed ionization of the C–O bond to yield carbenium ion **468**. The reaction pathway is mainly determined by the nature of the starting compound and the C–O ionization pathway is promoted by the stabilization of the final carbocation, whereas the O–O-bond heterolysis is facilitated by a high migratory ability of the adjacent groups. The fragmentation of dialkyl peroxides **465** and ozonides **466** mainly depends on the nature of the applied Lewis acid. In this way, SnCl_4_ and BF_3_·Et_2_O facilitate the O–O-bond heterolysis (**A**), whereas TiCl_4_ promotes the C–O ionization (S_N_1 mechanism) in tertiary peroxides (**B**). The formation of ketones **469**, **471** and ester **470** is the result of the Lewis acid-catalyzed decomposition of ozonides through the ionization of peroxide, ionization of alkoxide, or oxygen–oxygen heterolysis (**C**) (Scheme 135) [452].

**Scheme 136:**
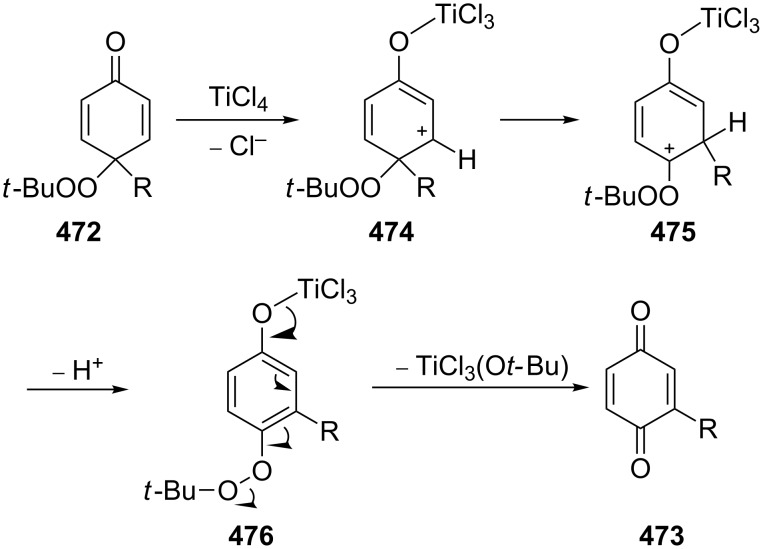
The mechanism of the transformation of (*tert*-butyldioxy)cyclohexanedienones **472**.

The TiCl_4_-promoted rearrangement of (*tert*-butyldioxy)cyclohexanedienones **472a–d**, which are generated by the ruthenium-catalyzed oxidation of phenols with *tert*-butyl hydroperoxide, provides an efficient route to 2-substituted quinones **473a–d** (Table 21) [453–454]. The mechanism of this transformation is depicted in Scheme 136.

**Table 21:** TiCl_4_-promoted rearrangement of (*tert*-butyldioxy)cyclohexanedienones **472a–d**.

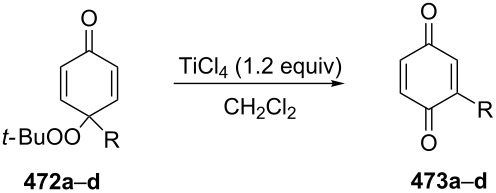			

Peroxide	Reaction conditions	Quinone	Yield, %

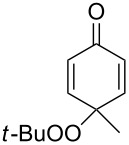 **472a**	25 °C, 1 h	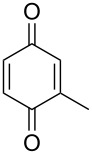 **473a**	92
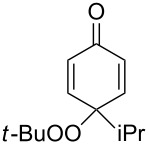 **472b**	−15 °C, 4 h	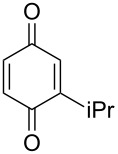 **473b**	98
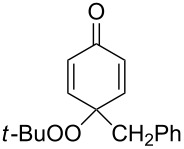 **472c**	−78 °C, 0.5 h	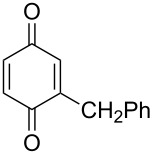 **473c**	93
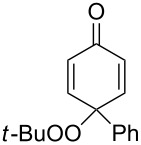 **472d**	−78 °C, 0.5 h	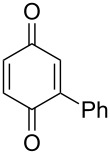 **473d**	91

In the first step, the coordination of dienone **472** to the Lewis acid gives rise to cation **474**. The second step involves a 1,2-alkyl migration to form cation **475.** The subsequent deprotonation of the latter affords aromatic intermediate **476**. In the final step, trichloro-*tert*-butoxytitanium is eliminated from intermediate **476** to produce 2-alkylquinones **473**.

**Scheme 137:**
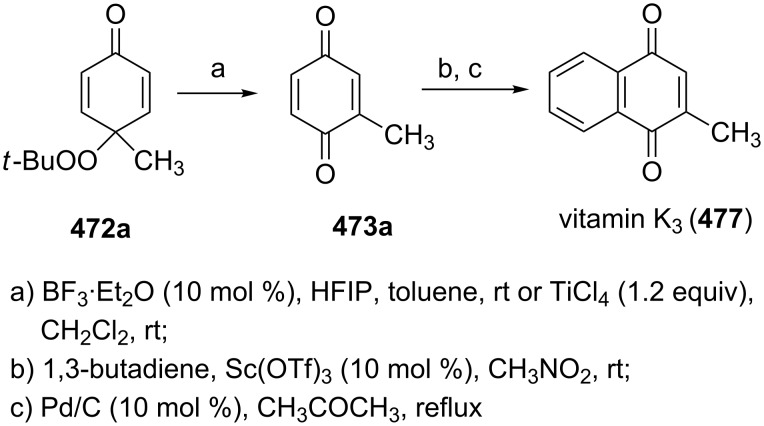
The synthesis of Vitamin K_3_ from **472a**.

The transformation of 4-methyl-4-*tert*-butyldioxycyclohexadienone **472a** into 2-methylbenzoquinone (**473a**) can be used also for the regioselective synthesis of vitamin K_3_ **477** (Scheme 137) [455–456].

The use of SnCl_4_ or TMSOTf as the catalyst made it possible to prepare trimethylsilyl-substituted cyclic peroxides **479a–d** and **480a**,**b** in a *cis* configuration starting from allyltrimethylsilane and bicyclic [2.2.*n*]endoperoxides **478a–d** (Table 22) [457].

**Table 22:** Conditions of the synthesis of trimethylsilyl-substituted cyclic peroxides (1,2-dioxanes) **479a–d** and **480a**,**b**.

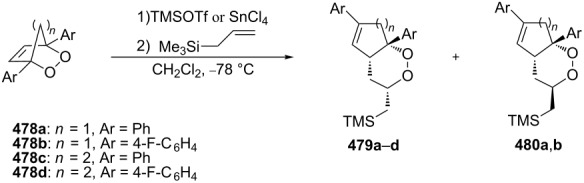				

Endoperoxide	Equivalents of TMSOTf (or SnCl_4_)	Reaction time, min	Product/ratio of diastereomers	Total yield, %

**478a**	0.033	15	**479a/480a**, 1:0	54
**478a**	1.0 SnCl_4_	30	**479a/480a**, 1:1	53
**478b**	1.1	15	**479b/480b**, 1:0.8	60
**478c**	1.1	40	**479c**, 1:0	10
**478d**	1.1	40	**479d**, 1:0	48

**Scheme 138:**
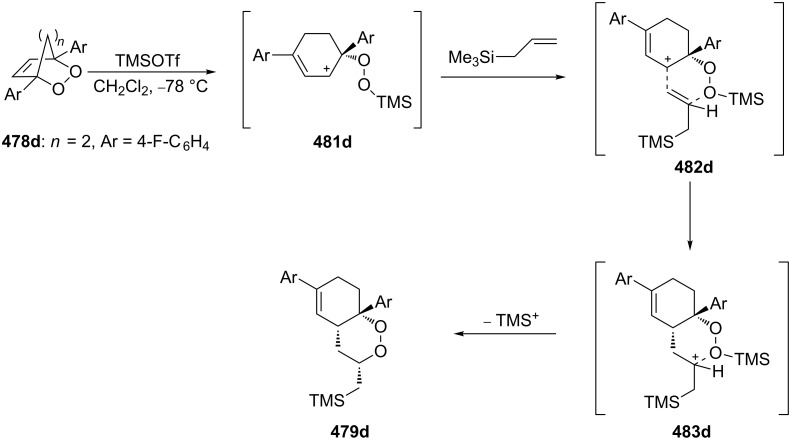
Proposed mechanism for the transformation of **478d** into silylated endoperoxide **479d**.

The mechanism of this reaction implies that TMSOTf or SnCl_4_ promote the heterolytic cleavage of the C–O bond in **478d** to form carbocation **481d**, which is then attacked by allyltrimethylsilane through a chair-like transition state **482d**. The subsequent cyclization of **482d** through the stabilized carbocation **483d** affords silyl-substituted peroxide, 1,2-dioxane **479d**, containing the substituent (–CH_2_SiMe_3_) in the equatorial position (Scheme 138).

**Scheme 139:**
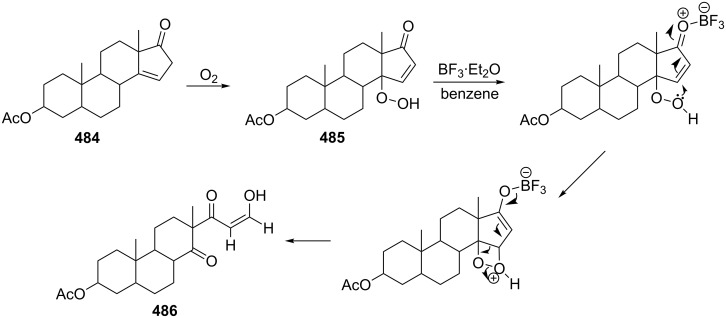
The rearrangement of hydroperoxide **485** to form diketone **486**.

The employment of BF_3_·Et_2_O as the catalyst for the rearrangement of hydroperoxide **485**, which is generated by the oxidation of steroid **484**, enables the opening of the D ring between C-14 and C-16 to form diketone **486** (Scheme 139) [458].

#### 2.3 Rearrangements and related processes of organic peroxides in the presence of bases

**Scheme 140:**
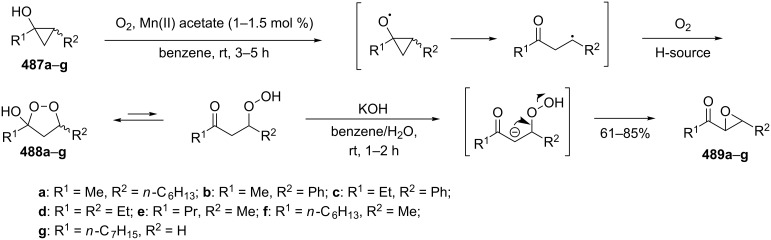
The base-catalyzed rearrangement of cyclic peroxides **488a–g**.

The base-catalyzed rearrangement of cyclic peroxides **488a–g**, which are prepared by the manganese-catalyzed oxidation of 1- and 1,2-disubstituted cyclopropanols **487a–g**, provides a convenient approach to the synthesis of aliphatic and arylaliphatic α,β-epoxy ketones **489a–g**. The latter compounds are attractive substrates for the synthesis of for example natural compounds (Scheme 140) [459].

**Scheme 141:**
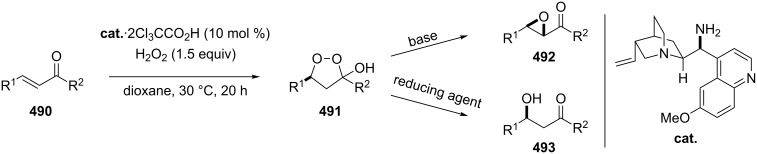
Synthesis of chiral epoxides and aldols from peroxy hemiketals **491**.

Peroxy hemiketals **491** are the starting reagents in the synthesis of epoxides **492** and aldols **493**. Scheme 141 shows the synthesis of epoxides and aldols from inexpensive and readily available α,β-unsubstituted ketones **490** through the intermediate formation of peroxy hemiketal **491** in the presence of a chiral catalyst [460].

**Scheme 142:**
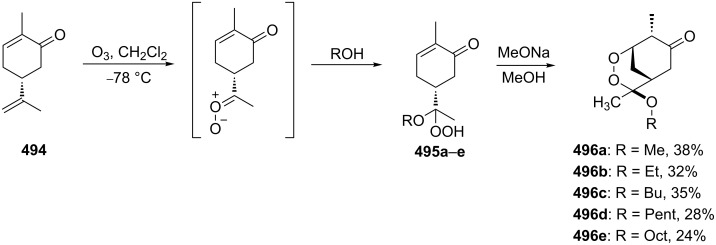
The multistep transformation of (R)-carvone (**494**) to endoperoxides **496a–e**.

A 1:1 mixture of the diastereomeric hydroperoxides **495a–e** was synthesized by ozonolysis of (*R*)-carvone (**494**) and in situ trapping with primary alcohols ROH (R = Me, Et, Bu, Pent, Oct). Further cyclization of these hydroperoxides **495a–e** using the sodium methanolate/MeOH system results in endoperoxides **496a–e** exhibiting antimalarial activity (Scheme 142) [461].

The intramolecular rearrangement of 1,2-dioxetanes **497** containing an aromatic electron-donating substituent is accompanied by emission of light. This process is of special interest for the application in clinical and biological analytical methods, and the synthesis of carbonyl-containing compounds **498** (Table 23) [462–475].

**Table 23:** Base-catalyzed intramolecular rearrangement of 1,2-dioxetanes.

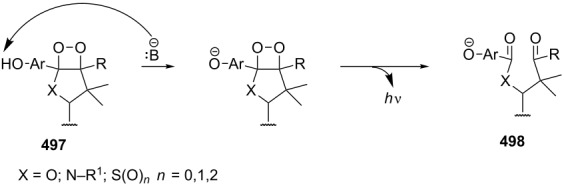					

Entry	X	R	Ar-OH	Reaction conditions	Ref.

1	O	*t*-Bu	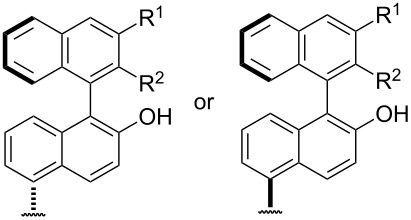 R^1^, R^2^ = H, OMe, CO_2_Me, CO_2_H, CH_2_OH	TBAF in DMSO at 25 °C for 1 h	[463]
2	O	*t*-Bu	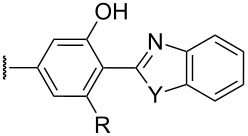 R = H, OMe; Y = O, S	NaOH in СH_3_CN/H_2_O at 45 °C	[464]
3	O	*t*-Bu	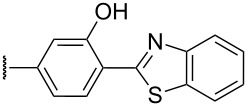	TBAF in DMSO (NMP or DMF) at 45–100 °C	[465]
4	O	Me, Et, iPr, iBu		in NMP at 50–100 °C or in TBAF/NMP at 35–60 °C	[466]
5	O	*t*-Bu	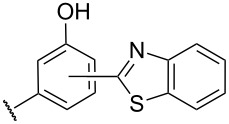	TBAF in CH_3_CN at 45 °C	[467]
6	NBoc	*t*-Bu	3-OH-C_6_H_4_ 3-OMe-C_6_H_4_ 6-OH-C_10_H_6_	TBAF in DMSO at 25 °C	[468–469,473]
7	S, SO, S(O)_2_	*t*-Bu	3-OH-C_6_H_4_ 3-OMe-C_6_H_4_ 3-OAc-C_6_H_4_	TBAF in DMSO at 25 °C	[472]
8	O	*t*-Bu	HO-phenanthrenyl	TBAF in CH_3_CN at 45 °C	[474]
9	O	*t*-Bu	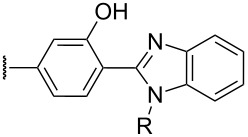 R = H, Me, Ph	TBAF in CH_3_CN or NaOH in H_2_O at 45 °C	[475]
10	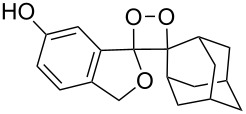			TBAF in DMSO at 25 °C	[470]
11	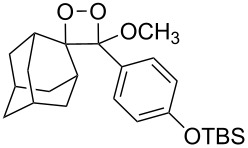			TBAF in THF/DMSO (1:1) at 25 °C	[476]
12	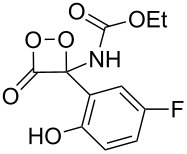			DBU in CH_3_CN at 25 °C	[477]
13	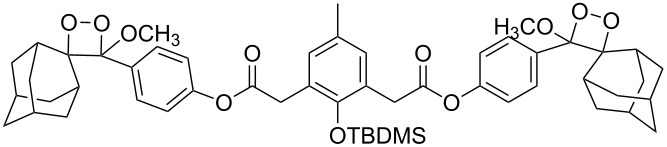			TBAF in DMSO/PBS buffer	[471]

**Scheme 143:**
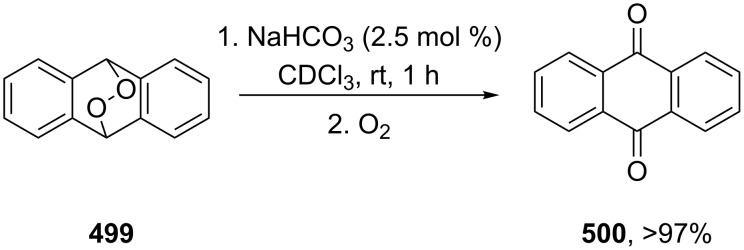
The decomposition of anthracene endoperoxide **499**.

Catalytic amounts of a sodium bicarbonate are sufficient to induce the decomposition of anthracene endoperoxide **499** to anthraquinone (**500**) (Scheme 143) [478].

**Scheme 144:**
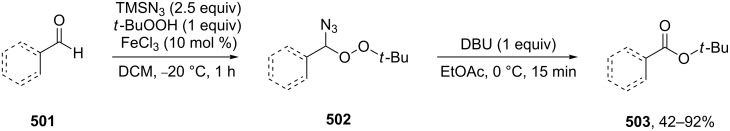
Synthesis of esters **503** from aldehydes **501** via rearrangement of peroxides **502**.

An intramolecular rearrangement of α-azidoperoxides **502** promoted by DBU provides esters **503**. The reaction takes place with alkyl, aryl and heteroaryl α-azidoperoxides generated from the corresponding aldehydes **501** (Scheme 144) [479].

**Scheme 145:**
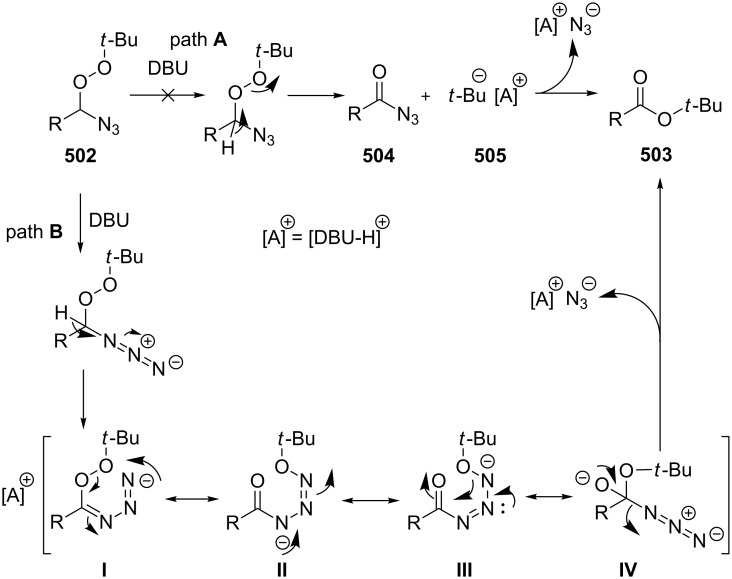
Two possible paths for the base-promoted decomposition of α-azidoperoxides **502**.

There could be two possible paths for base-promoted decomposition of α-azidoperoxides **502** (Scheme 145). The abstraction of the α-hydrogen in the azidoperoxide leads to the direct decomposition of the peroxide bond, which provides acylazide **504** and alkoxide ion **505** (path **A**). Further, the exchange of the azide moiety in the acylazide with an alkoxide ion generates esters **503**. On the other hand, an abstraction of the α-hydrogen in the azidoperoxide leads to a resonance-stabilized intermediate **I** (path **B**). Then, an intramolecular 1,2-alkoxy migration of **I**, via scission of the peroxide bond, followed by cleavage of the C–N bond (intermediate **IV**) affords the desired ester **503**. On basis of control experiments, the reaction is probably following the latter path.

#### 2.4 Thermal and photochemical transformations of organic peroxides

**Scheme 146:**
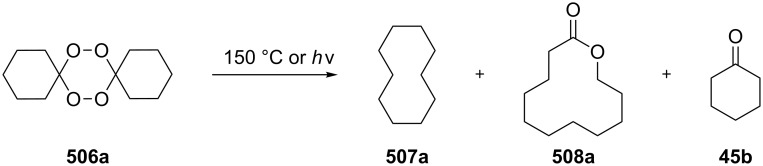
The Story decomposition of cyclic diperoxide **506a**.

**Scheme 147:**
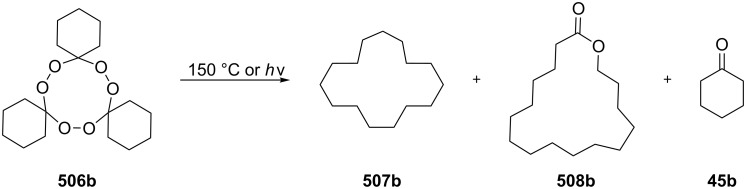
The Story decomposition of cyclic triperoxide **506b**.

Story and co-workers discovered that the thermal and photochemical decomposition of cyclic ketone peroxides **506** produces cycloalkanes **507** and cyclic lactones **508** (Scheme 146 and Scheme 147) [480–483]. This transformation is a general method for the synthesis of macrocyclic compounds from readily available starting materials.

Examples of the thermal decomposition and photolysis of diperoxide **506a** and triperoxide **506b** are given in Table 24.

**Table 24:** Products of thermal and photochemical rearrangement of diperoxide **506a** and triperoxide **506b**.

Peroxide	Conditions		Yields, %	

		Cycloalkane (**507**)	Macrolactone (**508**)	Ketone (**45b**)

**506a**	150 °C, 30 min	44	23	21
	*h*v, MeOH, 3 h	14	10	20
**506b**	150 °C, 30 min	16	<1	15
	*h*v, MeOH, 3 h	15	25	20

**Scheme 148:**
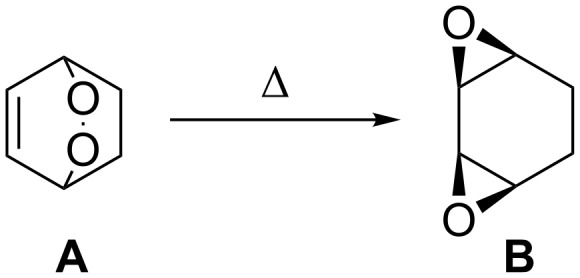
The thermal rearrangement of endoperoxides **A** into diepoxides **B**.

Unsaturated endoperoxides are convenient starting compounds for thermal and photochemical rearrangements. The thermal rearrangement of endoperoxides **A** into diepoxides **B** (Scheme 148) is one of the commonly used transformations [353,484–485].

**Scheme 149:**
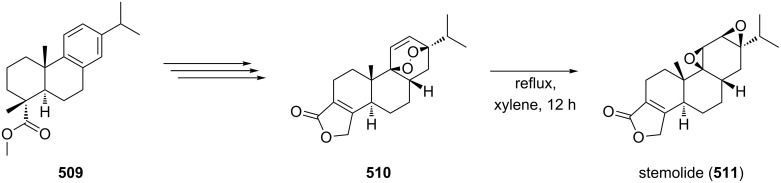
The transformation of peroxide **510** in the synthesis of stemolide (**511**).

The transformation of peroxide **510** is a key step in the synthesis of the cytotoxic agent stemolide (**511)** from methyl dehydroabietate (**509**) (Scheme 149) [486].

It was shown that thermal and photochemical transformations of endoperoxides **261g**, **263**, and **512a–с** afford, in addition to diepoxides **513a–e**, keto epoxides **514a–e** [487–489]. Examples of the thermal decomposition and photolysis of endoperoxides **261g**, **263**, and **512a–c** are given in Table 25.

**Table 25:** Products of thermal decomposition and photolysis of endoperoxides **261g**, **263**, **512a-c**.

Endoperoxide	Diepoxide, **513**	Keto epoxides, **514**	Ratio of products, **513**:**514**

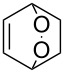 **261g**	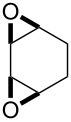 **a**	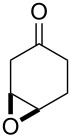 **a**	Δ 36:65 *h*ν 28:72
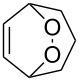 **263**	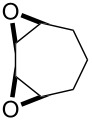 **b**	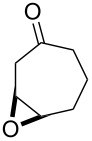 **b**	Δ 90:10 *h*ν 33:67
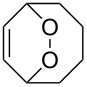 **512a**	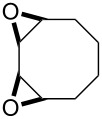 **c**	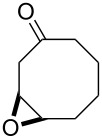 **c**	Δ – *h*ν 35:65
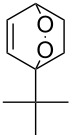 **512b**	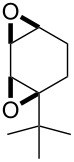 **d**	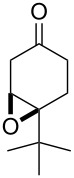 **d**	Δ 65:35 *h*ν 53:37
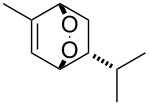 **512c**	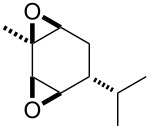 **e**	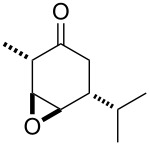 **e**	Δ 58:42 *h*ν 24:76

**Scheme 150:**
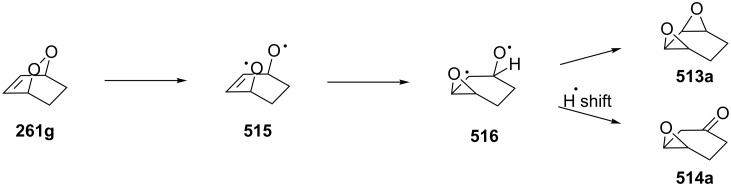
The possible mechanism of the rearrangement of endoperoxide **261g**.

The possible mechanism of the rearrangement of endoperoxide **261g** is shown in Scheme 150. It is supposed that diepoxide **513a** and keto epoxide **514a** are generated from diradical **516** via cyclization of the diradical or a 1,2-hydride shift, respectively. Since 1,4-cyclohexanedione is not generated from endoperoxide, it can be concluded that the first cyclization of **515** to 1,3-biradical **516** occurs rapidly and the formation of epoxide rings takes place successively rather than simultaneously.

**Scheme 151:**
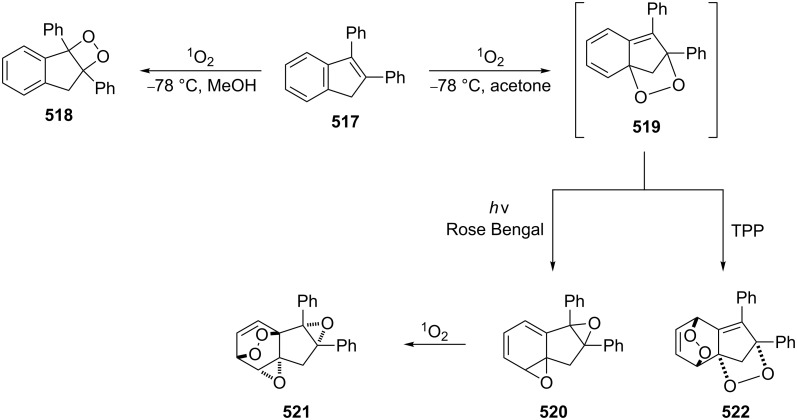
The photooxidation of indene **517**.

The photooxidation of indene **517** without a sensitizer provides dioxetane **518**, in the presence of Rose Bengal, the diepoxyendoperoxide **521** is obtained. Product **521** originates probably from a [2 + 4] addition of singlet oxygen to give **519**, followed by rearrangement to diepoxydiene **520,** which is capable of adding a second mole of oxygen. The use of *meso*-tetraphenylporphyrin instead of Rose Bengal leads to the formation of diendiperoxide **522** (Scheme 151) [484].

**Scheme 152:**
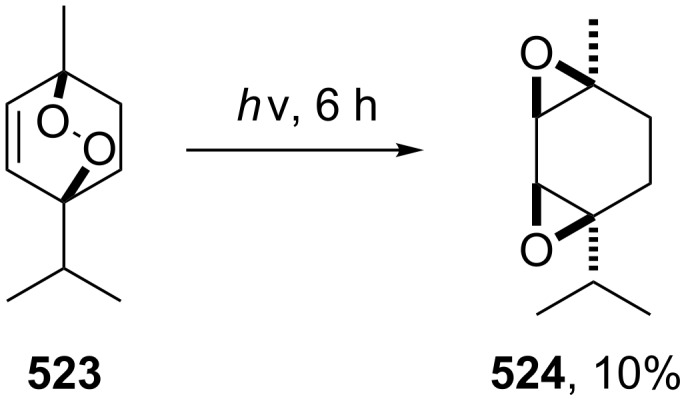
The isomerization of ascaridole (**523**).

Ascaridole (**523**) was slowly isomerized into isoascaridole (**524**) under irradiation with visible light (Scheme 152) [490]. Thermal and photochemical isomerization of related endoperoxides have been applied to the syntheses of other ascaridole analogs [491].

**Scheme 153:**
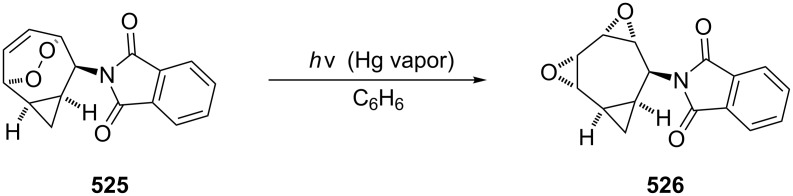
The isomerization of peroxide **525**.

The diepoxide **526** was obtained in 67% yield by photolysis of **525** with a medium-pressure Hg vapor lamp (Scheme 153) [492].

**Scheme 154:**
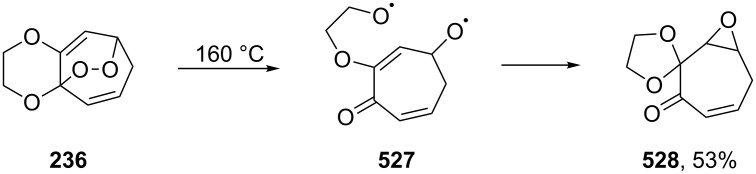
The thermal transformation of endoperoxide **355**.

The thermal transformation of endoperoxides produces mainly bis-epoxides, but can also provide unexpected products such as epoxy ketals. The heating of endoperoxide **236** to 160 °C in toluene affords epoxy ketal **528** in 53% yield through the formation of biradical **527** (Scheme 154) [347].

**Scheme 155:**
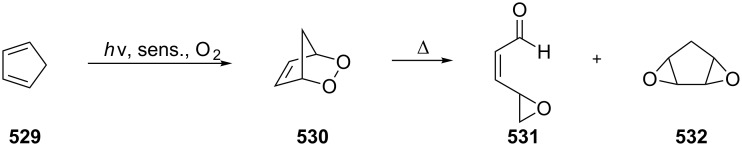
The photooxidation of cyclopentadiene (**529**) at a temperature higher than 0 °C.

The photooxidation of cyclopentadiene (**529**) in an alcohol solution in the presence of polymerization inhibitors at a temperature higher than 0 °C gave *cis*-4,5-epoxy-2-pentenal (**531**) in 58% yield, *cis*-1,2,3,4-diepoxycyclopentane (**532**) as a byproduct (in 7% yield), and polymers instead of the expected peroxide **530** (Scheme 155) [344].

The extensive development of methods for the synthesis of cyclopentenones lies in the fact that this structural unit is present in some natural compounds, such as dihydrojasmone, prostaglandins, and rethrolones. The mechanism of thermal decomposition of saturated fulvene endoperoxides **533a–d** involves the formation of one of the three intermediates **A**, **B**, **C**, which are precursors to cyclopentenones **534a–d** (Table 26) [493].

**Table 26:** Synthesis of cyclopentenones **534a**–**d** from saturated fulvene endoperoxides **533a–d**.

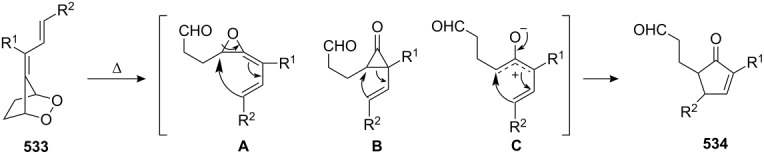		

Endoperoxide	Product	Yield (*trans*:*cis*)

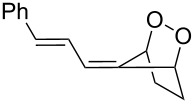 **533a**	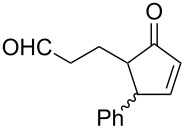 **534a**	85% (8:1)
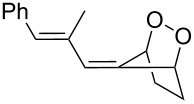 **533b**	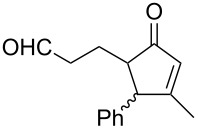 **534b**	83% (2:1)
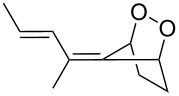 **533c**	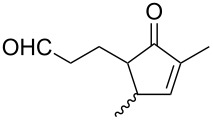 **534c**	90% (6:1)
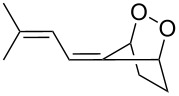 **533d**	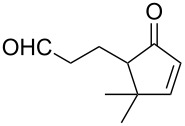 **534d**	68%

The replacement of the vinyl group at the exocyclic double bond in the fulvene precursor by a 3-butenyl group and the thermal decomposition of the resulting endoperoxides **535** at 80 °C lead to a [3,4]-sigmatropic shift of the 3-butenyl group and formation of the 5-oxo-6-heptenal derivatives **536**. The mechanism of this process involves the formation of epoxide **A**, which undergoes a [3,4]-shift through the intermediate **B** [494] (Table 27).

**Table 27:** The mechanism and results of the thermal rearrangement of saturated fulvene endoperoxides **535a–d**.

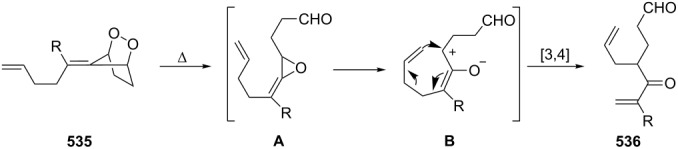		

Endoperoxide	Product	Yield, %

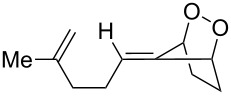 **535a**	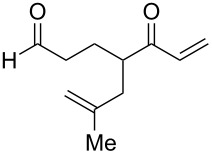 **536a**	45
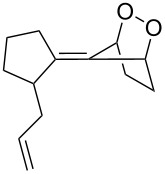 **535b**	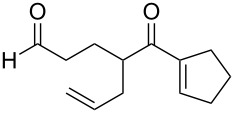 **536b**	83
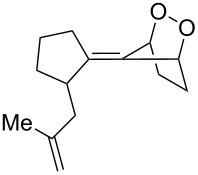 **535c**	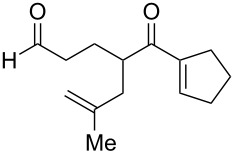 **536c**	85
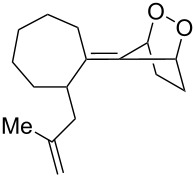 **535d**	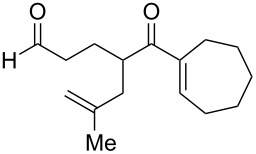 **536d**	79

**Scheme 156:**
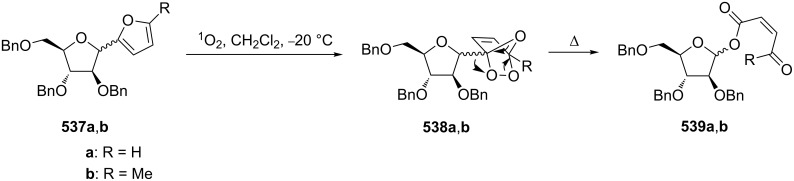
The thermal rearrangement of endoperoxides **538a**,**b**.

The thermal rearrangement of endoperoxides **538a**,**b**, which are generated by the photooxidation of furanosyl furans **537a**,**b**, selectively affords glycosides **539a**,**b** (Scheme 156) [495].

The methylene blue-sensitized photooxidation of arabinofuranosyl furan **537a** as an 1:6 α,β-anomeric mixture at −20 °C followed by warming of the reaction mixture to room temperature produced furanoside **539a** as an anomeric mixture in the same molar ratio. The photooxidation of pure β-arabinofuranosyl furan **537a** produced exclusively β-furanoside **539a**. Based on these data, the intermediate endoperoxide **538a** originates from the cycloaddition of ^1^O_2_ to the furanosyl furan. The selective thermal rearrangement of endoperoxide **538a**, which is similar to the Baeyer–Villiger rearrangement with the retention of the configuration, results in the corresponding O-derivatives.

The thermally unstable endoperoxides **541a–d** generated from 2-alkoxyfurans **540a–d** rearrange through several pathways depending upon the nature of the substituent at the carbon atom C5 in **541** with formation of **542** or **543** (Table 28) [496].

**Table 28:** Results of the rearrangement of endoperoxides **541a**–**d**.

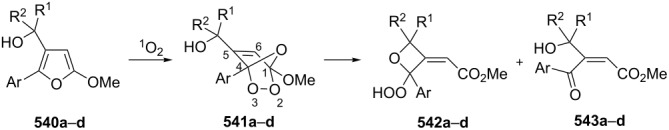					

Compound	R^1^	R^2^	Ar	Yield of **542**, %	Yield of **543**,%

**a**	Et	Et	Ph	88	traces
**b**	Et	Et	4-Br-C_6_H_4_	92	traces
**c**	Ph	Ph	4-Br-C_6_H_4_	93	traces
**d**	Me	H	Ph	0	87

The rearrangement of endoperoxides **541a–c** containing a substituent with a tertiary hydroxy group in the 5 position results in the formation of hydroperoxyoxetanes **542a–c** and trace amounts of *Z*-ketoesters **543a–c**. Under the same conditions, the rearrangement of endoperoxide **541d** containing a substituent with a secondary hydroxy group in the 5 position produces exclusively the *Z*-keto ester **543d** [497].

**Scheme 157:**
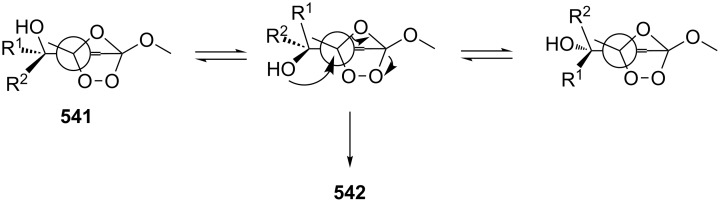
The transformation of peroxides **541**.

**Scheme 158:**
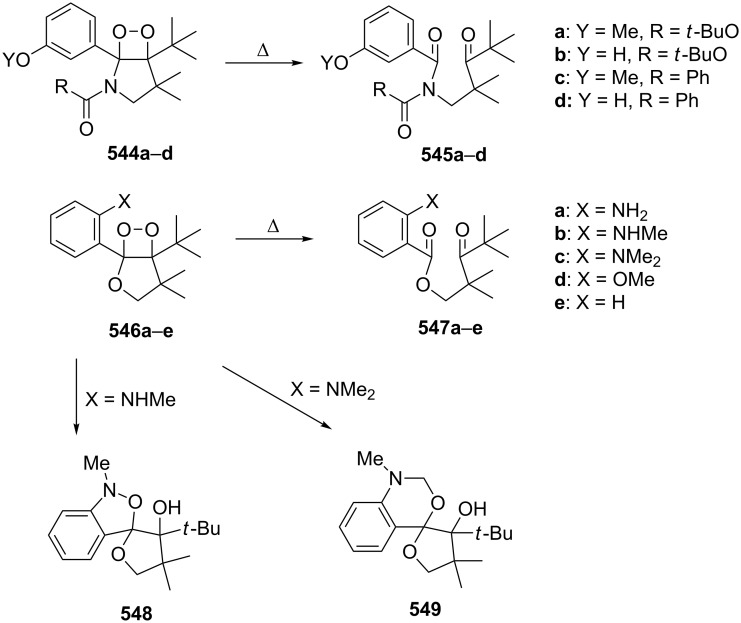
The thermal rearrangements of strained cyclic peroxides.

This difference is apparently attributable to the following two factors: (1) the lower nucleophilicity of the secondary hydroxy group compared to the tertiary hydroxy group; (2) the conformer, which would be suitably orientated towards the nucleophilic attack, is sterically unfavored in the case of R^2^ = H. At −20 °C, the transformation of **541d** into a conformational isomer occurs more slowly than the thermal decomposition giving **543d** (Scheme 157). Thermal rearrangements of strained cyclic peroxides **544a–d** and **546a–e** provide a versatile tool for the synthesis of carbonyl compounds **545a–d** and **547a–e** and heterocyclic systems **548** and **549** (Scheme 158) [498–499].

**Scheme 159:**
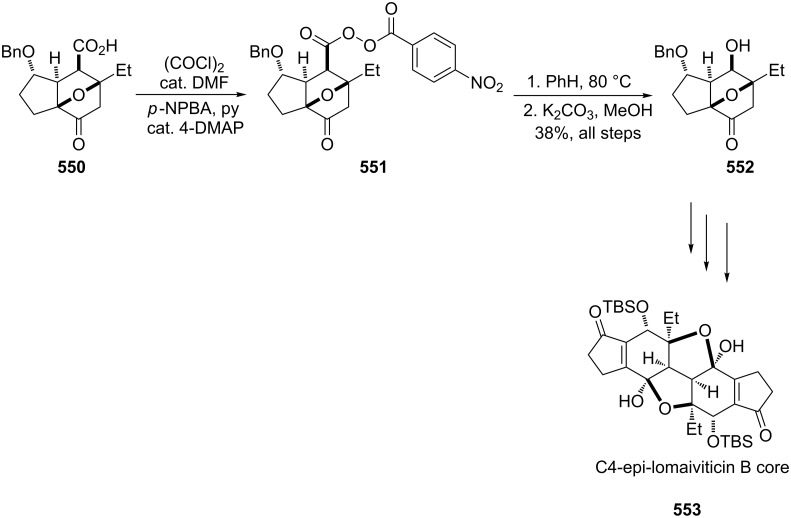
The thermal rearrangement of diacyl peroxide **551** in the synthesis of C4-*epi*-lomaiviticin B core **553**.

The thermal rearrangement of diacyl peroxide **551** was carried out in the synthesis of the C4-*epi*-lomaiviticin B core **553**. Diacyl peroxide **551** was prepared from *p*-nitroperbenzoic acid (*p*-NPBA) and the acid chloride of carboxylic acid **550**. An ionic Criegee-like rearrangement of peroxide **551** upon heating resulted in the corresponding acyl carbonate species. The reaction of MeOH with this acyl carbonate intermediate provided a single diastereomer of secondary carbinol **552** in 38% yield (Scheme 159) [500].

**Scheme 160:**
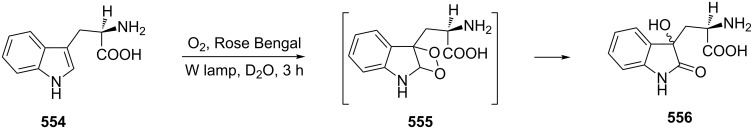
The ^1^O_2_ oxidation of tryptophan (**554**) and rearrangement of dioxetane intermediate **555**.

Two diastereoisomeric dioxindolylalanines **556** were identiﬁed after the ^1^O_2_ oxidation of tryptophan (**554**). Mechanistic investigations supported the dioxindolylalanine formation through a dioxetane intermediate **555** (Scheme 160) [501].

#### 2.5 Metal-catalyzed transformations of peroxides

This section focuses on transformations of peroxides under the action of the most representative metals used for these types of reactions: Fe(II), Co(II), Ru(II), and Pd(II).

**Scheme 161:**
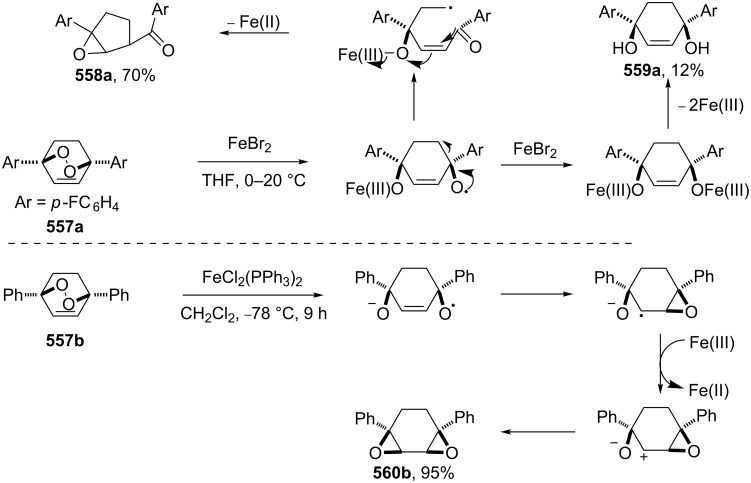
The Fe(II)-promoted cleavage of aryl-substituted bicyclic peroxides.

The Fe(II)-promoted activation of peroxides is believed to be involved in the antimalarial activity of a number of peroxides, including the natural product artemisinin. The understanding of the underlying mechanism of the Fe(II)-promoted cleavage of bicyclic peroxides is critical to the design and preparation of more efficient antimalarial peroxides. From this perspective, metal-catalyzed transformations of peroxides are of special interest. It was shown [502] that the reaction of fluorinated cyclic peroxide **557a** with FeBr_2_ in THF proceeds through an intermediate O-centered radical to form epoxy ketone **558a** and 1,4-diol **559a**. The reaction of **557b** with FeCl_2_(PPh_3_)_2_ in CH_2_Cl_2_ proceeds in a different manner through an intermediate O-centered radical to yield diepoxide **560b** (Scheme 161) [503].

**Scheme 162:**
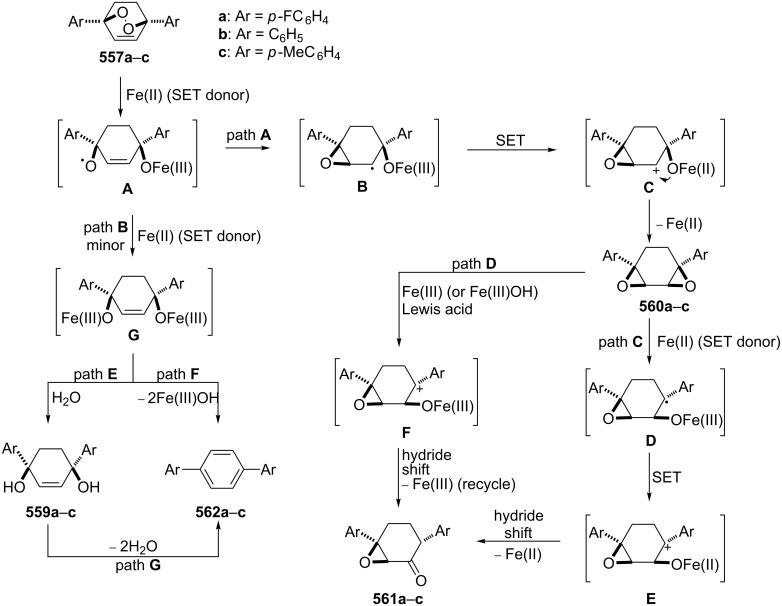
The proposed mechanism of the Fe(II)-promoted rearrangement of **557a–c**.

In a related study investigating the reaction of **557а–с** with FeBr_2_, bis-epoxides **560а–с** and epoxy ketones **561а–с** were obtained as the major products (Table 29) [504] and the proposed mechanism of the rearrangement of **557a–c** is presented in Scheme 162.

**Table 29:** Transformation of endoperoxides **557а–с** under the action of FeBr_2_.

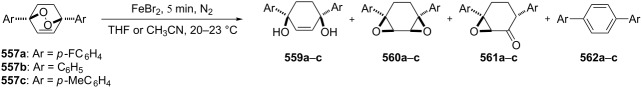						

Substrate	Solvent	Conversion, %	Yield, %			

			559	560	561	562

**557a**	THF	100	<1	52	26	<1
**557b**	THF	100	<1	52	20	3
**557c**	THF	100	20	0	65	3
**557a**	CH_3_CN	95	<1	69	<1	2
**557b**	CH_3_CN	100	<1	73	<1	2
**557c**	CH_3_CN	91	0	50	22	2

Both in THF and CH_3_CN, the intermediate O-centered radical **A** is generated via an electron transfer from Fe(II) to **557a–c**. The transformation of intermediate **A** can proceed through two different pathways. The first involves the intramolecular addition of an O-centered radical to the double bond in radical **A** to form C-centered radical **B** (path **A**). The second pathway involves an electron transfer from Fe(II) to radical **A** to give intermediate **G** (minor path **B**). The intramolecular electron transfer in intermediate **B** results in the formation of carbocation **C** followed by the formation of diepoxide **560a–c** and concomitant elimination of Fe(II). The generation of epoxy ketone **561a–c** from **560a–c** can occur through paths **C** and **D**. Paths **E** and **F** apparently give rise to 1,4-diol **559a–c** and diarylbenzene **562a–c**, respectively, from intermediate **G**.

**Scheme 163:**
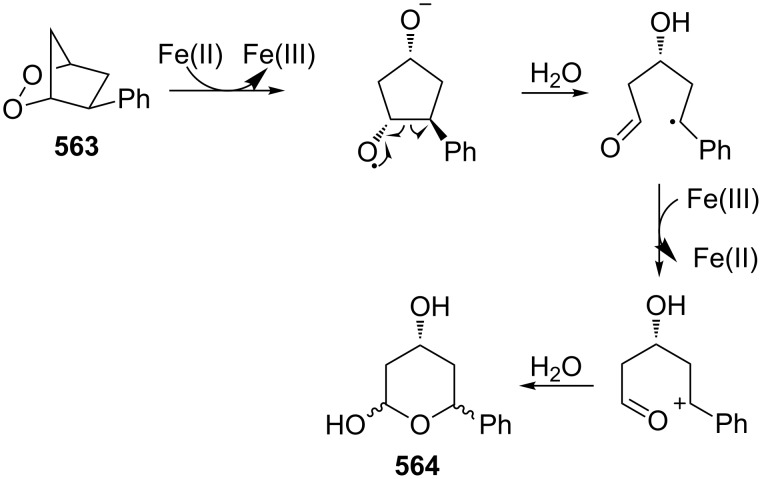
The reaction of dioxolane **563** with Fe(II) sulfate.

The reaction of dioxolane **563** with Fe(II) sulfate produces an O-centered radical, and the β-scission of the latter gives a C-centered radical, the oxidation and further cyclization of which yields **564** (Scheme 163) [505].

**Scheme 164:**
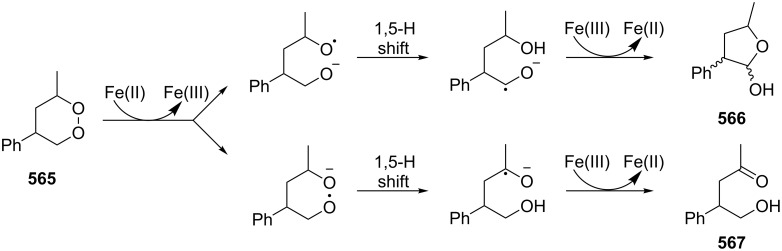
Fe(II)-promoted rearrangement of 1,2-dioxane **565**.

The monocyclic 1,2-dioxane **565**, as opposed to related dioxolane **563**, decomposes under the action of Fe(II) with exclusive formation of a 1:1 mixture of products **566** and **567**. This is attributed to the fact that the reaction proceeds through 1,5-hydrogen transfer, while β-scission does not occur (Scheme 164) [505].

**Scheme 165:**
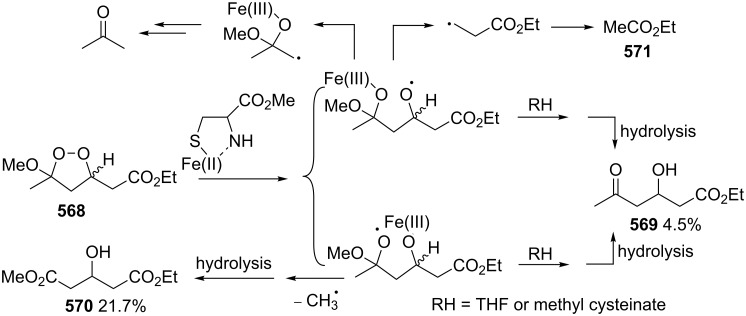
Fe(II) cysteinate-promoted rearrangement of 1,2-dioxolane **568**.

The reaction of Fe(II) cysteinate with dioxolane **568** produced compounds **569** and **570**, which were isolated from the reaction mixture. The formation of methyl acetate **571** was confirmed by GC analysis of the reaction mixture before work-up (Scheme 165) [506].

**Scheme 166:**
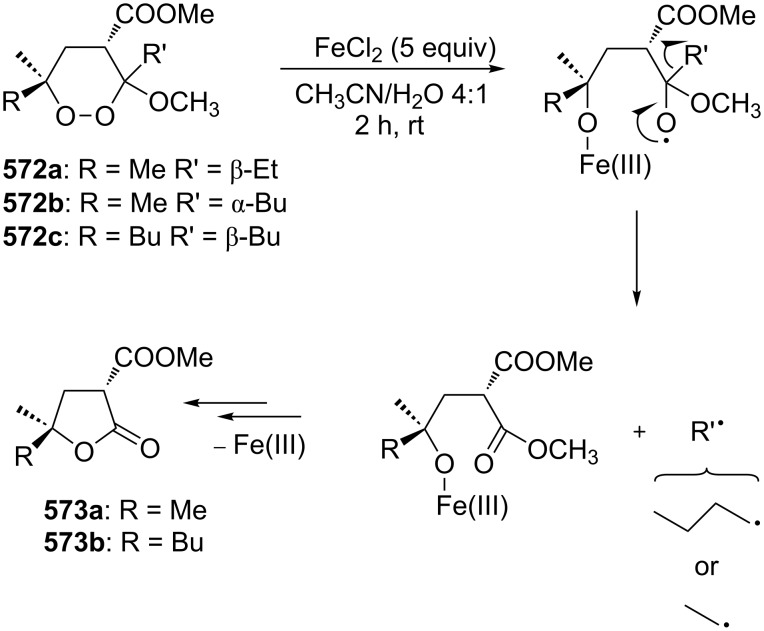
The transformation of 1,2-dioxanes **572a–c** under the action of FeCl_2_.

The reaction of 1,2-dioxanes **572a–c** with FeCl_2_ is accompanied by the formation of lactones **573a**,**b**, which were isolated in the individual state (Scheme 166) [507].

**Scheme 167:**
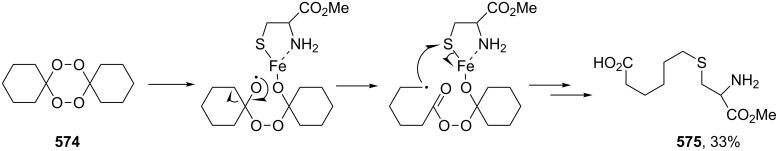
Fe(II) cysteinate-promoted transformation of tetraoxane **574**.

The reaction of synthetic tetraoxane **574** with Fe(II) cysteinate affords a complex mixture of products. Only one product, **575**, could be isolated from the mixture and identified. This was the first work, where the Fe(II)-promoted cleavage of 1,2,4,5-tetraoxane was investigated (Scheme 167) [508].

The hypothesis that this difference in the structure of the reaction products is associated with the rearrangement of intermediate endoperoxides gave impetus to research on the reaction of endoperoxides with transition metal derivatives. It was found that the catalytic rearrangement of endoperoxides using cobalt *meso*-tetraphenylporphyrin occurs in high yield. Therefore, this is an efficient approach to the synthesis of *syn*-1,2:3,4-diepoxides from 1,3-dienes under mild conditions. Table 30 summarizes the results of the cobalt(II) tetraphenylporpyrin-catalyzed rearrangement of endoperoxides **576** [509], **578** [510], **580** [511], **582** [512], **584** [353], **586** [513], **588** [514], **590**, **592** [515], **594** [516], **596** [517], and **598** [518] which afforded products structurally similar to the diepoxides prepared by thermal rearrangement of endoperoxides (Table 25). All rearrangements were stereospecific and yielded only the *syn*-diepoxides.

**Table 30:** CoTPP-catalyzed rearrangement of endoperoxides.

Substrate	Product	Yield, %

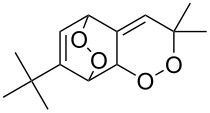 **576**	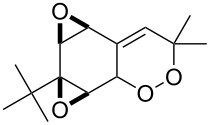 **577**	50
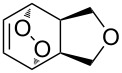 **578**	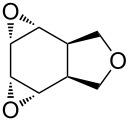 **579**	84
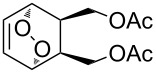 **580**	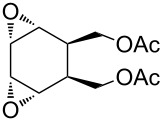 **581**	80
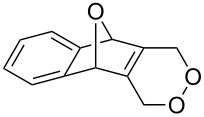 **582**	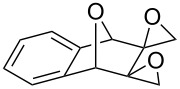 **583** or 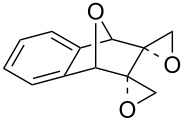	75
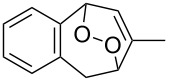 **584**	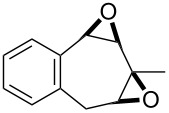 **585**	45
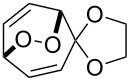 **586**	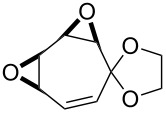 **587**	61
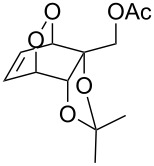 **588**	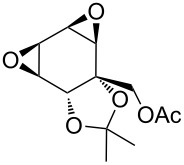 **589**	96
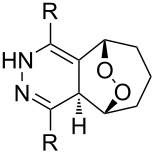 R = COOCH_3_ **590**	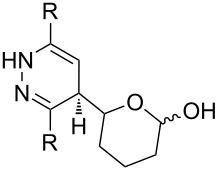 **591**	83
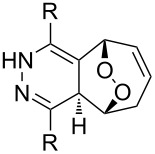 R = COOCH_3_ **592**	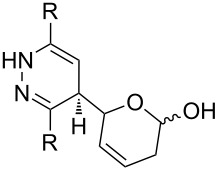 **593**	55
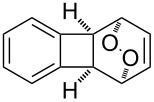 **594**	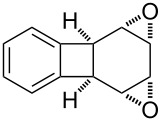 **595**	90
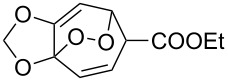 **596**	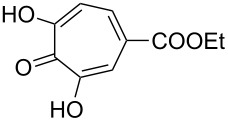 **597**	60
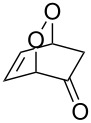 **598**	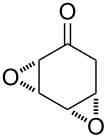 **599**	50

**Scheme 168:**
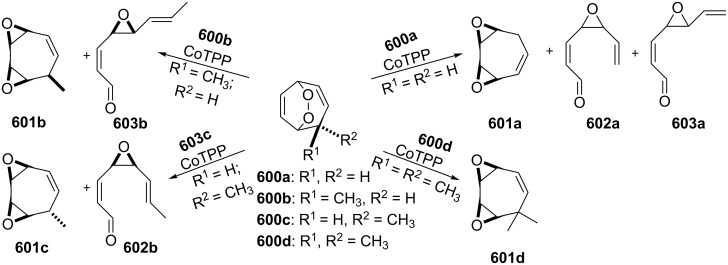
The CoTPP-catalyzed transformation of bicyclic endoperoxides **600a–d**.

The study of the CoTPP-catalyzed transformation of bicyclic endoperoxides containing non-strained diene moieties demonstrated that the formation of epoxides can be accomplished in yields up to 90–100%, while the side reaction giving epoxy ketones is suppressed. A detailed study on the CoTPP-catalyzed reaction of **600a** showed that this reaction affords, in addition to the expected diepoxide **601a**, two isomeric epoxy aldehydes **602a** and **603a**. The reaction of bicyclic endoperoxides **600b**,**c** gives, instead of the expected epoxides **601b**,**c**, exclusively epoxy aldehydes **602b**, **603b** and the reaction of endoperoxide **600d** produces solely the diepoxide **601d** (Scheme 168) [519].

**Scheme 169:**
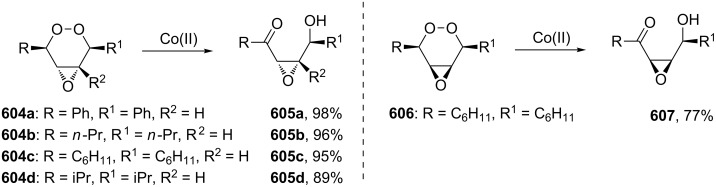
The CoTPP-catalyzed transformation of epoxy-1,2-dioxanes.

The reaction of epoxy-1,2-dioxanes **604a–d** and **606** with Co(II) complexes affords 4-hydroxy-2,3-epoxy ketones **605a**–**d** and **607** in good yields (Scheme 169) [364]. Possibly the selectivity towards the hydroxyketones formation is provided by means of cobalt ions interaction. The obtained compounds are useful synthons in organic synthesis.

**Scheme 170:**
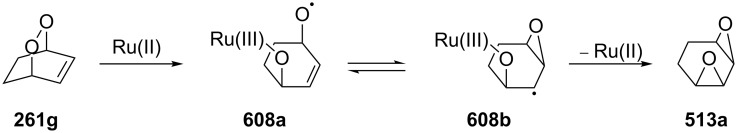
The Ru(II)-catalyzed reactions of 1,4-endoperoxide **261g**.

The Ru(II)-catalyzed reactions of 1,4-endoperoxide **261g** involves the formation of intermediate radicals **608a**,**b**, the structures of which differ from that of the radicals generated by photolysis or thermal decomposition. Ruthenium ions have a considerable effect on the stability and reactivity of radicals, resulting in the selective transformation of peroxides under mild conditions. The reactivity also substantially depends on steric factors (Scheme 170) [503,520–521].

**Scheme 171:**
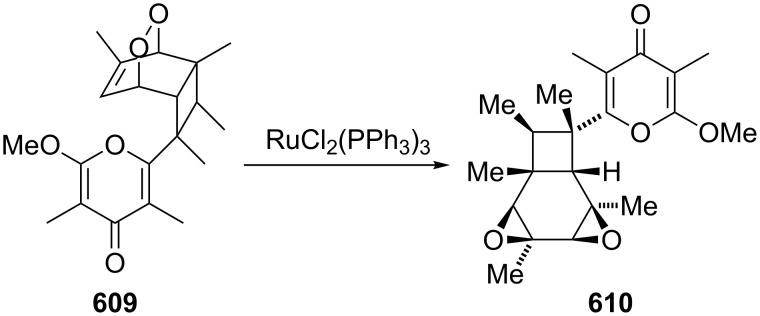
The Ru(II)-catalyzed transformation as a key step in the synthesis of elyiapyrone A (**610**) from 1,4-endoperoxide.

The Ru(II)-catalyzed transformation of 1,4-endoperoxide **609** is used as a key step in the synthesis of the natural compound, elyiapyrone A (**610**) (Scheme 171) [522–523].

Transformations of endoperoxides catalyzed by variable-valence metals are well studied for metals such as Cu(II), Fe(II), or Co(II), which can initiate the reaction through a one-electron oxidation–reduction mechanism. The decomposition of endoperoxides catalyzed by Ru(II)phosphine complexes also belongs to this type of reaction and the decomposition produces diepoxides as the major products.

The reactions of endoperoxides with Pd(0) proceed through different pathways. Thus, bicyclic 2,3-saturated 1,4-endoperoxides **611a–d** are transformed into the corresponding 4-hydroxyketones and 1,4-diols. Bicyclic 2,3-unsaturated 1,4-endoperoxides **530**, **261g**, **263** produce 4-hydroxyenones, 1,4-diols, and diepoxides. Monocyclic endoperoxides **611e–g** are transformed into enones, 1,4-diols, 1,4-diketones, or furan derivatives (Table 31) [524–525].

**Table 31:** Pd(PPh_3_)_4_-catalyzed transformation of 1,4-endoperoxides.

Endoperoxide	Temperature, °C (time, h)	Products, %			

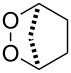 **611a**	17 (3)	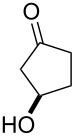 **612a**, 41	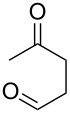 **613a**, 29		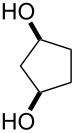 **614a**, 20
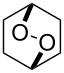 **611b**	60 (5)	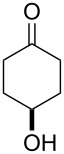 **612b**, 49	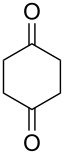 **613b**, 3		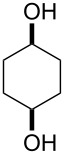 **614b**, 37
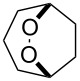 **611c**	60 (10)	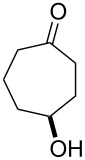 **612c**, 62	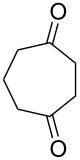 **613c**, 13		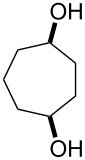 **614c**, 25
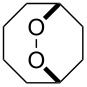 **611d**	65 (15)	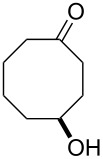 **612d**, 73			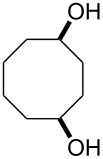 **614d**, 23
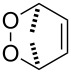 **530**	4 (20)	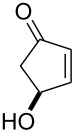 **612e**, 54			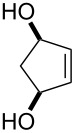 **614e**, 16
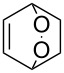 **261g**	50–60 (5)	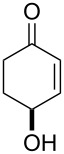 **262g**, 42		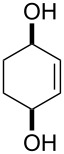 **614f**, 32	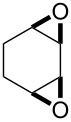 **513a**, 9
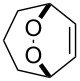 **263**	60 (29)	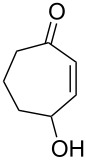 **612f**, 45	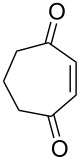 **613d**, 10	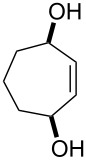 **614g**, 17	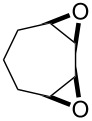 **513b**,28
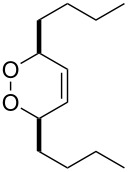 **611e**	60 (39)	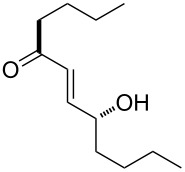 **612g**, 34		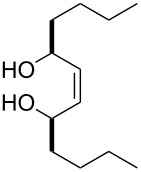 **614h**, 59	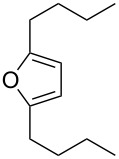 **615**, 5
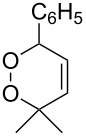 **611f**	100 (15)	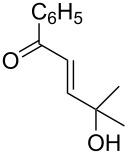 **612h**, 66			
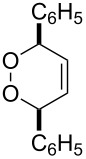 **611g**	70 (12)	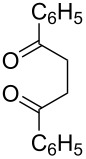 **612i**, 74			

The reactivity of bicyclic substrates depends on the carbon-ring size. Strained 1,4-endoperoxide derivatives are readily decomposed under the action of Pd(PPh_3_)_4_ at room or elevated temperatures, whereas substrates containing larger rings require more severe conditions. Monocyclic substrates are less reactive than bicyclic endoperoxides and require even more harsh conditions.

### 3 Rearrangements and related processes of important natural and synthetic peroxides

#### 3.1 Antimalarial, antiparasitic, and antitumor peroxides

**Scheme 172:**
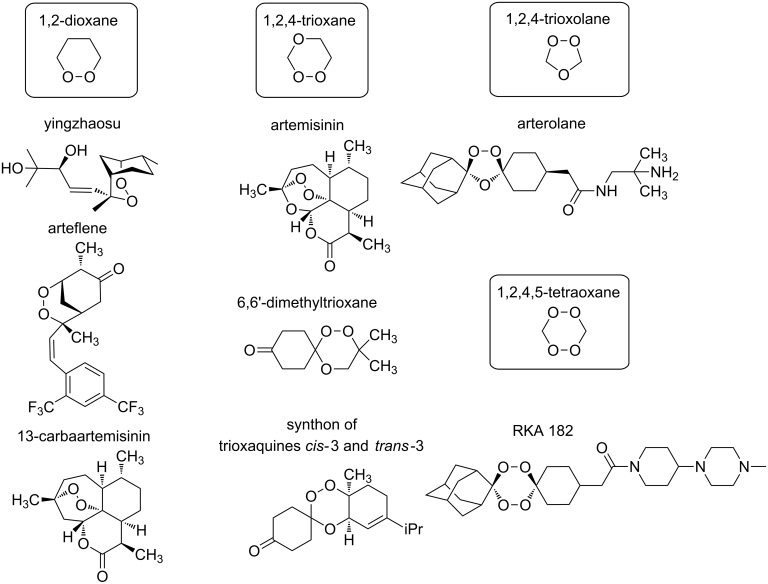
Peroxides with antimalarial activity.

The extensive development of the chemistry of organic peroxides has been stimulated largely by the isolation of the antimalarial agent artemisinin from leaves of the annual wormwood *Artemisia annua* in 1972. The structural identification showed that artemisinin contains a cyclic endoperoxide moiety (1,2,4-trioxane ring), which plays a key role in its antimalarial activity [526–527]. The highly reactive and unusual chemical structure, in addition to low yields isolated from natural sources gave impetus to the development of total synthesis methods of artemisinin. Several routes towards the total synthesis of this compound were elaborated and several semisynthetic derivatives were prepared [12,16,528–533]. The high costs of these products stimulated the search for alternative peroxides, which are synthetically easier accessible and less expensive compared with the natural and semisynthetic structures. It was shown that 1,2-dioxolanes [35], 1,2-dioxanes [40], 1,2,4-trioxolanes [534–536], 1,2,4-trioxanes [44], and 1,2,4,5-tetraoxanes [537] exhibit antimalarial activity, which was sometimes higher than that of the parent artemisinin (Scheme 172). As a milestone of this research, arterolane, a fully synthetic 1,2,4-trioxolane was discovered and in 2012, the arterolane-based drug synriam was approved to the market.

Although artemisinin has been used in medicine for about three decades, the mechanism of its action remains unclear [538–539]. Two main theories of its antiparasitic action are assumed. In accordance with one theory, the endoperoxide bond is reduced by means of iron ions leading to the formation of oxygen-centered radicals, which are responsible for the initiation of oxidative stress in infected erythrocytes. An alternative theory proposes that specific parasites’ proteins or heme are alkylated by carbon-centered radicals derived from the peroxide [540–541]. In infected human erythrocytes, malaria parasites digest more than 70% of the hemoglobin with formation of globin and heme. After the hydrolysis of globin, the resulting amino acids are used by the parasites for protein synthesis. Malaria parasites detoxify the toxic heme via a heme polymerization process with preparation of hemozoin, which exists in the crystalline form. Parasite metalloproteins, superoxide dismutase and ferredoxin, use a small part of the host’s iron for their construction. In such a manner parasite cells always contain heme iron and non-heme iron, allows for the interaction with artemisinin or other peroxides [542].

**Scheme 173:**
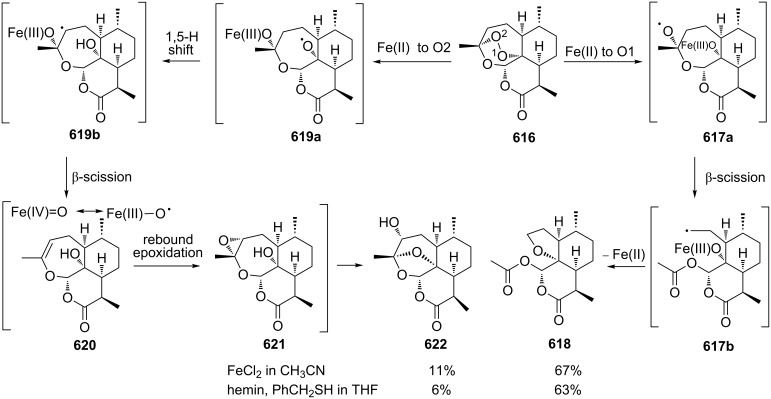
The interaction of iron ions with artemisinin (**616**).

Numerous studies on the interaction of iron ions with artemisinin (**616**) demonstrated that Fe(II) promotes the O–O-bond cleavage via two paths. Thus, Fe(II) may bind to either O1 or O2 in artemisinin (Scheme 173) [542–551]. The interaction of Fe(II) with O1 gives rise to an intermediate oxy radical **617a**, which undergoes β-scission to form the primary C-centered radical **617b**. The subsequent elimination of Fe(II) is accompanied by the formation of compound **618** containing a tetrahydrofuran ring. The pathway involving the interaction of Fe(II) with O2 affords the O-centered radical **619a**. A subsequent [1,5]-H shift results in the formation of the secondary C-centered radical **619b**, and the β-scission of the latter produces vinyl ester **620**, which can be epoxidated by the resulting high-valent iron-oxo species. Epoxide **621** is finally cyclized to hydroxydeoxoartemisinin **622**. The formation of **618** and **622** is evidence in favor of the proposed two pathways of the Fe(II)-promoted transformation of artemisinin. The highly reactive intermediates **617** and **619** apparently lead to the damage of some parasite biomolecules [552].

**Scheme 174:**
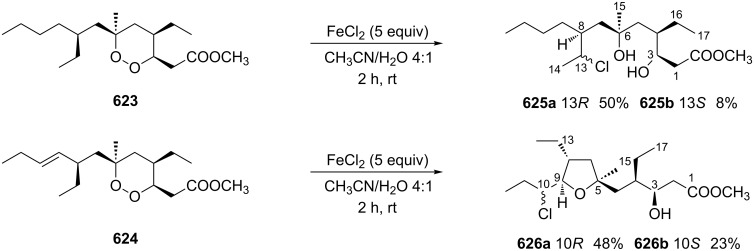
The interaction of FeCl_2_ with 1,2-dioxanes **623**, **624**.

The 1,2-dioxanes **623** and **624** exhibiting antimalarial activity were isolated from the Caribbean sponge *Plakortis simplex* and their reactions with Fe(II) result in compounds **625a**,**b** and **626a**,**b**, respectively (Scheme 174) [553].

**Scheme 175:**
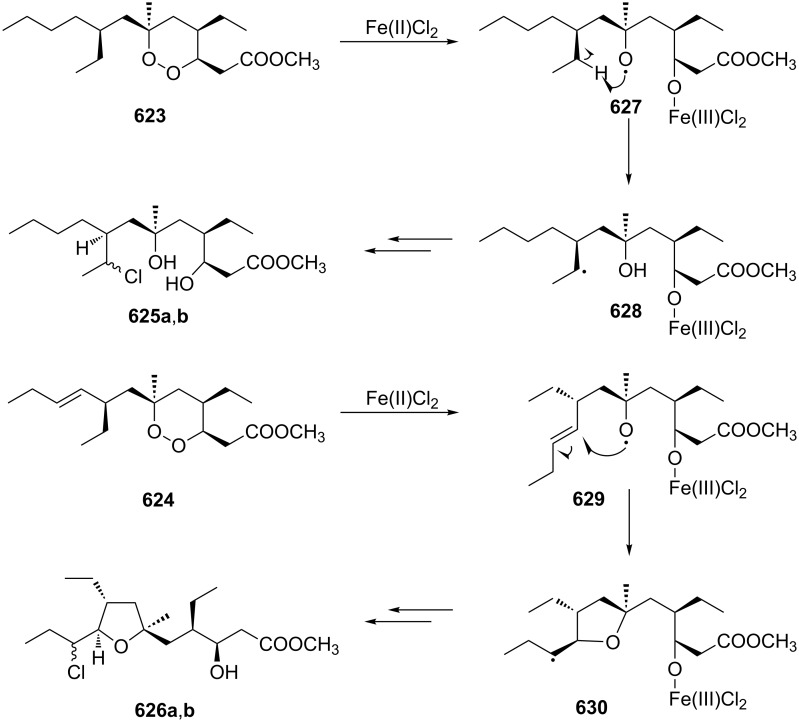
The mechanism of reaction **623** and **624** with Fe(II)Cl_2._

Scheme 175 shows the mechanism including the formation of oxygen radicals **627**, **629** from cyclic peroxides **623** and **624**. The 1,5-rearrangement of the latter produces the alkyl-side chain carbon-centered radicals **628**, **630**. The reaction of these toxic intermediates with parasite biomolecules determines the biological effect observed for 1,2-dioxanes **623** and **624** (Scheme 175).

**Scheme 176:**
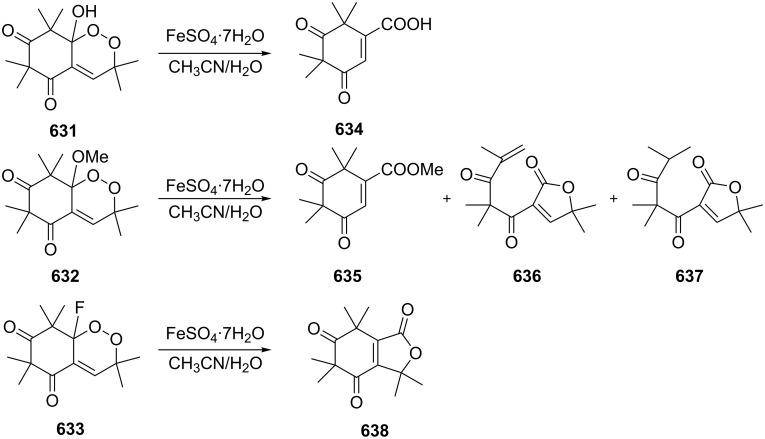
The reaction of bicyclic natural endoperoxides G3-factors **631–633** with FeSO_4_.

Depending on the nature of the substituents in close vicinity of the peroxide group, the bicyclic natural endoperoxides G3-factors **631–633** which are involved in plant defense and extracted from the leaves of *Eucalyptus grandis*, react with Fe(II) to form different types of products. For instance, treatment of the **631** with Fe(II)SO_4_, gives rise to **634** in 82% yield. On the other hand the reaction of **632** under the same reaction conditions affords three products **635**, **636**, and **637** in a 1:1:1 ratio. The fluorinated endoperoxide **633** gives exclusively **638** under these conditions (Scheme 176) [554–555].

**Scheme 177:**
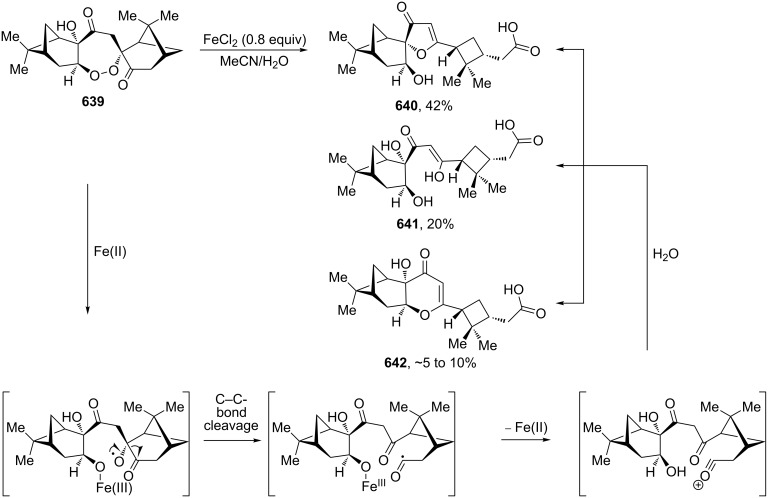
The transformation of terpene cardamom peroxide **639**.

In the reaction with Fe(II), the natural antimalarial terpene cardamom peroxide **639** isolated from *Amomum krervanh* Pierre (Siam cardamom) is transformed into acids **640**, **641**, and **642** (Scheme 177) [164].

**Scheme 178:**
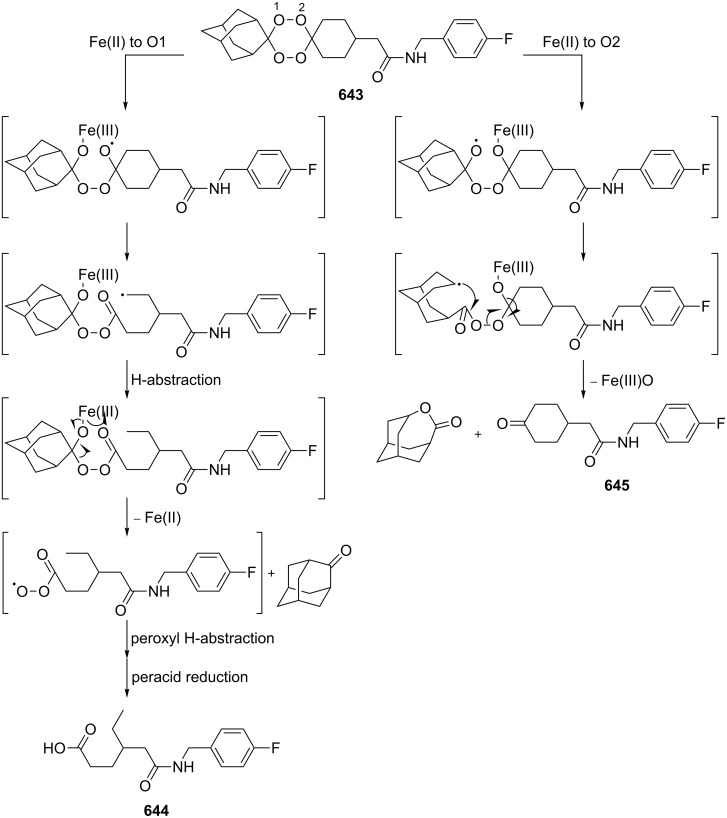
The different ways of the cleavage of tetraoxane **643**.

However, the cleavage of tetraoxane **643** gives two major products, namely **644** and **645**, in yields of 44% and 51%, respectively. The reaction mechanism based on the results of this study is shown in Scheme 178 [556].

**Scheme 179:**
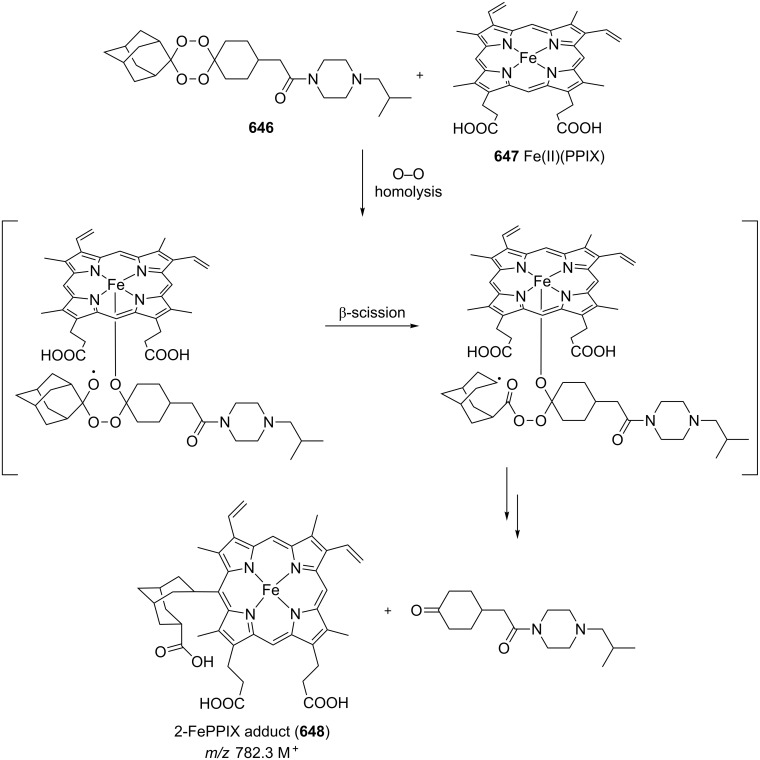
The LC–MS analysis of interaction of tetraoxane **646** with iron(II)heme **647**.

Presumably, in accordance with the direction from Fe(II) to O2, tetraoxane **646** interacts with iron(II) heme **647**. Starting heme **647** reacts within 30 min with formation of three products. The LC–MS study proved the formation of the covalent coupling product **648** formed from heme (mass 616) and the tetroxane-derived secondary C-centered radical. The molecular ion [M]^+^ of coupling product **648** was observed at *m/z* 782.3, which is consistent with the prediction (Scheme 179) [537,556].

Under similar conditions, the same alkylated heme adduct was obtained with trioxolanes [557]. Four peaks at *m*/*z* 782.3 were detected which were assigned to the four possible regioisomers of alkylated heme adduct **648** as reported for heme–artemisinin adducts [558]. Later, in an initial study dealing with monoclonal antibodies that recognize the alkylation signature (sum of heme and protein alkylation) of synthetic peroxides it was shown that the artemisinins alkylate proteins in *P. falciparum* [559].

All the above-mentioned transformations involve the homolytic O–O-bond cleavage resulting in the formation of an O-centered radical, which is followed by the rearrangement into a C-centered radical, as a key step. The subsequent transformation of the C-centered radical determines the structure of the final product.

**Scheme 180:**
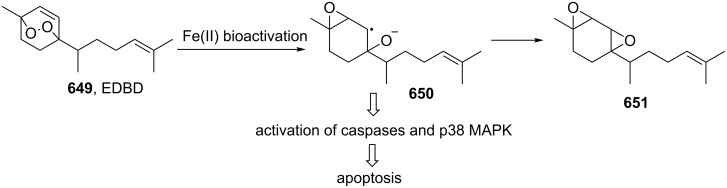
The rearrangement of 3,6-epidioxy-1,10-bisaboladiene (EDBD, **649**).

The peroxide, 3,6-epidioxy-1,10-bisaboladiene (EDBD, **649**), isolated from wild plants, *Cacalia delphiniifolia* and *Cacalia hastata*, possesses cytotoxicity against the human promyelocytic leukemia cell line HL60. It was shown that the mechanism of biological activity of EDBD involves a rearrangement with formation of an unstable C-centered radical intermediate **650**, followed by its transformation into product **651** (Scheme 180) [560].

#### 3.2 Rearrangement of lipid peroxides

**Scheme 181:**
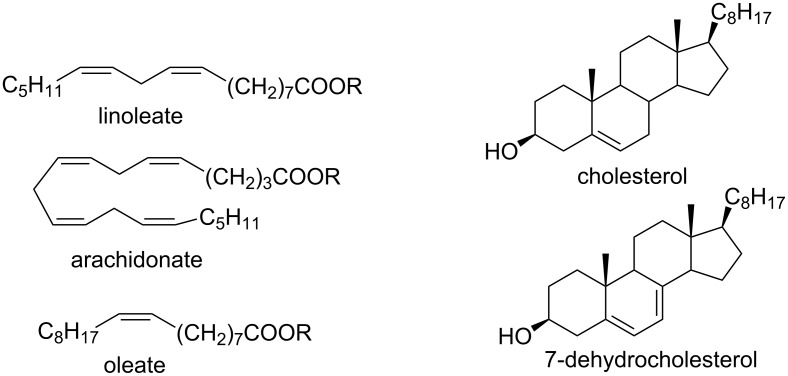
Easily oxidized substrates.

Lipids contained in cell membranes maintain the structure and control of the vital functions of cells. Lipids are the targets of the reactions with reactive oxygen species (ROS) such as various oxygen-centered radicals, which play a key role in several pathological states [561]. Compounds containing double bonds, polyunsaturated fatty acids and esters, cholesterol and its derivatives easily undergo oxidation by action of oxygen-centered radicals (Scheme 181) [562–563].

**Scheme 182:**
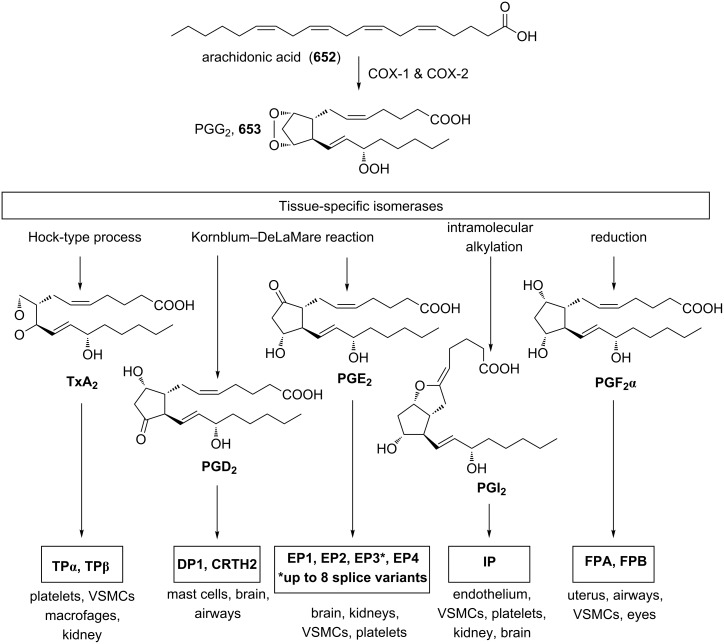
Biopathway of synthesis of prostaglandins.

Rearrangements of organic peroxides play an important role in such biological processes as the synthesis of prostaglandins from fatty acids. Prostaglandins are physiologically active substances produced by the reaction of arachidonic acid (**652**) with cyclooxygenase (COX) isoenzymes. Prostaglandin G_2_ (PGG_2_, **653**) containing an endoperoxide fragment undergoes transformations mediated by a series of specific isomerases and synthases with production of PGE_2_, PGI_2_, PGD_2_, PGF_2_, and T_X_A_2_ (Scheme 182) [564–567].

**Scheme 183:**
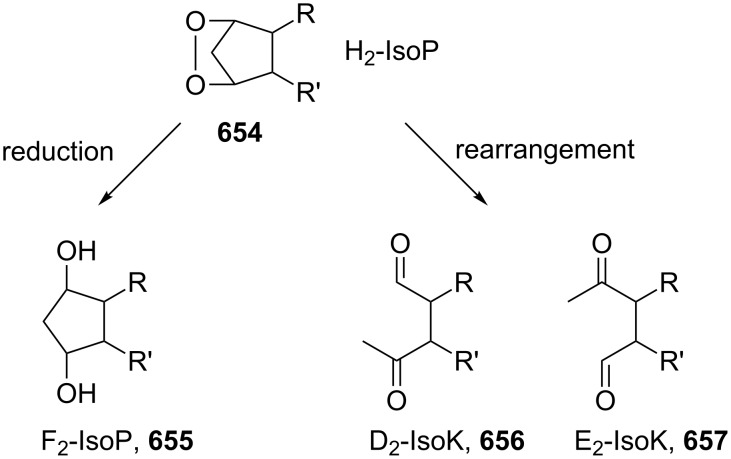
The reduction and rearrangements of isoprostanes.

The formation of the metabolites isoprostanes, neuroprostanes, phytoprostanes, and isofurans **655–657** from fatty acids under autoxidative conditions in vivo involves both the reduction of peroxides and their rearrangements (Scheme 183). These compounds proved to be widespread in nature. Compounds **655**–**657** display significant biological activities, and the isoprostanes are currently the most reliable indicators of oxidative stress [568–570].

**Scheme 184:**
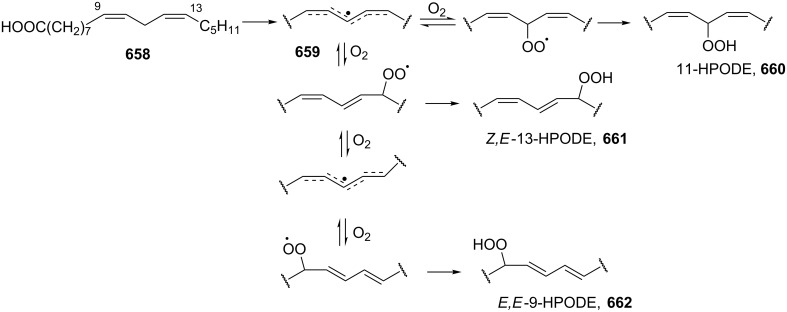
The partial mechanism for linoleate **658** oxidation.

One of the essential fatty acids, linoleic acid, contains a homoconjugated diene fragment, which is responsible for a specific peroxidation mechanism without the formation of cyclic peroxides. In addition to linoleic acid, its esters are present in the human circulating low-density lipoprotein (LDL). For this reason, the oxidation of linoleic acid esters is of special biomedical interest [566]. A mechanism for linoleate (**658**) oxidation, which involves hydroperoxyoctadecadienoates (HPODE, **660–662**) preparation, is presented in Scheme 184. The first step of the oxidation process is the formation of the carbon-centered pentadienyl radical **659**. The reaction of **659** with O_2_ produces three peroxyl radicals, one of them having a nonconjugated diene part with the oxygen at C-11 position. The two other radicals have *Z*,*E*- and *E,E-*conjugated diene parts with oxygen substituents at the C-9 and C-13 positions. These peroxyl radical intermediates after abstracting hydrogen atoms transform to the hydroperoxyoctadecadienoates (HPODE, **660–662**) [570].

**Scheme 185:**
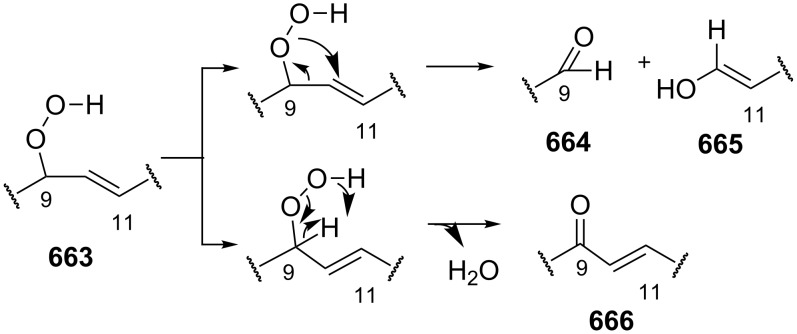
The transformation of lipid hydroperoxide.

The Hock cleavage mechanism is a possible route to transform lipid hydroperoxide **663** into smaller carbonyl compounds **664–666**, although this transformation seems to occur only in the presence of photosensitizers (Scheme 185) [571].

**Scheme 186:**
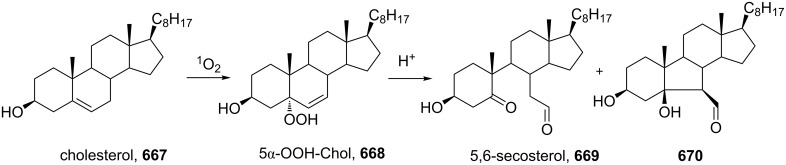
The acid-catalyzed cleavage of the product from free-radical oxidation of cholesterol (**667**).

In mammalian tissues and cells, cholesterol is found to a large extent. One of the main cholesterol functions represents to maintaining the stability of plasma membranes. The oxidation of cholesterol by means of free radical particles is responsible for the initiation of a range of pathological conditions [572–573]. Many processes including the rearrangement of intermediately formed peroxides accompany the oxidation of cholesterol. The major product of ^1^O_2_ oxidation of cholesterol (**667**), cholesterol 5α-hydroperoxide (**668**), readily forms 5,6-secosterol ketoaldehyde **669** and the product of its intramolecular aldolization **670** through an acid-catalyzed (Hock) cleavage of the C5–C6 bond in **668** (Scheme 186) [67].

#### 3.3 Rearrangement of dioxygenase enzyme–substrate systems

**Scheme 187:**
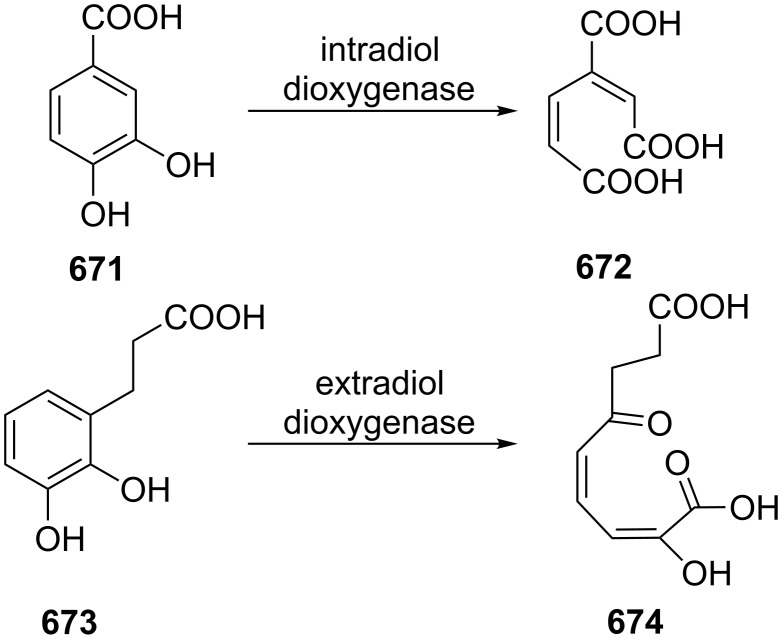
Two pathways of catechols oxidation.

A useful chemical property of most soil bacteria concludes in their capability to oxidize aromatic compounds. This multistep process depends on the structure of dioxygenase enzymes, which utilize molecular oxygen for oxidation [574]. This oxidation has attracted much attention as a green chemistry approach for the conversion of aromatic compounds to water-soluble products and for degradation of lignin [575–576]. The ring cleavage of 1,2-dihydroxybenzene (catechol) is likely the most thoroughly studied reaction which is catalyzed by iron-dependent catechol dioxygenase enzymes [577–579]. The oxidation of catechols **671** and **673** by two types of enzymes – intradiol dioxygenase and extradiol dioxygenase – affords 3-carboxyhexa-2,4-dienedioic acid (**672**) and 2-hydroxy-6-ketonona-2,4-dienoic acid (**674**) (Scheme 187) [580–581].

**Scheme 188:**
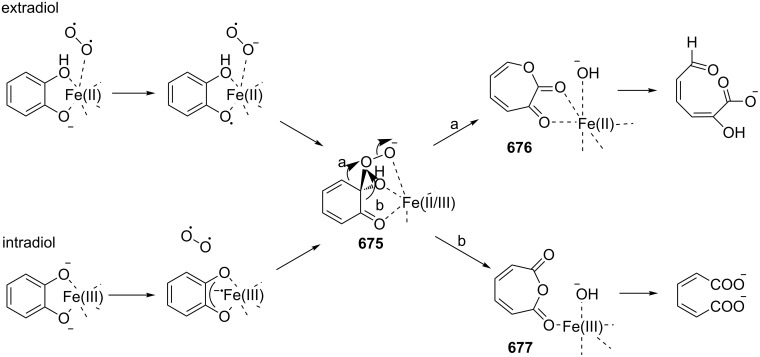
Criegee-like or Hock-like rearrangement of the intermediate hydroperoxide **675** in dioxygenase enzyme–catechol system.

A key step in the cleavage of the aromatic ring is the oxygen-atom insertion into the C–C-double bond as the result of a Criegee-like or Hock-like intermediate rearrangement [582–583]. It was demonstrated that, despite the different mechanisms of the initial step of the substrate/molecular oxygen activation, both reactions produce hydroperoxide **675** as the intermediate. This hydroperoxide undergoes Criegee-like or Hock-like rearrangement through different pathways. Intradiol dioxygenase catalyzes the 1,2-acyl migration (path **B**) and the formation of an intermediate anhydride **677**. On the other hand, extradiol dioxygenase catalyzes the 1,2-migration of the alkenyl moiety (path **A**) through the intermediate formation of lactone **676** (Scheme 188) [584].

Therefore, the catalyst for the O–O-bond cleavage in the Criegee-like intermediate determines the regioselectivity of the catechol oxidation.

**Scheme 189:**
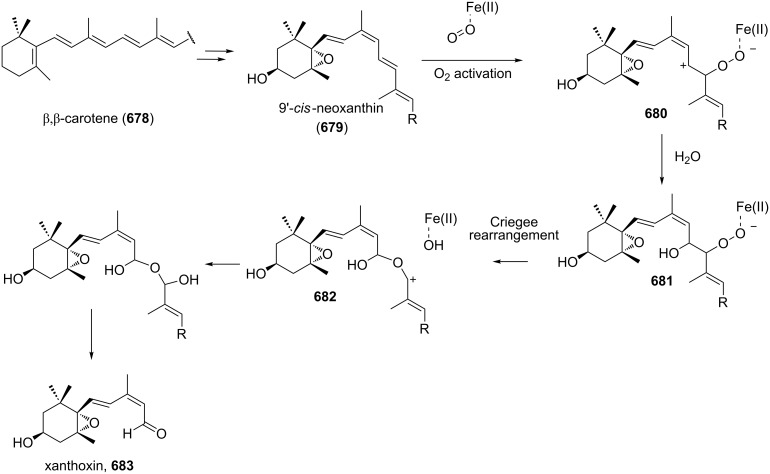
Carotinoides **679** cleavage by carotenoid cleavage dioxygenases.

A similar rearrangement of the Criegee intermediate with the cleavage of the С–С bond occurs in oxidative cleavage of natural organic pigments, carotinoides **679** by carotenoid cleavage dioxygenases (Scheme 189) [585–586].

In this section, we considered rearrangements of the most important natural and synthetic peroxides, which proceed or can take place in biological systems. Apparently, there are is a much larger number of biological processes, involving rearrangements of peroxides, which has to be discovered and studied in the future.

## Conclusion

The rearrangements of organic peroxides and related processes are covered in the literature in hundreds of publications and several specialized reviews. However, these reviews are limited in scope, narrow in their approach, and do not provide an overall picture of this field of chemistry. The present review is the first to offer a complex analysis of the available data on rearrangements of peroxides published in the last 15−20 years with an excursion to the history of the discovery of particular reactions and transformations. The rearrangements and related processes are classified according to the type of the catalysts used: acid- and base-catalyzed processes, reactions catalyzed by variable-valence metals, photochemical and thermal action. Special emphasis is drawn to current trends in the performance and application of rearrangements of organic peroxides, such as asymmetric synthesis, organocatalysis, and the use of transition metal-peroxo complexes for the preparation of compounds interesting for pharmacological applications. The published data summarized in the review provide, for the first time, an insight into the common and different features of the reaction mechanisms and allow predicting experimental and structural requirements for performing rearrangements with specified results. An analysis of the published data shows that there are numerous new and unnamed processes related to name reactions. The development and investigation of these processes are apparently the future of peroxide chemistry.

Rearrangements of organic peroxides are the key steps in processes such as the Baeyer−Villiger, the Criegee and Hock reactions, the Kornblum−DeLaMare rearrangement, and Dakin and Elbs oxidation reactions. These reactions are widely used in chemistry: The Baeyer−Villiger oxidation is widely used for the synthesis of esters and lactones. The Criegee reaction is employed to transform tertiary alcohols into ketones and aldehydes. The Kornblum−DeLaMare rearrangement is an important tool in the synthesis of γ-hydroxy enones. The Dakin oxidation is applied in the synthesis of phenols from arylaldehydes or aryl ketones and the Elbs persulfate oxidation is used to prepare hydroxyphenols from phenols.

The comprehensive analysis of the published data makes the knotty term "peroxide rearrangement” more exact. Two types of processes are actually included under the term "peroxide rearrangement”: processes that fall under the definition of a classical rearrangement, resulting in the formation of a compound of isomeric structure, and processes, in which the O–O-bond cleavage is followed by the rearrangement of one of the resulting fragments.

The pathways of peroxide rearrangements mainly depend on the type of the catalysts used, the reaction conditions, and the structure of the starting peroxide. Rearrangements can be accompanied by a homolytic or heterolytic O–O-bond cleavage, through the formation of a carbocation (e.g., the Criegee rearrangement), a carbanion (e.g., the Kornblum−DeLaMare rearrangement), or an O-centered radical (e.g., the Wieland rearrangement or rearrangements promoted by variable-valence metals).

In recent years, there has been a growing interest in organic peroxides as a base for the design of antiparasitic and antitumor agents, which led to an extensive search for new classes of peroxides. New compounds and new structural classes play a key role in the development of the chemistry of rearrangements and the performance of related useful transformations of peroxides.
